# Association of genetic risk and lifestyle with pancreatic cancer and their age dependency: a large prospective cohort study in the UK Biobank

**DOI:** 10.1186/s12916-023-03202-0

**Published:** 2023-12-08

**Authors:** Liangtang Zeng, Zhuo Wu, Jiabin Yang, Yu Zhou, Rufu Chen

**Affiliations:** 1 School of Medicine, South China University of Technology, Guangzhou, Guangdong Province, China; 2 Department of Pancreatic Surgery, Department of General Surgery, Guangdong Provincial People’s Hospital (Guangdong Academy of Medical Sciences), Southern Medical University, Guangzhou, Guangdong Province, China

**Keywords:** Pancreatic cancer, Polygenic risk score, Healthy lifestyle, Prospective study, UK Biobank

## Abstract

**Background:**

Pancreatic cancer (PC) is influenced by both genetic and lifestyle factors. However, further research is still needed to comprehensively clarify the relationships among lifestyle, genetic factors, their combined effect on PC, and how these associations might be age-dependent.

**Methods:**

We included 340,631 participants from the UK Biobank. Three polygenic risk score (PRS) models for PC were applied, which were derived from the previous study and were categorized as low, intermediate, and high. Two healthy lifestyle scores (HLSs) were constructed using 9 lifestyle factors based on the World Cancer Research Fund/American Institute of Cancer Research (WCRF/AICR) lifestyle score and the American Cancer Society (ACS) guidelines and were categorized as unfavorable, intermediate, and favorable. Data were analyzed using Cox proportional hazards models.

**Results:**

There were 1,129 cases of incident PC during a median follow-up of 13.05 years. Higher PRS was significantly associated with an increased risk of PC (hazard ratio [HR], 1.58; 95% confidence intervals [CI], 1.47–1.71). Adhering to a favorable lifestyle was associated with a lower risk (HR, 0.48; 95% CI, 0.41–0.56). Participants with an unfavorable lifestyle and a high PRS had the highest risk of PC (HR, 2.84; 95% CI, 2.22–3.62). Additionally, when stratified by age, a favorable lifestyle was most pronounced associated with a lower risk of PC among participants aged ≤ 60 years (HR, 0.35; 95% CI, 0.23–0.54). However, the absolute risk reduction was more pronounced among those aged > 70 years (ARR, 0.19%, 95% CI, 0.13%–0.26%). A high PRS was more strongly associated with PC among participants aged ≤ 60 years (HR, 1.89; 95% CI, 1.30–2.73). Furthermore, we observed a significant multiplicative interaction and several significant additive interactions.

**Conclusions:**

A healthy lifestyle was associated with a lower risk of PC, regardless of the participants' age, sex, or genetic risk. Importantly, our findings indicated the age-dependent association of lifestyle and genetic factors with PC, emphasizing the importance of early adoption for effective prevention and potentially providing invaluable guidance for setting the optimal age to start preventive measures.

**Supplementary Information:**

The online version contains supplementary material available at 10.1186/s12916-023-03202-0.

## Background

Pancreatic cancer (PC) remains one of the deadliest cancers and has now overtaken breast cancer as the third leading cause of cancer-related deaths in the United States [1, 2]. Even more concerning is that PC is predicted to surpass colorectal cancer in 2040 and become the second-leading cause of cancer-related mortality, trailing only lung cancer [2]. To date, surgical resection remains the only treatment option with the potential to cure PC [3]. However, the vast majority of PC patients present with locally advanced disease or distant metastasis, and only a small percentage of individuals with PC can receive radical surgical treatment [4, 5]. Therefore, exploring the contributing factors and identifying individuals who are at a high risk of developing PC may help with early diagnosis and prevention of PC and its associated challenges.

Both genetic and lifestyle factors have been identified as key contributors to the development of PC. Several lifestyle factors, such as obesity, smoking and an unhealthy dietary pattern, have been positively associated with the risk of PC [6–9]. Given that lifestyle factors tend to coexist, researchers in several recent studies have reported associations between combinations of these factors and cancer risk, providing evidence to support the idea that an overall healthy lifestyle was associated with a reduction in cancer risk [10–12]. Individuals carrying pathogenic mutations in the PC susceptibility gene have a high risk of developing this type of cancer. Recent genome-wide association studies (GWASs) have been successful at identifying numerous single nucleotide polymorphisms (SNPs) associated with PC risk [13–16]. While individual variants may contribute only a limited amount to the heritable risk of PC, a polygenic risk score (PRS) has been shown to be effective in capturing the collective impact of multiple risk-associated variants. In a recent study performed on participants of the UK Biobank, it was reported that a higher PRS was associated with an increased risk of PC [17].

In previous studies, genetic and lifestyle factors were combined to estimate their association with cancers (e.g., breast cancer, lung cancer, thyroid cancer, colorectal cancer, and gastric cancer), and it was discovered that an overall healthy lifestyle might attenuate the risk of cancer due to genetic factors [18–22]. A study explored the relationship between lifestyle, genetic factors, and their combined effects on PC [23]. Additionally, a recent study reported the age-dependent association of these risk factors with PC [24]. However, few comprehensively investigated the age-dependent relationship between a healthy lifestyle and PC risk or the combined influence of lifestyle and genetic factors in this context. Hence, we conducted a prospective study using the UK Biobank to examine the beneficial association of a healthy lifestyle on PC in a different context. Then we comprehensively assessed the age-dependent association of a healthy lifestyle, genetic factors, and their combined effect with PC.

## Methods

### Study population

The UK Biobank was a large-scale prospective cohort study that involved recruiting approximately 500,000 participants (aged 37–73 years) from 2006 to 2010, containing in-depth genetic and health information for each participant. More information about the UK Biobank is described in detail elsewhere [25, 26].

**Fig. 1:**
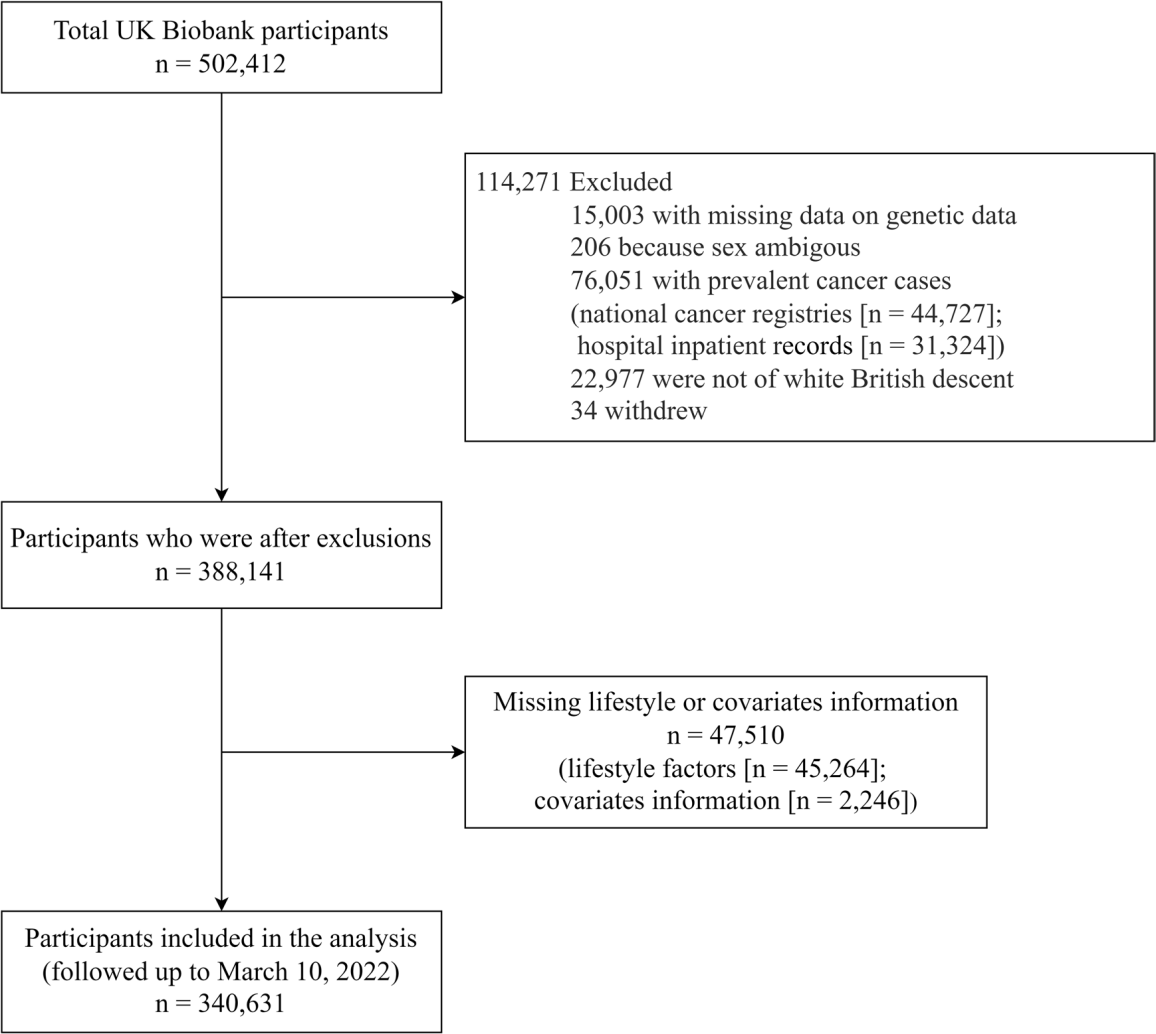
Flowchart for the selection of the analyzed study sample from the UK Biobank study

Our analysis was restricted to individuals of white British descent (including British, Irish, White, White and Asian, and White and Black African), as GWASs reported so far have been largely confined to this population. Out of a total of 502,412 participants, 161,781 were excluded from the cohort study, including 15,003 cases with missing genetic data, 206 cases with ambiguous sex information, 76,051 cases (national cancer registries [*n* = 44,727]; hospital inpatient [*n* = 31,324]) of prevalent cancer (diagnosed with cancer before enrollment), 22,977 individuals of nonwhite British ancestry, 34 withdrew, and 47,510 cases (lifestyle factors [*n* = 45,264]; covariate information [*n* = 2,246]) with missing lifestyle or covariate information (Additional file 1: Table S3). After excluding these factors, there were 340,631 participants in the study (Fig. 1).

The UK Biobank received ethics approval from the North West Multicenter Research Ethics Committee. All participants provided informed written consent, and they had the option to withdraw their personal data from the study at any time. The research was carried out utilizing resources from the UK Biobank, with application number 85224. This study adhered to the Strengthening the Reporting of Observational Studies in Epidemiology (STROBE) reporting guidelines (Additional file 2: Table S1).

### Outcomes

Prevalent cancer and incident PC cases within the UK Biobank cohort were identified by national cancer registries and hospital inpatient records. Diagnoses were recorded using the International Classification of Diseases-9 (ICD-9) and ICD-10 coding system (Additional file 1: Table S1). To prevent bias from analyzing heterogeneous molecular subtypes, patients diagnosed with neuroendocrine tumors were excluded. Death was ascertained via linkage to death registries. We computed the follow-up time from the date of attendance to the first diagnosis date, date of death, or last registered follow-up (10/03/2022), whichever occurred first.

### PRS calculation

We obtained genotype imputation data from the UK Biobank that are described in detail elsewhere [25, 26]. Briefly, blood samples were genotyped using either the Applied Biosystems UK BiLEVE Axiom Array by Affymetrix (807,411 markers) or the Applied Biosystems UK Biobank Axiom Array (825,927 markers). These arrays were highly compatible, as they shared 95% of the SNPs. These genotyping data were imputed using the UK 10 K and the 1000 Genomes Phase 3 reference panel and the Haplotype Reference Consortium (HRC) reference panel.

We applied three PRS models, namely PRS 54, PRS 22, and PRS 32, to assess the genetic risk of participants. Derived from a UK Biobank population study, PRS 54 was developed by Sharma, integrating SNPs from previous studies including Nakatochi (5 SNPs), Galeotti (30 SNPs), Molina (33 SNPs), Jia (22 SNPs), and 10 SNPs associated with PC in a large pan-cancer study [17, 27–31]. PRS 22 comprised 22 SNPs that were identified in previous GWAS studies conducted on populations of European descent (specifically, PanScan I-III and PanC4) [13, 16, 24]. Compared to PRS 22, PRS 32 included an additional 4 SNPs with suggestive associations and 6 SNPs that had not been replicated previously [13]. The SNP information for PRS 32 came from the GWAS Catalog (https://www.ebi.ac.uk/gwas/publications/29422604). Detailed information on the selected SNPs was available in Tables S4, S5 and S6 (Additional file 1: Tables S4, S5 and S6). Using a linkage disequilibrium (LD) clumping cutoff of r^2^ < 0.3 and conditional analyses, we applied three PRSs according to the SNP information in the above studies: PRS 54 constructed with 44 SNPs (10 SNPs with strong linkage disequilibrium were removed); PRS 22, comprising 22 SNPs; and PRS 32, consisting of 31 SNPs (1 SNP with strong linkage disequilibrium were removed). The effect size, P value, and risk allele for each SNP were sourced through the GWAS catalog and PubMed publications. Each SNP was recoded as 0, 1, or 2 according to the number of risk alleles. The weighted PRSs were generated using the PLINK “–score” command, which applies the following equation:

$$\mathrm{PRS} = \upbeta_1 \times \mathrm{SNP}_1 + \upbeta_2 \times \mathrm{SNP}_2 + \dots + \upbeta_n \times \mathrm{SNP}_n$$

Where n is the number of SNPs in the model and β is the per-allele log odds ratio (OR) for PC associated with SNP_n_. PRSs were standardized to a mean of zero and standard deviation (SD) of 1 through the computation of Z score (PRS-mean/SD). We defined genetic risk in thirds: "low" (the lowest third of the PRS), "intermediate" (the second third), and "high" (the highest third). We used the intermediate PRS to represent the normal people and as a reference for the entire study.

### Healthy lifestyle scores (HLSs)

We created HLSs primarily based on the World Cancer Research Fund/American Institute of Cancer Research (WCRF/AICR) lifestyle score and the American Cancer Society (ACS) Guidelines on Nutrition and Physical Activity for Cancer Prevention [32–34]. In this study, HLSs were generated using only five of the WCRF/AICR recommendations and one additional recommendation (smoking status) as the UK Biobank did not have a complete record of the cancer prevention recommendations of the WCRF/AICR on lifestyle factors. Nine lifestyle factors were used in constructing the HLSs: body mass index (BMI), waist circumference (WC), physical activity, sedentary time (time spent engaging in sedentary activity: driving, watching TV, computer using), fruit and vegetable intake, whole grain intake, red meat intake and processed meat intake, alcohol intake frequency, and smoking. Given the significant baseline characteristics of lifestyle differences between males and females, the association between different lifestyles and PC might be influenced by sex (Additional file 1: Table S8). Therefore, we created two HLSs: an unweighted HLS and a sex-specific weighted HLS (Additional file 1: Table S7 and S9).

Unweighted HLS was created as recommended by the WCRF/AICR and ACS guidelines (Additional file 1: Table S7). BMI, WC, physical activity, sedentary time, fruit and vegetable intake, whole grain intake, red meat and processed meat intake, alcohol intake frequency, and smoking were each assigned a score ranging from 0 to 0.5 (or 0 to 1), with the highest value of 0.5 (or 1) representing the healthiest behavior category. The unweighted HLS was then constructed by summing the scores for the nine lifestyle factors. The unweighted HLS ranged from 0 to 6 and was analyzed in this study according to 3 categories: unfavorable (≤ 2.75), intermediate (2.75, 3.75), and favorable (≥ 3.75), based on the tertile distribution of the unweighted HLS in all participants. To capture each lifestyle behavior at a more detailed spectrum, we created a sex-specific weighted healthy lifestyle score (Additional file 1: Table S9). The weighted HLS was derived based on β coefficients of each lifestyle factor in the Cox proportional hazards regression model (stratified by sex) with all 9 lifestyle factors and adjustment for age (continuous), education level, socioeconomic status, and the first 5 principal components of ancestry [35, 36]. The weighted HLS was analyzed in this study according to three categories: unfavorable (≥ -0.893), intermediate (-1.165, -0.893), and favorable (≤ -1.165) for males and unfavorable (≥ -1.107), intermediate (-1.355, -1.107), and favorable (≤ -1.107) for females, based on the tertile distribution of the weighted HLS in all participants. As a result, two HLSs (unweighted and weighted) were created.

### Covariate definition

The researchers used a touchscreen questionnaire and interview to collect information on other covariates, including age, sex, education level, and socioeconomic status (Additional file 1: Table S2). Education level was categorized as college or university, upper secondary, lower secondary, vocational, or other. We utilized the Townsend deprivation index, which analyzes information on social class, employment, car availability, and housing, to evaluate the socioeconomic status of participants in the UK Biobank. They were then categorized as low (highest quintile), middle (quintiles 2 to 4), or high (lowest quintile).

### Statistical analyses

To compare categorical features, we employed the chi-square test, while the t-test was used for normally distributed continuous variables and the Mann‒Whitney U test for nonnormally distributed continuous variables. Numbers and percentages were used to report categorical variables, means (SD) for normally distributed continuous variables, and medians (IQR) for nonnormally distributed continuous variables. The predictive performance of PRSs and HLSs was quantified using receiver operating characteristic curves (ROC) and the area under the curve (AUC) metric. Z tests were used to compare AUCs between different PRSs using a paired design.

Cox proportional hazards models were used to assess the HRs and 95% CIs of PC in relation to PRS and lifestyle factors and the interaction between lifestyle and PRS on the risk of PC. *P* value for trend was determined by using the categorical variables as continuous. The proportional hazards assumptions for the Cox model were verified using the Schoenfeld residuals method. Models were adjusted for age (continuous), sex, socioeconomic status, education level, and the first 5 principal components of ancestry. Stratified analyses of the PRS were performed (unfavorable lifestyle as reference). Absolute risk was calculated as the percentage of incident PC cases occurring in a given group. To test the additive interaction of risk factors, we used Cox proportional hazards regression models as previously described and estimated the relative excess risk due to interaction (RERI) and its 95% CI. Additionally, we calculated the multiplicative interaction by modeling the multiplicative term between PRS and lifestyle in the model. To assess the age-dependent association of lifestyle, PRS, and their combined effect with PC, we re-categorized participants based on their age at enrollment and follow-up duration into three age groups: ≤ 60, 61–70, and > 70 years, and recalculated the follow-up time for each age group. Furthermore, we divided the participants into two groups based on sex to assess whether the association between HLS, PRS, and PC is influenced by sex.

To test the robustness of our results, we conducted a series of sensitivity analyses as follows: (1) repeated the analyses in a sample excluding participants who were diagnosed with PC within the 2 years of follow-up and those who died within 2 years of baseline; and (2) assessed the competing risk analysis using the subdistribution method proposed by Fine and Gray (setting the cancer cases or deaths as the competing event).

All statistical analyses were conducted using R software version 4.2.1 and SPSS v 26.0. All P value < 0.05 (two-sided) was considered significant.

## Results

### Baseline characteristics of the study population

Of the 340,631 participants included in our study, there were 175,197 females and 165,434 males. The baseline characteristics of the included participants are presented in Table 1 and Table S10 (Additional file 1: Table S10). The median age was 57 (IQR, 50–63) years. During a median follow-up of 13.05 (IQR, 12.33–13.74) years (4,363,430 person-years), there were 1,129 cases of incident PC with an incidence rate of 25.87 per 100,000 person-years in the total population. Compared to participants with an unfavorable lifestyle, those with intermediate and favorable lifestyles tended to have higher levels of education and higher socioeconomic status (Table 1). Compared with participants without PC, those who developed incident PC were more likely to be older, male, excessive alcohol drinkers, smokers, physically inactive, and to have an unfavorable lifestyle, lower educational attainment and socioeconomic status (Additional file 1: Table S10). In addition, we observed significant differences in demographic characteristics and lifestyle factors between males and females (Additional file 1: Table S8). Compared to females, males had a more unfavorable BMI, longer sedentary time, lower vegetable and fruit intake, higher red and processed meat intake, greater alcohol consumption, and were more likely to smoke.

**Table 1:** Baseline characteristics of participants in the UK Biobank (*N* = 340,631)

	No. (%)					
Characteristics	Weighted healthy lifestyle score			Unweighted healthy lifestyle score		
	Unfavorable	Intermediate	Favorable	Unfavorable	Intermediate	Favorable
	(*N* = 113,431)	(*N* = 113,858)	(*N* = 113,342)	(*N* = 116,605)	(*N* = 102,246)	(*N* = 121,780)
PC						
No	112,905 (99.5)	113,497 (99.7)	113,100 (99.8)	116,096 (99.6)	101,907 (99.7)	121,499 (99.8)
Yes	526 (0.5)	361 (0.3)	242 (0.2)	509 (0.4)	339 (0.3)	281 (0.2)
Age, median (IQR), year	57 (50–63)	58 (50–63)	56 (49–62)	58 (50–63)	57 (50–63)	56 (49–62)
Sex						
Female	58,355 (51.4)	58,607 (51.5)	58,235 (51.3)	44,499 (38.2)	53,212 (52.0)	77,486 (63.6)
Male	55,076 (48.6)	55,251 (48.5)	55,107 (48.6)	72,106 (61.8)	49,034 (48.0)	44,294 (36.4)
Education level						
College or University	31,371 (27.7)	39,433 (34.6)	48,797 (43.1)	34,789 (29.8)	35,529 (34.7)	49,283 (40.5)
Upper secondary	13,128 (11.6)	13,940 (12.2)	14,080 (12.4)	13,998 (12.0)	12,243 (12.0)	14,907 (12.2)
Lower secondary	33,978 (30.0)	31,126 (27.3)	27,468 (24.2)	33,626 (28.8)	28,155 (27.5)	30,791 (25.3)
Vocational	8,615 (7.6)	7,496 (6.6)	6,187 (5.5)	9,252 (7.9)	6,623 (6.5)	6,423 (5.3)
Other	26,339 (23.2)	21,863 (19.2)	16,810 (14.8)	24,940 (21.4)	19,696 (19.3)	20,376 (16.7)
Socioeconomic status						
Low	28,469 (25.1)	20,473 (18.0)	19,187 (16.9)	26,114 (22.4)	19,494 (19.1)	22,521 (18.5)
Middle	65,294 (57.6)	69,446 (61.0)	69,548 (61.4)	68,529 (58.8)	61,707 (60.4)	74,052 (60.8)
High	19,668 (17.3)	23,939 (21.0)	24,607 (21.7)	21,962 (18.8)	21,045 (20.6)	25,207 (20.7)
PRS						
Low	37,640 (33.2)	38,161 (33.5)	37,724 (33.3)	38,792 (33.3)	34,148 (33.4)	40,585 (33.3)
Intermediate	37,798 (33.3)	37,958 (33.3)	37,815 (33.4)	38,773 (33.3)	34,203 (33.5)	40,595 (33.3)
High	37,993 (33.5)	37,739 (33.1)	37,803 (33.4)	39,040 (33.5)	33,895 (33.2)	40,600 (33.3)

*Abbreviations*: *PC* Pancreatic cancer, *PRS* Polygenic risk score

### PRSs and HLSs

We applied three PRSs to predict the risk of PC. Density plots of the resulting scores showed that for each PRS model, there was a clear shift in the PRS distribution toward higher scores in the PC cases compared with the PC-free controls (Additional file 1: Fig. S1). Furthermore, we applied ROC curves and AUC metrics to evaluate the power of the PRSs (Additional file 1: Fig. S1). The ROC curves for PRS 54 demonstrated the best performance, significantly outperforming PRS 22 (PRS 54: 61.6%; 95% CI, 60.0%–63.2%; PRS 22: 60.2%; 95% CI, 58.6%–61.8%;* P* = 0.02007). Although the difference from PRS 32 was not significant, a slight improvement in the AUC was still observed (PRS 54: 61.6%; 95% CI, 60.0%–63.2%; PRS 32: 60.7%; 95% CI, 59.1%–62.2%; *P* = 0.1142). Combining the performance results and estimate, PRS 54 (hereinafter referred to as PRS) was used in the subsequent assessment. In addition, we also applied two HLSs to assess the risk of PC. To evaluate the predictive power of these HLSs, we used ROC curves and AUC metrics (Additional file 1: Fig. S1). The weighted HLS demonstrated better performance (*P* = 0.00018), with an AUC of 60.5% (95% CI, 58.9%–62.1%), compared to the unweighted HLS which had an AUC of 58.1% (95% CI, 56.5%–59.7%). In order to more fully evaluate the relationship between lifestyle and PC, both HLSs were considered throughout the whole analysis.

### Association between genetic risk and PC

**Fig. 2:**
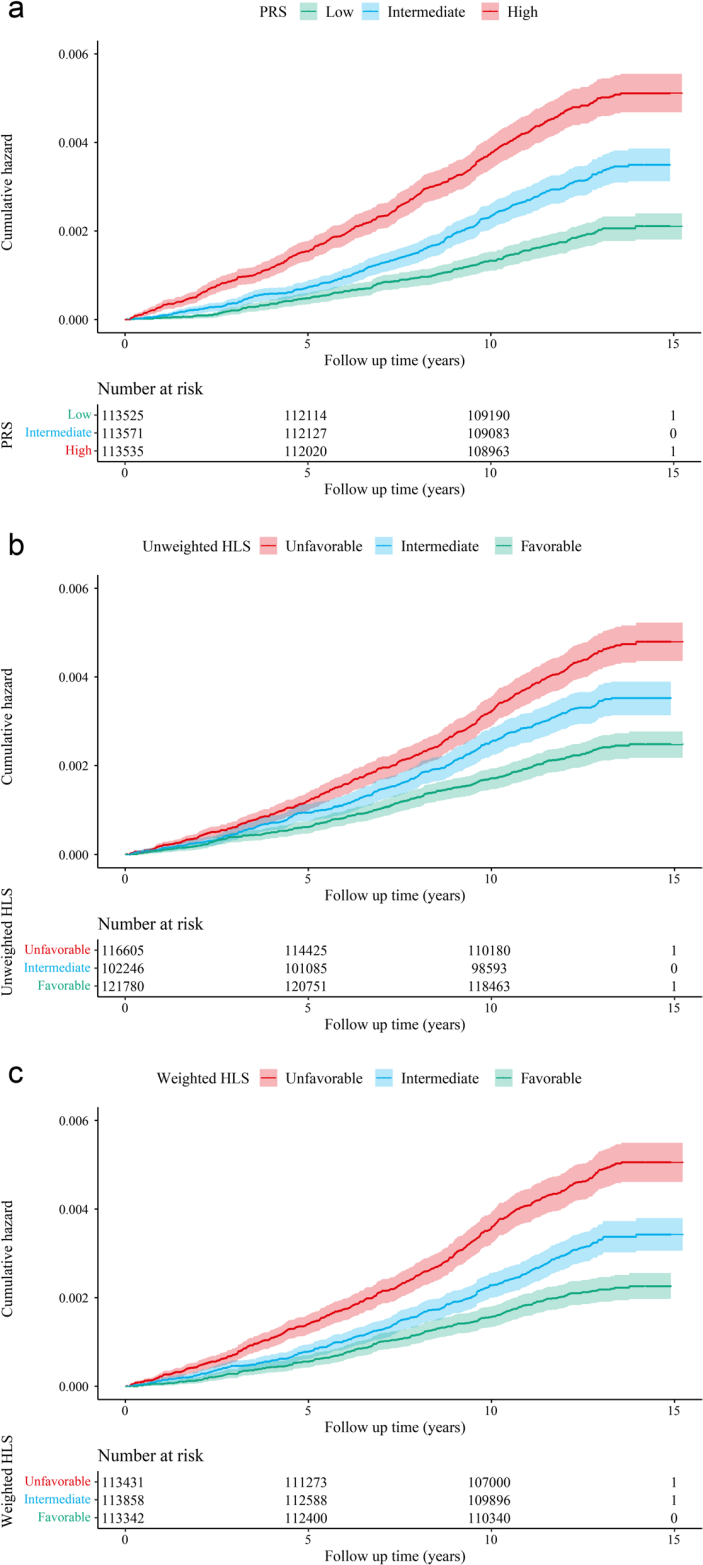
Cumulative risk of PC according to PRS and HLSs. Cumulative risk of PC during follow-up according to PRS (**a**), unweighted healthy lifestyle score (**b**), and weighted healthy lifestyle score (**c**)

We found that the PRS as a continuous variable was associated with an increased risk of PC (HR, 1.58; 95% CI, 1.47–1.71). In comparison to those with an intermediate PRS, participants with a low PRS had a 41% reduction in the risk of PC (95% CI, 0.50–0.70), while participants with a high PRS had a 1.51-fold increased risk of PC (95% CI, 1.32–1.73) (Table 2). Figure 2a shows the cumulative risk of PC in each genetic risk group during follow-up.

**Table 2:** Association between PRS, lifestyles, and risk of PC

Characteristics	PC/non-PC	Model 1^a^				Model 2^a^			
		HR (95% CI)	*P* value	HR (95% CI)	*P* value for trend ^c^	HR (95% CI)	*P* value	HR (95% CI)	*P* value for trend ^c^
PRS				1.58 (1.46–1.70)	1.45E-32			1.58 (1.47–1.71)	5.78E-33
Low	217/113,308	0.60 (0.50–0.70)	1.56E-09			0.59 (0.50–0.70)	1.23E-09		
Intermediate ^b^	364/113,207	1 [Reference]	NA			1 [Reference]	NA		
High	548/112,987	1.51 (1.32–1.72)	1.31E-09			1.51 (1.32–1.73)	9.96E-10		
Weighted healthy lifestyle score				0.67 (0.62–0.72)	6.65E-26			0.69 (0.64–0.74)	8.79E-22
Unfavorable	526/112,905	1 [Reference]	NA			1 [Reference]	NA		
Intermediate	361/113,497	0.67 (0.59–0.77)	6.86E-09			0.66 (0.58–0.76)	2.40E-09		
Favorable	242/113,100	0.45 (0.39–0.52)	9.36E-25			0.48 (0.41–0.56)	1.69E-20		
Unweighted healthy lifestyle score				0.72 (0.67–0.78)	4.30E-19			0.78 (0.72–0.84)	1.63E-11
Unfavorable	509/116,096	1 [Reference]	NA			1 [Reference]	NA		
Intermediate	339/101,907	0.75 (0.65–0.86)	3.60E-05			0.81 (0.70–0.93)	2.23E-03		
Favorable	281/121,499	0.52 (0.45–0.60)	8.57E-19			0.60 (0.52–0.70)	1.98E-11		

*Abbreviations*: *PC* Pancreatic cancer, *PRS* Polygenic risk score, *HR* Hazard ratio, *NA* Not applicable

^a^Model 1 was not adjusted; model 2 was adjusted for age (continuous), sex, education level, socioeconomic status, and the first 5 principal components of ancestry

^b^Set the intermediate value of PRS as the reference

^c^ *P* value for trend was determined by using the categorical variables as continuous

### Association between lifestyle and PC

As shown in Table 2, after adjustment for covariates in model 2, adherence to a healthier lifestyle was associated with a lower risk of PC (intermediate vs. unfavorable HR, 0.66; 95% CI, 0.58–0.76; favorable vs. unfavorable HR, 0.48; 95% CI, 0.41–0.56) in contrast to those with an unfavorable lifestyle. Similar results were observed for an unweighted HLS. Figure 2b and 2c show the cumulative risk of PC in the unweighted and weighted HLS groups during follow-up. In multi-adjusted Cox regression models, six of the nine lifestyle factors we examined were associated with a lower risk of PC. Specifically, favorable BMI, waist circumference, sedentary time, grain intake, moderate alcohol consumption, and not smoking were associated with a lower risk of PC (Additional file 1: Table S11).

### Combined association of genetic risk, lifestyle, and PC

**Fig. 3:**
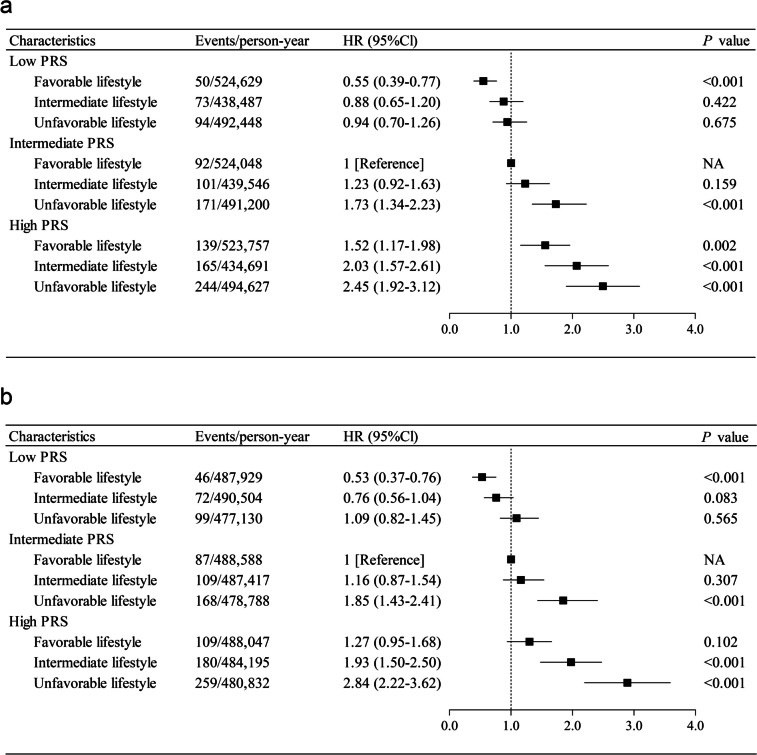
The combined effect of PRS and HLSs on the risk of incident PC. **a** The combined effect of unweighted healthy lifestyle score and PRS; **b** The combined effect of weighted healthy lifestyle score and PRS. The model was adjusted for age (continuous), sex, education level, socioeconomic status, and the first 5 principal components of ancestry

The combined analysis of the PRS and lifestyle and the risk of PC is presented in Fig. 3. In the combined analysis, compared to participants with a favorable lifestyle and an intermediate PRS, the HRs of PC were 1.16 (95% CI, 0.87–1.54) and 1.85 (95% CI, 1.43–2.41) in those with an intermediate and unfavorable lifestyle plus intermediate PRS and 1.93 (95% CI, 1.50–2.50) and 2.84 (95% CI, 2.22–3.62) for those with an intermediate and unfavorable lifestyle plus high PRS, respectively (Fig. 3b; Additional file 1: Table S12). Participants with a favorable lifestyle and low PRS had a 47% reduction in the risk of PC (95% CI, 0.37–0.76), compared to those with a favorable lifestyle and intermediate PRS. These results did not change significantly when using an unweighted HLS (Fig. 3a; Additional file 1: Table S13). Fig. S2 shows the cumulative risk of PC by the joint effect of lifestyle and PRS during follow-up. Furthermore, we analyzed the combined associations between genetic risk and healthy lifestyle components and the risk of PC (Additional file 1: Table S14).

### Interaction association of genetic risk, lifestyle, and PC

Table 3 displays the association between lifestyle and PC in the PRS-stratified analysis with unfavorable lifestyle as the reference. We observed that adopting a healthier lifestyle was associated with a lower risk of PC, regardless of genetic risk. In the low, intermediate, and high PRS groups, the HRs of favorable lifestyle were 0.49 (95% CI, 0.35–0.70), 0.55 (95% CI, 0.43–0.72), and 0.44 (95% CI, 0.35–0.55), respectively, compared with unfavorable lifestyle. Additionally, we assessed the absolute risk reduction for participants adhering to a healthy lifestyle across different genetic backgrounds over the full follow-up period (Additional file 1: Table S15). Compared to those who did not adhere to a healthy lifestyle, participants who adhered to a healthy lifestyle had an absolute risk reduction of 0.14% (95% CI, 0.08% to 0.20%), 0.21% (95% CI, 0.13%-0.30%), and 0.39% (95% CI, 0.29%-0.49%) in the low, intermediate, and high PRS categories, respectively. Similar results were shown with unweighted HLS.

**Table 3:** Associations of lifestyle components with incident PC according to PRS stratified analysis^a^

Characteristics	PRS						*P* value for interaction
	Low		Intermediate		High		
	HR (95% CI)	*P* value	HR (95% CI)	*P* value	HR (95% CI)	*P* value	
BMI (kg/m^2^)							0.827
Unfavorable	1 [Reference]	NA	1 [Reference]	NA	1 [Reference]	NA	
Intermediate	0.74 (0.54–1.01)	0.058	0.78 (0.61–1.01)	0.053	0.88 (0.72–1.07)	0.192	
Favorable	0.61 (0.42–0.87)	0.007	0.73 (0.55–0.96)	0.025	0.70 (0.56–0.89)	0.003	
Waist circumference (cm)							0.965
Unfavorable	1 [Reference]	NA	1 [Reference]	NA	1 [Reference]	NA	
Intermediate	0.88 (0.63–1.23)	0.454	0.81 (0.62–1.04)	0.102	0.78 (0.63–0.96)	0.018	
Favorable	0.78 (0.57–1.07)	0.126	0.74 (0.58–0.95)	0.018	0.66 (0.54–0.81)	< 0.001	
Physical activity 10 + min (days/week)							0.461
Unfavorable	1 [Reference]	NA	1 [Reference]	NA	1 [Reference]	NA	
Intermediate	0.95 (0.62–1.46)	0.805	0.96 (0.69–1.33)	0.788	0.88 (0.66–1.16)	0.351	
Favorable	0.78 (0.50–1.21)	0.268	0.80 (0.57–1.12)	0.198	0.94 (0.71–1.23)	0.643	
Sedentary time (hours/day)							0.153
Unfavorable	1 [Reference]	NA	1 [Reference]	NA	1 [Reference]	NA	
Intermediate	0.65 (0.48–0.88)	0.006	0.89 (0.71–1.12)	0.329	0.85 (0.71–1.02)	0.075	
Favorable	0.67 (0.43–1.05)	0.078	1.03 (0.76–1.41)	0.838	0.61 (0.46–0.82)	0.001	
Total fruit and vegetable intake							0.369
Unfavorable	1 [Reference]	NA	1 [Reference]	NA	1 [Reference]	NA	
Intermediate	1.04 (0.76–1.43)	0.809	0.75 (0.59–0.95)	0.019	0.97 (0.79–1.18)	0.750	
Favorable	0.97 (0.68–1.39)	0.867	0.79 (0.61–1.04)	0.088	0.90 (0.72–1.13)	0.354	
Whole grains intake							0.924
Unfavorable	1 [Reference]	NA	1 [Reference]	NA	1 [Reference]	NA	
Intermediate	0.75 (0.53–1.05)	0.095	0.90 (0.69–1.18)	0.433	0.89 (0.72–1.11)	0.317	
Favorable	0.75 (0.52–1.09)	0.133	0.83 (0.62–1.12)	0.222	0.85 (0.67–1.08)	0.186	
Red meat and processed meat intake							0.718
Unfavorable	1 [Reference]	NA	1 [Reference]	NA	1 [Reference]	NA	
Intermediate	0.88 (0.64–1.20)	0.409	1.04 (0.82–1.31)	0.752	1.10 (0.90–1.33)	0.349	
Favorable	0.84 (0.59–1.19)	0.323	0.80 (0.60–1.06)	0.120	0.93 (0.74–1.16)	0.512	
Alcohol intake frequency							0.457
Unfavorable	1 [Reference]	NA	1 [Reference]	NA	1 [Reference]	NA	
Intermediate	0.98 (0.71–1.37)	0.924	0.95 (0.74–1.22)	0.677	0.76 (0.62–0.93)	0.008	
Favorable	0.97 (0.66–1.42)	0.864	0.92 (0.69–1.24)	0.600	0.88 (0.69–1.11)	0.263	
Smoking							0.568
Unfavorable	1 [Reference]	NA	1 [Reference]	NA	1 [Reference]	NA	
Intermediate	0.49 (0.33–0.73)	< 0.001	0.66 (0.47–0.92)	0.013	0.53 (0.41–0.68)	< 0.001	
Favorable	0.45 (0.30–0.66)	< 0.001	0.62 (0.44–0.85)	0.004	0.42 (0.33–0.54)	< 0.001	
Unweighted healthy lifestyle score							0.645
Unfavorable	1 [Reference]	NA	1 [Reference]	NA	1 [Reference]	NA	
Intermediate	0.93 (0.68–1.26)	0.626	0.72 (0.56–0.92)	0.008	0.82 (0.68–1.01)	0.058	
Favorable	0.57 (0.40–0.81)	0.002	0.59 (0.46–0.77)	< 0.001	0.62 (0.50–0.76)	< 0.001	
Weighted healthy lifestyle score							0.679
Unfavorable	1 [Reference]	NA	1 [Reference]	NA	1 [Reference]	NA	
Intermediate	0.70 (0.52–0.95)	0.022	0.63 (0.50–0.81)	< 0.001	0.68 (0.56–0.82)	< 0.001	
Favorable	0.49 (0.35–0.70)	< 0.001	0.55 (0.43–0.72)	< 0.001	0.44 (0.35–0.55)	< 0.001	

*Abbreviations*: *PC* Pancreatic cancer, *PRS* Polygenic risk score, *BMI* Body mass index, *HR* Hazard ratio, *NA* not applicable

^a^Model was adjusted for age (continuous), sex, education level, socioeconomic status, and the first 5 principal components of ancestry

To further describe the relationship of genetic and lifestyle factors with the risk of PC, we conducted additional analyses on their multiplicative and additive interactions. While we did not observe a multiplicative interaction between lifestyles and the PRS, we observed a positive additive interaction for participants with a high PRS and an unfavorable weighted healthy lifestyle (RERI: 0.71; 95% CI, 0.21–1.22), compared to those with an intermediate PRS and favorable lifestyle. The additive association is presented in Table S21 (Additional file 1: Table S21), with a favorable lifestyle and a low PRS (or a favorable lifestyle and an intermediate PRS) as the reference. In our analysis, we identified positive additive interactions between PRS and various lifestyle components, specifically waist circumference, sedentary time, and smoking.

### The age-dependent association of genetic and lifestyle factors with PC

**Fig. 4:**
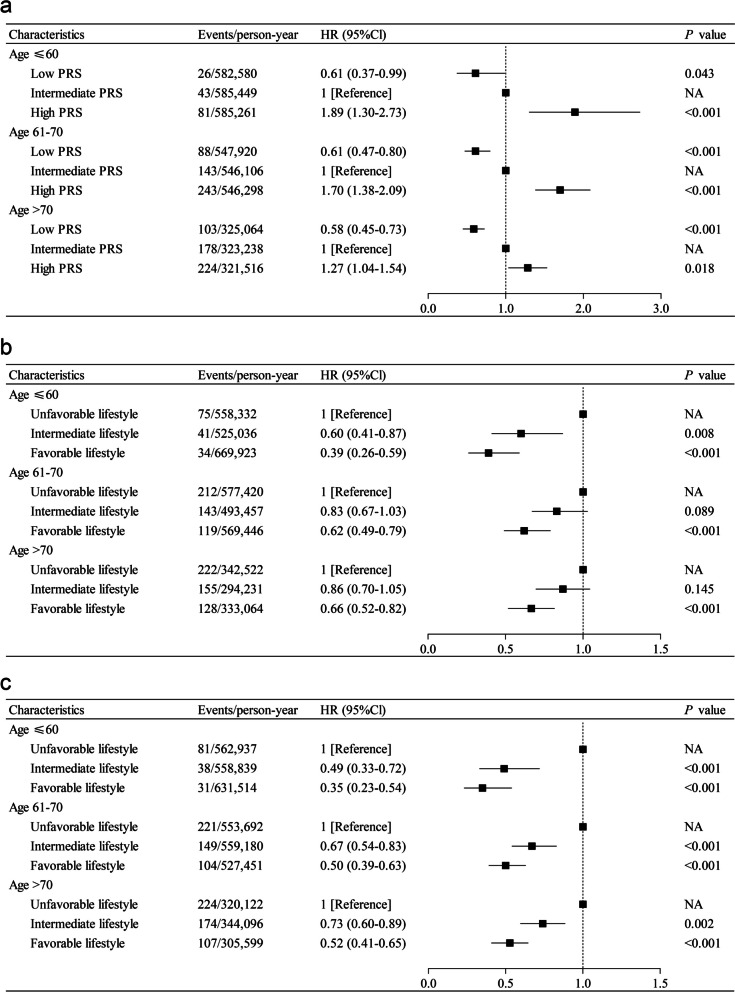
The effect of PRS and HLSs on the risk of incident PC, stratified by age groups. **a** PRS and PC according to age groups; **b** unweighted HLS and PC according to age groups; **c** weighted HLS and PC according to age groups. The model was adjusted for sex, education level, socioeconomic status, and the first 5 principal components of ancestry

To assess whether the association of lifestyle and genetic factors with PC risk was influenced by age, we further conducted stratified analyses by age groups. In the dataset re-categorized by age and follow-up duration, there were 150 cases aged ≤ 60 years (incidence rate, 8.56 per 100,000 person-years), 474 cases aged 61–70 years (incidence rate, 28.90 per 100,000 person-years), and 505 cases aged > 70 years (incidence rate, 52.07 per 100,000 person-years). Figure 4 displays the associations between HLSs and PRS with the risk of PC in different age subgroups. Among participants aged ≤ 60 years, the association between PRS and PC risk was most pronounced (HR, 1.89, 95% CI, 1.30–2.73), with lesser associations identified in older participants (Fig. 4a). In addition, we found that favorable weighted HLS were more strongly associated with a reduced risk of PC at younger ages, with lesser associations identified in older participants. Among participants with favorable versus unfavorable lifestyle, the HRs for PC were 0.35 (95% CI, 0.23–0.54) among those aged ≤ 60 years and 0.52 (95% CI, 0.41–0.65) among those aged > 70 years (Fig. 4c). Similar results were observed for unweighted HLS (Fig. 4b and Additional file 1: Table S16). When examining specific lifestyle factors, we found consistent results for BMI, waist circumference, sedentary time, red meat and processed meat intake, alcohol intake frequency, and smoking (Additional file 1: Table S17). We next examined whether the combined effects of HLSs and PRS would have differential associations with PC risk by age. The strongest association of the combined effects with PC risk was noted among participants aged ≤ 60 years (HR, 6.69; 95% CI, 2.99–14.93), with lesser associations identified in older participants (Additional file 1: Table S16). When comparing participants with a favorable lifestyle versus those with an unfavorable lifestyle, the absolute risk reduction was 0.07% (95% CI, 0.04%–0.10%) in those aged ≤ 60, 0.12% (95% CI, 0.08%–0.16%) in those aged 61–70, and 0.19% (95% CI, 0.13%–0.26%) in those aged > 70 (Additional file 1: Table S20). Furthermore, we used ROC curves to predict the PC among participants in different age groups (Additional file 1: Fig. S3). The PRS showed the highest AUC in participants aged ≤ 60 (AUC, 62.3%; 95% CI, 59.8%–64.9%), and both the unweighted HLS and weighted HLS had the highest AUC in participants aged ≤ 60 (unweighted HLS AUC, 60.2%; 95% CI, 57.6%–62.8%; weighted HLS AUC, 62.5%; 95% CI, 59.9%–65.1%).

To further understand the relationship between PC risk factors and age, we evaluated the interactions among genetic risk, lifestyle, and age (Additional file 1: Table S16 and Table S22). We observed a significant multiplicative interaction between age and the combined effect of weighted HLS and PRS on the risk of PC (*P* = 0.010). Furthermore, there were significant additive interactions involving age with the PRS, age with HLSs, and age with the combined effect of the HLSs and the PRS.

### Stratified analysis by sex

When stratified by sex, the association between PRS and PC risk was stronger in females (HR 1.55; 95% CI, 1.26–1.91). However, the protective association of weighted HLS against PC was slightly stronger for males (HR 0.46; 95% CI, 0.38–0.57). In the combined analysis, male participants with an unfavorable lifestyle and high PRS presented the highest risk of developing PC. In comparison to participants with a favorable lifestyle and an intermediate PRS, the HRs for PC were 2.95 (95% CI, 2.15–4.06) in males and 2.66 (95% CI, 1.82–3.89) in females, both having an unfavorable lifestyle and high PRS (Additional file 1: Table S18). The absolute risk reduction was 0.18% (95% CI, 0.12%–0.24%) for females and 0.33% (95% CI, 0.25%–0.40%) for males, when comparing participants with a favorable lifestyle to those with an unfavorable lifestyle (Additional file 1: Table S20). When we further analyzed the association between individual lifestyle components and PC, we found that aside from BMI, WC, sedentary time, and smoking which were all related to PC, alcohol was associated with PC in males, while grains intake showed a relationship in females (Additional file 1: Table S19). We further analyzed the interactions between lifestyle or genetic factors, or their combined effects, with sex. No significant multiplicative interactions were observed. However, positive additive interactions were observed between PRS and sex, weighted HLS and sex, as well as between the combined effect of weighted HLS and PRS with sex (Additional file 1: Table S23).

### Sensitivity analyses

Similar results were observed when we conducted the following assessments: (1) analyses excluding participants who died or developed incident PC within the first 2-year follow-up period (Additional file 1: Table S24 and Table S25); and (2) repeated analyses using a competing risk regression model (Additional file 1: Table S26 and Table S27).

## Discussion

In this large prospective study, we observed that both an unfavorable lifestyle and a high genetic risk were significantly associated with higher PC risk. Notably, participants with a high genetic risk and an unfavorable lifestyle had the highest risk of incident PC. In addition, regardless of the participants' age, sex, or genetic risk, maintaining a healthy lifestyle is associated with a lower risk of pancreatic cancer. When conducting stratified analysis based on age at enrollment and follow-up duration, we found that the association between genetic factors and PC was strongest among participants aged ≤ 60. Furthermore, we observed an age-dependent association of a healthy lifestyle with PC. In terms of absolute risk reduction, the protective relationship was more pronounced in the elderly population (age > 70). However, when considering hazard ratio, the protective association was stronger in the younger group (age ≤ 60), with weaker associations noted among the older participants. When considering the combined effect of HLS and PRS, we observed the highest risk among young people (age ≤ 60). The association between PRS and PC risk was stronger in females; however, the protective association of weighted HLS against PC was slightly stronger for males, and the combined effect was most strongly associated with PC risk in males. We observed several significant additive interactions among PRS, HLS, age, and sex. Moreover, there was a significant multiplicative interaction between age and the combined effect of weighted HLS and PRS on the risk of PC.

GWAS has become a powerful, hypothesis-free way to identify common alleles that influence disease risk. While the influence of a single SNP on the genetic susceptibility to PC may be limited, the PRS has demonstrated efficacy in predicting the hereditary predisposition to PC [29]. The findings of this study were consistent with previous studies indicating that individuals with a higher PRS were significantly more susceptible to PC. These genetic variants associated with PC have been validated by previous GWASs and PRSs [13–17, 24]. There is mounting evidence suggesting that a healthy lifestyle is linked to a decreased risk of PC [6, 23], a conclusion that is corroborated by our study. There were positive associations between unfavorable BMI, waist circumference, sedentary time, grain intake, smoking, and PC, which were also reported in the previous study [6, 7, 37–40]. In addition, we noted that smoking had the strongest association of all lifestyles with PC risk, which is in accordance with most earlier findings [38, 41, 42]. Smoking could act through several different mechanisms in the development of PC as smokers are exposed to a mixture of different carcinogenic and toxic compounds, both organic and inorganic, such as polycyclic aromatic hydrocarbons, heterocyclic aromatic amines, metals, and even radioactive gas [43]. These factors contribute to *KRAS* mutation, which is the most prevalent alteration in PC progression [44, 45]. Compared to excessive alcohol consumption, moderate alcohol consumption appeared to be a protective factor against PC. However, the protective effect of never drinking alcohol against PC was not statistically significant. The relationship between alcohol consumption and PC remains unclear and requires further research [6, 46, 47]. Furthermore, null significance was found between physical activity, fruit and vegetable intake, red meat and processed meat intake, and PC. However, the association between these lifestyle factors and PC continues to be a subject of debate [48–52].

A previous study explored the association between lifestyle, PRS, and PC risk, revealing that a healthy lifestyle is beneficially associated with PC, especially among those with higher genetic risk groups [23]. Additionally, a recent study reported the age-dependent association of these risk factors with PC [24]. However, few studies have comprehensively assessed the age-dependent relationship between lifestyle, PRS, and their combined effect on PC. In our study, we found that adhering to a healthy lifestyle was associated with a lower risk of PC, regardless of age. Moreover, an interesting phenomenon was observed, in terms of absolute risk reduction, the protective association was greater in the elderly population (age > 70). However, when considering hazard ratio, the protective association was more pronounced in the younger population (age ≤ 60), with lesser associations identified in older participants. This could be due to the fact that the initial absolute risk is potentially higher in the elderly, whereas the younger population has a lower initial absolute risk. In this study, it was observed that most cases of PC were diagnosed in older participants (aged > 60). When examining specific lifestyle factors, the results for BMI, waist circumference, sedentary time, intake of red and processed meat, frequency of alcohol intake, and smoking were consistent with previous findings [24]. Furthermore, we observed the strongest association between genetic risk and PC among participants aged ≤ 60, with the association gradually weakening in older groups. This is consistent with previous views suggesting that the connection between genetic factors and PC diminishes with age [24]. Further ROC analysis revealed that the predictive ability of PRS was highest among participants aged ≤ 60, and the predictive capability of HLSs was highest among participants aged ≤ 60. When considering the combined effect of HLS and PRS, we found that the highest risk was among young individuals (age ≤ 60). These findings have several important implications. Firstly, we evaluated both HR and ARR of lifestyle in relation to PC across different age groups, which helps us gain a more comprehensive understanding of the effects of lifestyle and allows for more precise risk assessment and decision-making. Secondly, to facilitate disease prevention, interventions targeting modifiable overall lifestyle might need to be implemented at younger ages. This is because, in terms of HR, the protective association of a healthy lifestyle appears to diminish as age increases. Lastly, future studies to investigate risk factors and prediction models for PC will need to consider age not only as a risk marker, but also as a stratification variable that may modify the association or predictive ability of other factors.

In this study, we observed a significant multiplicative interaction between age and the combined effect of weighted HLS and PRS on the risk of PC. This suggests that when a participant possesses both older age and an unhealthy lifestyle combined with a high genetic risk, their risk of developing PC is elevated to the highest level, exceeding the anticipated risk obtained from simply multiplying the effects of age with the combined effect of weighted HLS and PRS [53].In addition, we identified several significant additive interactions. These include the interaction of HLSs with PRS, age with PRS (or HLSs or the combined effect of HLSs and PRS), and sex with PRS (or weighted HLS or the combined effect of weighted HLS and PRS). The observed additive interactions suggest that when combined, the two risk factors for PC may produce a more pronounced effect on the likelihood of developing PC than the mere sum of their individual effects [54]. It's essential to assess interaction on the additive scale in studies within this field. This form of interaction provides an indication of the presence of biological interaction between risk factors and therefore has important etiological implications [54–56]. Two risk factors are said to have a biological interaction if both operate in the same pathway towards disease[56]. Furthermore, understanding additive interactions holds significant public health relevance, as it can pinpoint groups of individuals most likely to benefit from targeted interventions [55]. Specifically, in this study, older individuals, males, or those with a high genetic risk should pay more attention to maintaining a healthy lifestyle. Aging is inevitable, and both sex and genetic predisposition are innate and unchangeable. However, our lifestyle choices remain within our control and can be adjusted. The mechanisms underlying the interactions of these risk factors of PC are complex and not fully elucidated. The results need to be interpreted with caution, and further studies are needed.

### Strengths and limitations

The strengths of our present study are based on the UK Biobank dataset. The UK Biobank is a large sample prospective cohort study using standardized data collection protocols to reduce the risk of confounding bias. Additionally, we applied three PRSs and selected the one with the best performance. We also developed a sex-specific weighted HLS based on prior research. In subsequent analyses, the relationships of both HLSs with PC were taken into consideration. Furthermore, through additional stratified analyses, we were to explore the age-dependent association of lifestyle, PRS, and their combined effect with PC. To support our findings, we also performed a series of sensitivity analyses and competing risk model analyses.

There are several limitations in the current study. First, we were only able to measure lifestyle data for 502,412 individuals at baseline, so we could not assess longitudinal changes in lifestyle. Second, although we adjusted for known sources of bias, unmeasured confounding factors and reverse causation may still have influenced our findings. Third, our study was limited to individuals of European descent, so caution is needed when generalizing our findings to other populations. Fourth, the UK Biobank participants are not representative of the broader UK population since they tend to be health-conscious and well-educated. Finally, due to the limited number and accuracy of cancer site codes, we were unable to perform subgroup analyses on specific anatomic sites of PC.

## Conclusions

In this large prospective study, we discovered that both a high genetic predisposition and an unhealthy lifestyle were significantly associated with higher PC risk. Adhering to a healthy lifestyle was associated with a lower risk of PC, regardless of the participants' age, sex, or genetic risk. Importantly, our findings indicated the age-dependent association of lifestyle and genetic factors with PC. These results emphasize the pivotal importance of embracing a healthy lifestyle early on for effective prevention of PC, and could be invaluable in setting the optimal age to commence early preventive measures against PC.

### Supplementary Information

**Additional file 1: Table S1. **Data fields and International Classification of Disease Codes used for identification of PC and date of PC diagnosis in the UK Biobank cohort. **Table S2.** Data fields and information on variables in the UK Biobank cohort involved in this study. **Table S3.** The breakdown of the missing data for the lifestyle factors and other covariates. **Table S4.** Information on SNPs used to construct PRS 54 for pancreatic cancer. **Table S5.** Information on SNPs used to construct PRS 22 for pancreatic cancer. **Table S6.** Information on SNPs used to construct PRS 32 for pancreatic cancer. **Table S7.** Unweighted healthy lifestyle score components. **Table S8.** Baseline characteristics between female and male in the UK Biobank. **Table S9.** Sex-specific weighted healthy lifestyle score components. **Table S10.** Baseline characteristics of participants of PC in the UK Biobank. **Table S11.** Associations between lifestyle components and PC. **Table S12.** Combined association between PRS, weighted healthy lifestyle score, and PC. **Table S13.** Combined association between PRS, unweighted healthy lifestyle score, and PC. **Table S14.** Combined association between PRS, lifestyle components, and PC. **Table S15.** The absolute risk reductions of pancreatic cancer in different PRS. **Table S16.** Multivariable Cox regression analysis of genetic risk and lifestyles in relation to risk of PC, stratified by age. **Table S17.** Multivariable Cox regression analysis of lifestyle components in relation to risk of PC, stratified by age. **Table S18.** Multivariable Cox regression analysis of genetic risk and lifestyles in relation to risk of PC, stratified by sex. **Table S19.** Multivariable Cox regression analysis of lifestyle components in relation to risk of PC, stratified by sex. **Table S20.** The absolute risk reduction of pancreatic cancer in various age and sex groups. **Table S21.** The additive interaction between lifestyles and PRS (RERI). **Table S22.** The additive interaction between lifestyles, or PRS, and age (RERI). **Table S23.** The additive interaction between lifestyles, or PRS, and sex (RERI). **Table S24.** Associations between lifestyles, PRS, and PC after excluding the incidence of PC or death during the first 2 years of follow-up. **Table S25.** Combined analysis of PRS and lifestyles on the risk of PC in participants after excluding the incidence of PC or death during the first 2 years of follow-up. **Table S26.** Associations between lifestyles, PRS, and PC using competing risk analysis. **Table S27.** Combined analysis of PRS and lifestyle components on the risk of PC using competing risk analysis.** Fig. S1.** ROC curves and density plots of PRS and healthy lifestyle score. **Fig. S2.** Cumulative risk of PC by the joint effect of lifestyle and PRS. **Fig. S3.** ROC curves and AUC metrics of PRS and HLSs according to different age groups.

**Additional file 2: Table S1. **STROBE Statement—Checklist of items that should be included in reports of cohort studies.

## Data Availability

The data that support the findings of this study are available from UK Biobank but restrictions apply to the availability of these data, which were used under license for the current study, and so are not publicly available. Data are, however, available from the authors upon reasonable request and with permission of UK Biobank. More details can be found at https://www.ukbiobank.ac.uk/.

## Abbreviations

PC: Pancreatic cancer
PRS: Polygenic risk score
HLS: Healthy lifestyle score
WCRF/AICR: World Cancer Research Fund/American Institute of Cancer Research
ACS: American Cancer Society
HR: Hazard ratio
CI: Confidence interval
GWAS: Genome-wide association study
SNPs: Single nucleotide polymorphisms
ICD-9: International Classification of Diseases-9
ICD-10: International Classification of Diseases-10
HRC: Haplotype Reference Consortium
LD: Linkage disequilibrium
OR: Odds ratio
SD: Standard deviation
BMI: Body mass index
WC: Waist circumference
IQR: Interquartile range
RERI: Relative excess risk due to interaction
ROC: Receiver operating characteristic curve
AUC: Area under the curve
ARR: Absolute risk reduction

## References

[1] Halbrook CJ, Lyssiotis CA (2023). Pasca di Magliano M, Maitra A. Pancreatic Cancer: Advances Challenges Cell.

[2] Rahib L, Wehner MR, Matrisian LM, Nead KT (2021). Estimated projection of us cancer incidence and death to 2040. JAMA Netw Open.

[3] Mizrahi JD, Surana R, Valle JW, Shroff RT (2020). Pancreatic cancer. Lancet.

[4] Bengtsson A, Andersson R, Ansari D (2020). The actual 5-year survivors of pancreatic ductal adenocarcinoma based on real-world data. Sci Rep.

[5] Ryan DP, Hong TS, Bardeesy N (2014). Pancreatic adenocarcinoma. N Engl J Med.

[6] Zanini S, Renzi S, Limongi AR, Bellavite P, Giovinazzo F, Bermano G (2021). A review of lifestyle and environment risk factors for pancreatic cancer. Eur J Cancer.

[7] Klein AP (2021). Pancreatic cancer epidemiology: Understanding the role of lifestyle and inherited risk factors. Nat Rev Gastroenterol Hepatol.

[8] Huang J, Lok V, Ngai CH, Zhang L, Yuan J, Lao XQ (2021). Worldwide burden of, risk factors for, and trends in pancreatic cancer. Gastroenterology.

[9] Naudin S, Viallon V, Hashim D, Freisling H, Jenab M, Weiderpass E (2020). Healthy lifestyle and the risk of pancreatic cancer in the epic study. Eur J Epidemiol.

[10] Catsburg C, Miller AB, Rohan TE (2014). Adherence to cancer prevention guidelines and risk of breast cancer. Int J Cancer.

[11] Turati F, Bravi F, Di Maso M, Bosetti C, Polesel J, Serraino D (2017). Adherence to the world cancer research fund/american institute for cancer research recommendations and colorectal cancer risk. Eur J Cancer.

[12] Zhang Z-Q, Li Q-J, Hao F-B, Wu Y-Q-L, Liu S, Zhong G-C. Adherence to the 2018 world cancer research fund/american institute for cancer research cancer prevention recommendations and pancreatic cancer incidence and mortality: A prospective cohort study. Cancer Med. 2020;9(18):6843–53. 10.1002/cam4.3348. 10.1002/cam4.3348 PMC7520356 32716590

[13] Klein AP, Wolpin BM, Risch HA, Stolzenberg-Solomon RZ, Mocci E, Zhang M (2018). Genome-wide meta-analysis identifies five new susceptibility loci for pancreatic cancer. Nat Commun.

[14] Petersen GM, Amundadottir L, Fuchs CS, Kraft P, Stolzenberg-Solomon RZ, Jacobs KB, et al. A genome-wide association study identifies pancreatic cancer susceptibility loci on chromosomes 13q22.1, 1q32.1 and 5p15.33. Nat Genet. 2010;42(3):224–8. 10.1038/ng.522. 10.1038/ng.522 PMC2853179 20101243

[15] Amundadottir L, Kraft P, Stolzenberg-Solomon RZ, Fuchs CS, Petersen GM, Arslan AA (2009). Genome-wide association study identifies variants in the abo locus associated with susceptibility to pancreatic cancer. Nat Genet.

[16] Wolpin BM, Rizzato C, Kraft P, Kooperberg C, Petersen GM, Wang Z, et al. Genome-wide association study identifies multiple susceptibility loci for pancreatic cancer. Nat Genet. 2014;46(9). 10.1038/ng.3052. 10.1038/ng.3052 PMC4191666 25086665

[17] Sharma S, Tapper WJ, Collins A, Hamady ZZR. Predicting pancreatic cancer in the uk biobank cohort using polygenic risk scores and diabetes mellitus. Gastroenterology. 2022;162(6). 10.1053/j.gastro.2022.01.016. 10.1053/j.gastro.2022.01.016 35065983

[18] Arthur RS, Wang T, Xue X, Kamensky V, Rohan TE (2020). Genetic factors, adherence to healthy lifestyle behavior, and risk of invasive breast cancer among women in the uk biobank. J Natl Cancer Inst.

[19] Choi J, Jia G, Wen W, Shu X-O, Zheng W (2021). Healthy lifestyles, genetic modifiers, and colorectal cancer risk: A prospective cohort study in the uk biobank. Am J Clin Nutr.

[20] Liang H, Zhou X, Zhu Y, Li D, Jing D, Su X (2023). Association of outdoor air pollution, lifestyle, genetic factors with the risk of lung cancer: A prospective cohort study. Environ Res.

[21] Jin G, Lv J, Yang M, Wang M, Zhu M, Wang T (2020). Genetic risk, incident gastric cancer, and healthy lifestyle: A meta-analysis of genome-wide association studies and prospective cohort study. Lancet Oncol.

[22] Zhu M, Wang T, Huang Y, Zhao X, Ding Y, Zhu M (2021). Genetic risk for overall cancer and the benefit of adherence to a healthy lifestyle. Cancer Res.

[23] Byrne S, Boyle T, Ahmed M, Lee SH, Benyamin B, Hyppönen E (2023). Lifestyle, genetic risk and incidence of cancer: A prospective cohort study of 13 cancer types. Int J Epidemiol.

[24] Yuan C, Kim J, Wang QL, Lee AA, Babic A, Amundadottir LT (2022). The age-dependent association of risk factors with pancreatic cancer. Ann Oncol.

[25] Sudlow C, Gallacher J, Allen N, Beral V, Burton P, Danesh J (2015). Uk biobank: An open access resource for identifying the causes of a wide range of complex diseases of middle and old age. PLoS Med.

[26] Bycroft C, Freeman C, Petkova D, Band G, Elliott LT, Sharp K (2018). The uk biobank resource with deep phenotyping and genomic data. Nature.

[27] Rashkin SR, Graff RE, Kachuri L, Thai KK, Alexeeff SE, Blatchins MA (2020). Pan-cancer study detects genetic risk variants and shared genetic basis in two large cohorts. Nat Commun.

[28] Jia G, Lu Y, Wen W, Long J, Liu Y, Tao R, et al. Evaluating the utility of polygenic risk scores in identifying high-risk individuals for eight common cancers. JNCI Cancer Spectr. 2020;4(3):pkaa021. 10.1093/jncics/pkaa021. 10.1093/jncics/pkaa021 PMC7306192 32596635

[29] Nakatochi M, Lin Y, Ito H, Hara K, Kinoshita F, Kobayashi Y (2018). Prediction model for pancreatic cancer risk in the general japanese population. PLoS ONE.

[30] Galeotti AA, Gentiluomo M, Rizzato C, Obazee O, Neoptolemos JP, Pasquali C (2021). Polygenic and multifactorial scores for pancreatic ductal adenocarcinoma risk prediction. J Med Genet.

[31] Molina-Montes E, Coscia C, Gómez-Rubio P, Fernández A, Boenink R, Rava M (2021). Deciphering the complex interplay between pancreatic cancer, diabetes mellitus subtypes and obesity/bmi through causal inference and mediation analyses. Gut.

[32] Shams-White MM, Brockton NT, Mitrou P, Romaguera D, Brown S, Bender A, et al. Operationalizing the 2018 world cancer research fund/american institute for cancer research (wcrf/aicr) cancer prevention recommendations: A standardized scoring system. Nutrients. 2019;11(7). doi: 10.3390/nu11071572. 10.3390/nu11071572 PMC6682977 31336836

[33] Rock CL, Thomson CA, Sullivan KR, Howe CL, Kushi LH, Caan BJ (2022). American cancer society nutrition and physical activity guideline for cancer survivors. CA Cancer J Clin.

[34] Hill EB, Grainger EM, Young GS, Clinton SK, Spees CK. Application of the updated wcrf/aicr cancer prevention score as an outcome for cancer survivors participating in a tailored and intensive dietary and physical activity intervention. Nutrients. 2022;14(22). 10.3390/nu14224751. 10.3390/nu14224751 PMC9699073 36432442

[35] Lourida I, Hannon E, Littlejohns TJ, Langa KM, Hyppönen E, Kuzma E (2019). Association of lifestyle and genetic risk with incidence of dementia. JAMA.

[36] Feng X, Wang F, Yang W, Zheng Y, Liu C, Huang L (2022). Association between genetic risk, adherence to healthy lifestyle behavior, and thyroid cancer risk. JAMA Netw Open.

[37] Li D, Morris JS, Liu J, Hassan MM, Day RS, Bondy ML (2009). Body mass index and risk, age of onset, and survival in patients with pancreatic cancer. JAMA.

[38] Jiao L, Mitrou PN, Reedy J, Graubard BI, Hollenbeck AR, Schatzkin A (2009). A combined healthy lifestyle score and risk of pancreatic cancer in a large cohort study. Arch Intern Med.

[39] Sung H, Siegel RL, Torre LA, Pearson-Stuttard J, Islami F, Fedewa SA, et al. Global patterns in excess body weight and the associated cancer burden. CA Cancer J Clin. 2019;69(2). 10.3322/caac.21499. 10.3322/caac.21499 30548482

[40] Lauby-Secretan B, Scoccianti C, Loomis D, Grosse Y, Bianchini F, Straif K (2016). Body fatness and cancer–viewpoint of the iarc working group. N Engl J Med.

[41] Lugo A, Peveri G, Bosetti C, Bagnardi V, Crippa A, Orsini N (2018). Strong excess risk of pancreatic cancer for low frequency and duration of cigarette smoking: A comprehensive review and meta-analysis. Eur J Cancer.

[42] Gandini S, Botteri E, Iodice S, Boniol M, Lowenfels AB, Maisonneuve P (2008). Tobacco smoking and cancer: A meta-analysis. Int J Cancer.

[43] Duell EJ (2012). Epidemiology and potential mechanisms of tobacco smoking and heavy alcohol consumption in pancreatic cancer. Mol Carcinog.

[44] Rivenson A, Hoffmann D, Prokopczyk B, Amin S, Hecht SS (1988). Induction of lung and exocrine pancreas tumors in f344 rats by tobacco-specific and areca-derived n-nitrosamines. Cancer Res.

[45] Schuller HM (2002). Mechanisms of smoking-related lung and pancreatic adenocarcinoma development. Nat Rev Cancer.

[46] Lucenteforte E, La Vecchia C, Silverman D, Petersen GM, Bracci PM, Ji BT (2012). Alcohol consumption and pancreatic cancer: A pooled analysis in the international pancreatic cancer case-control consortium (panc4). Ann Oncol.

[47] Rahman F, Cotterchio M, Cleary SP, Gallinger S (2015). Association between alcohol consumption and pancreatic cancer risk: A case-control study. PLoS ONE.

[48] Rohrmann S, Linseisen J, Nöthlings U, Overvad K, Egeberg R, Tjønneland A (2013). Meat and fish consumption and risk of pancreatic cancer: Results from the european prospective investigation into cancer and nutrition. Int J Cancer.

[49] Behrens G, Jochem C, Schmid D, Keimling M, Ricci C, Leitzmann MF (2015). Physical activity and risk of pancreatic cancer: A systematic review and meta-analysis. Eur J Epidemiol.

[50] Bao Y, Michaud DS (2008). Physical activity and pancreatic cancer risk: A systematic review. Cancer Epidemiol Biomarkers Prev.

[51] Larsson SC, Hakanson N, Permert J, Wolk A (2006). Meat, fish, poultry and egg consumption in relation to risk of pancreatic cancer: A prospective study. Int J Cancer.

[52] Larsson SC, Hakansson N, Naslund I, Bergkvist L, Wolk A (2006). Fruit and vegetable consumption in relation to pancreatic cancer risk: A prospective study. Cancer Epidemiol Biomarkers Prev.

[53] de Mutsert R, Jager KJ, Zoccali C, Dekker FW (2009). The effect of joint exposures: Examining the presence of interaction. Kidney Int.

[54] Knol MJ, VanderWeele TJ, Groenwold RH, Klungel OH, Rovers MM, Grobbee DE (2011). Estimating measures of interaction on an additive scale for preventive exposures. Eur J Epidemiol.

[55] VanderWeele TJ, Knol MJ (2014). A tutorial on interaction. Epidemiologic Methods.

[56] Andersson T, Alfredsson L, Källberg H, Zdravkovic S, Ahlbom A (2005). Calculating measures of biological interaction. Eur J Epidemiol.

