# Supplementary material for: Association of genetic risk and lifestyle with pancreatic cancer and their age dependency: a large prospective cohort study in the UK Biobank
Source: BMC Med. 2023 Dec 8;21:489. doi: 10.1186/s12916-023-03202-0 (PMC10709905; doi:10.1186/s12916-023-03202-0)
Supplement: Supplementary file 1 — Additional file 1: Table S1. Data fields and International Classification of Disease Codes used for identification of PC and date of PC diagnosis in the UK Biobank cohort. Table S2. Data fields and information on variables in the UK Biobank cohort involved in this study. Table S3. The breakdown of the missing data for the lifestyle factors and other covariates. Table S4. Information on SNPs used to construct PRS 54 for pancreatic cancer. Table S5. Information on SNPs used to construct PRS 22 for pancreatic cancer. Table S6. Information on SNPs used to construct PRS 32 for pancreatic cancer. Table S7. Unweighted healthy lifestyle score components. Table S8. Baseline characteristics between female and male in the UK Biobank. Table S9. Sex-specific weighted healthy lifestyle score components. Table S10. Baseline characteristics of participants of PC in the UK Biobank. Table S11. Associations between lifestyle components and PC. Table S12. Combined association between PRS, weighted healthy lifestyle score, and PC. Table S13. Combined association between PRS, unweighted healthy lifestyle score, and PC. Table S14. Combined association between PRS, lifestyle components, and PC. Table S15. The absolute risk reductions of pancreatic cancer in different PRS. Table S16. Multivariable Cox regression analysis of genetic risk and lifestyles in relation to risk of PC, stratified by age. Table S17. Multivariable Cox regression analysis of lifestyle components in relation to risk of PC, stratified by age. Table S18. Multivariable Cox regression analysis of genetic risk and lifestyles in relation to risk of PC, stratified by sex. Table S19. Multivariable Cox regression analysis of lifestyle components in relation to risk of PC, stratified by sex. Table S20. The absolute risk reduction of pancreatic cancer in various age and sex groups. Table S21. The additive interaction between lifestyles and PRS (RERI). Table S22. The additive interaction between lifestyles, or PRS, and age (RERI). [file 12916_2023_3202_MOESM1_ESM.docx]

**Additional File 1**

**Table S1.** Data fields and International Classification of Disease Codes used for identification of PC and date of PC diagnosis in the UK Biobank cohort

**Table S2.** Data fields and information on variables in the UK Biobank cohort involved in this study

**Table S3.** The breakdown of the missing data for the lifestyle factors and other covariates

**Table S4.** Information on SNPs used to construct PRS 54 for pancreatic cancer

**Table S5.** Information on SNPs used to construct PRS 22 for pancreatic cancer

**Table S6.** Information on SNPs used to construct PRS 32 for pancreatic cancer

**Table S7.** Unweighted healthy lifestyle score components

**Table S8.** Baseline characteristics between female and male in the UK Biobank

**Table S9.** Sex-specific weighted healthy lifestyle score components

**Table S10.** Baseline characteristics of participants of PC in the UK Biobank

**Table S11.** Associations between lifestyle components and PC

**Table S12.** Combined association between PRS, weighted healthy lifestyle score, and PC

**Table S13.** Combined association between PRS, unweighted healthy lifestyle score, and PC

**Table S14.** Combined association between PRS, lifestyle components, and PC

**Table S15.** The absolute risk reductions of pancreatic cancer in different PRS

**Table S16.** Multivariable Cox regression analysis of genetic risk and lifestyles in relation to risk of PC, stratified by age

**Table S17.** Multivariable Cox regression analysis of lifestyle components in relation to risk of PC, stratified by age

**Table S18.** Multivariable Cox regression analysis of genetic risk and lifestyles in relation to risk of PC, stratified by sex

**Table S19.** Multivariable Cox regression analysis of lifestyle components in relation to risk of PC, stratified by sex

**Table S20.** The absolute risk reduction of pancreatic cancer in various age and sex groups

**Table S21.** The additive interaction between lifestyles and PRS (RERI)

**Table S22.** The additive interaction between lifestyles, or PRS, and age (RERI)

**Table S23.** The additive interaction between lifestyles, or PRS, and sex (RERI)

**Table S24.** Associations between lifestyles, PRS, and PC after excluding the incidence of PC or death during the first 2 years of follow-up

**Table S25.** Combined analysis of PRS and lifestyles on the risk of PC in participants after excluding the incidence of PC or death during the first 2 years of follow-up

**Table S26.** Associations between lifestyles, PRS, and PC using competing risk analysis

**Table S27.** Combined analysis of PRS and lifestyle components on the risk of PC using competing risk analysis

**Fig. S1.** ROC curves and density plots of PRS and healthy lifestyle score

**Fig. S2.** Cumulative risk of PC by the joint effect of lifestyle and PRS

**Fig. S3.** ROC curves and AUC metrics of PRS and HLSs according to different age groups

| **Table S1. Data fields and International Classification of Disease Codes used for identification of PC and date of PC diagnosis in the UK Biobank cohort** | | |  |
| --- | --- | --- | --- |
| **Definition** | **Data field** | **Code** |  |
| Pancreatic cancer | ICD 10 codes for: 40006 National cancer registry 41270 Hospital inpatient | C25.0 Head of pancreas C25.1 Body of pancreas C25.2 Tail of pancreas C25.3 Pancreatic duct C25.7 Other parts of pancreas C25.8 Overlapping lesion of pancreas C25.9 Pancreas, unspecified |  |
|  |  |  |  |
|  |  |  |  |
|  |  |  |  |
|  |  |  |  |
|  |  |  |  |
|  |  |  |  |
|  | ICD 9 codes for: 40013 National cancer registry 41271 Hospital inpatient | 1570 Malignant neoplasm of head of pancreas 1571 Malignant neoplasm of body of pancreas 1572 Malignant neoplasm of tail of pancreas 1573 Malignant neoplasm of pancreatic duct 1578 Malignant neoplasm of pancreas, other 1579 Malignant neoplasm of pancreas, part unspecified |  |
|  |  |  |  |
|  |  |  |  |
|  |  |  |  |
|  |  |  |  |
|  |  |  |  |
| Date of cancer diagnosis (national cancer registry) | 40005 |  |  |
| Date of first in-patient diagnosis - ICD10 (hospital inpatient) | 41280 |  |  |
| Date of first in-patient diagnosis - ICD9 (hospital inpatient) | 41281 |  |  |
| Prevalent cancer within the UK Biobank cohort was identified through national cancer registries (data fields: 40006, 40013) and hospital inpatient records (data fields: 41270, 41271). | | |  |
| Abbreviations: PC, pancreatic cancer; ICD, International Classification of Diseases. | | |  |

| **Table S2. Data fields and information on variables in the UK Biobank cohort involved in this study** | | |
| --- | --- | --- |
| **Category** | **Data field** | **Description** |
| **Demographic factors** |  |  |
| Age | 21022 | Age at recruitment |
|  | 53 | Date of attending assessment center |
| Date of death | 40000 | Date of death |
| Sex | 31 | Sex |
| Education level | 6138 | Qualifications |
| Socioeconomic status | 189 | Townsend deprivation index at recruitment |
| Ethnic background | 21000 | Ethnic background |
| **Lifestyle factors** |  |  |
| BMI | 21001 | Body mass index (BMI) |
| Waist circumference (cm) | 48 | Waist circumference |
| Physical activity 10+ min (day/week) | 884 | Number of days/week of moderate physical activity 10+ minutes |
|  | 904 | Number of days/week of vigorous physical activity 10+ minutes |
| Sedentary time (hours/day) | 1090 | Time spent driving |
|  | 1080 | Time spent using computer |
|  | 1070 | Time spent watching television (TV) |
| Fruit and vegetable intake | 1309 | Fresh fruit intake |
|  | 1319 | Dried fruit intake |
|  | 1289 | Cooked vegetable intake |
|  | 1299 | Salad / raw vegetable intake |
| Whole grains intake | 1458 | Cereal intake |
|  | 1438 | Bread intake |
| Red meat and processed meat intake | 1369 | Beef intake |
|  | 1379 | Lamb/mutton intake |
|  | 1389 | Pork intake |
|  | 1349 | Processed meat intake |
| Alcohol intake frequency | 1558 | Alcohol intake frequency |
| Smoking | 20116 | Smoking status |

| **Table S3. The breakdown of the missing data for the lifestyle factors and other covariates** | |
| --- | --- |
| Characteristic | Missing information (%) |
| Lifestyle factors | 45264 (11.66) |
| BMI | 1237 (0.32) |
| Waist circumference | 61 (0.02) |
| Physical activity 10+ min | 28729 (7.40) |
| Sedentary time | 5728 (1.48) |
| Fruit and vegetable intake | 5216 (1.34) |
| Whole grains intake | 1796 (0.46) |
| Red meat and processed meat intake | 1606 (0.41) |
| Alcohol intake frequency | 104 (0.03) |
| Smoking | 787 (0.20) |
| Covariates | 2246 (0.58) |
| Age | 0 (0) |
| Sex | 0 (0) |
| Education level | 1851 (0.48) |
| Socioeconomic status | 395 (0.10) |

| **Table S4. Information on SNPs used to construct PRS 54 for pancreatic cancer** | | | | | | | | | | |
| --- | --- | --- | --- | --- | --- | --- | --- | --- | --- | --- |
| SNP | Chr | Position ^a^ | Gene | Risk allele | Other allele | MAF ^b^ | OR | Beta | P value | LD exclusion |
| rs10094872 | 8 | 128719884 | CASC11 | T | A | T=0.366278 | 1.15 | 0.1398 | 3.63E-03 |  |
| rs10919791 | 1 | 199965168 | RNU6-716P, AL445687.2 | G | A | A=0.226206 | 1.26 | 0.2311 | 1.16E-04 |  |
| rs10991043 | 9 | 106797388 | AL590381.1, SMC2-AS1 | C | T | C=0.376445 | 1.01 | 0.0100 | 8.65E-01 | LD with rs2417487 |
| rs11655237 | 17 | 70400166 | LINC00511 | T | C | T=0.124143 | 1.15 | 0.1398 | 4.62E-02 | LD with rs7214041 |
| rs1182933 | 12 | 121454622 | OASL, C12orf43 | T | C | T=0.304076 | 1.01 | 0.0100 | 8.75E-01 |  |
| rs12478462 | 2 | 153654720 | ARL6IP6, AC009969.1 | G | T | G=0.246948 | 1.07 | 0.0677 | 2.08E-01 |  |
| rs12615966 | 2 | 105378957 | LINC01114, HMGB3P11 | T | C | T=0.137942 | 0.98 | -0.0202 | 7.58E-01 |  |
| rs13303010 | 1 | 894573 | NOC2L | G | A | G=0.136619 | 1.30 | 0.2624 | 4.67E-04 |  |
| rs138585571 | 3 | 98188580 | OR5K1 | T | C | T=0.033879 | 1.50 | 0.4055 | 7.00E-05 |  |
| rs144239147 | 17 | 48154668 | ITGA3 | A | G | A=0.014068 | 1.25 | 0.2231 | 2.00E-01 |  |
| rs145695688 | 11 | 65039350 | POLA2, AP000944.5 | T | C | T=0.007835 | 1.02 | 0.0198 | 9.23E-01 |  |
| rs148512905 | 3 | 18984747 | SATB1-AS1, RNU6-138P | C | A | C=0.001717 | 1.88 | 0.6313 | 1.99E-03 |  |
| rs1486134 | 2 | 67639769 | LINC02831, ETAA1 | G | T | G=0.278874 | 1.15 | 0.1398 | 5.51E-03 | LD with rs962856 |
| rs1517037 | 18 | 56878274 | SEC11C, GRP | C | T | T=0.188536 | 1.07 | 0.0677 | 2.83E-01 |  |
| rs1561927 | 8 | 129568078 | LINC00824 | T | C | C=0.277668 | 1.10 | 0.0953 | 9.29E-02 |  |
| rs16986825 | 22 | 29300306 | ZNRF3 | T | C | T=0.159933 | 0.99 | -0.0101 | 8.65E-01 |  |
| rs172310 | 7 | 155615627 | SHH, LOC389602 | A | C | A=0.299634 | 1.11 | 0.1044 | 4.98E-02 |  |
| rs17688601 | 7 | 40866663 | SUGCT | C | A | A=0.258063 | 1.12 | 0.1133 | 3.15E-02 |  |
| rs192904742 | 2 | 6722118 | MIR7515HG, LINC01824 | A | G | A=0.010058 | 0.71 | -0.3425 | 4.20E-01 |  |
| rs2417487 | 9 | 106887581 | SMC2 | A | G | G=0.42935 | 1.06 | 0.0583 | 2.43E-01 |  |
| rs2735948 | 5 | 1299213 | TERT, MIR4457 | A | G | A=0.393867 | 1.22 | 0.1989 | 2.81E-05 | LD with rs401681 |
| rs2736098 | 5 | 1294086 | TERT | C | T | T=0.260574 | 1.37 | 0.3148 | 1.97E-08 |  |
| rs2736100 | 5 | 1286516 | TERT | A | C | A=0.493547 | 1.14 | 0.1310 | 5.13E-03 |  |
| rs2816938 | 1 | 199985368 | NR5A2, RNU6-609P | A | T | A=0.297324 | 1.09 | 0.0862 | 1.05E-01 |  |
| rs2853677 | 5 | 1287194 | TERT | A | G | G=0.423081 | 1.14 | 0.1310 | 6.56E-03 | LD with rs2736100 |
| rs28884829 | 11 | 130624371 | LINC02873, AP003486.2 | A | G | A=0.005205 | 1.47 | 0.3853 | 7.06E-03 |  |
| rs2941471 | 8 | 76470404 | HNF4G | A | G | G=0.373796 | 1.04 | 0.0392 | 4.47E-01 |  |
| rs351365 | 1 | 113046395 | WNT2B | C | T | T=0.25846 | 1.09 | 0.0862 | 1.14E-01 |  |
| rs35226131 | 5 | 1295373 | MIR4457, TERT | C | T | T=0.030969 | 1.03 | 0.0296 | 8.27E-01 |  |
| rs3790844 | 1 | 200007432 | NR5A2 | A | G | G=0.24346 | 1.22 | 0.1989 | 5.36E-04 | LD with rs10919791 |
| rs401681 | 5 | 1322087 | CLPTM1L | T | C | T=0.433217 | 1.24 | 0.2151 | 3.45E-06 |  |
| rs450960 | 22 | 18316304 | MICAL3 | T | C | T=0.203399 | 0.95 | -0.0513 | 3.54E-01 |  |
| rs4795218 | 17 | 36078510 | HNF1B | G | A | A=0.214878 | 1.12 | 0.1133 | 6.25E-02 |  |
| rs505922 | 9 | 136149229 | ABO | C | T | C=0.349937 | 1.18 | 0.1655 | 7.25E-04 | LD with rs657152 |
| rs6073450 | 20 | 43086648 | LINC01620 | A | G | A=0.394622 | 1.06 | 0.0583 | 2.50E-01 |  |
| rs657152 | 9 | 136139265 | ABO | A | C | A=0.376266 | 1.20 | 0.1823 | 1.76E-04 |  |
| rs687289 | 9 | 136137106 | ABO | A | G | A=0.349446 | 1.19 | 0.1740 | 3.48E-04 | LD with rs657152 |
| rs6971499 | 7 | 130680521 | AC016831.6, LINC-PINT | T | C | C=0.14662 | 1.32 | 0.2776 | 6.54E-05 |  |
| rs71411601 | 2 | 53012925 | AC010967.1 | C | G | C=0.008311 | 1.55 | 0.4383 | 2.80E-02 |  |
| rs7190458 | 16 | 75263661 | BCAR1 | A | G | A=0.05343 | 1.50 | 0.4055 | 1.56E-05 |  |
| rs7200646 | 16 | 86335351 | LINC00917, LINC01081 | C | T | C=0.348899 | 1.16 | 0.1484 | 2.16E-03 |  |
| rs7214041 | 17 | 70401476 | LINC00511 | T | C | T=0.123737 | 1.15 | 0.1398 | 4.57E-02 |  |
| rs7310409 | 12 | 121424861 | HNF1A | A | G | A=0.403620 | 1.00 | 0.0000 | 9.90E-01 | LD with rs1182933 |
| rs73328514 | 7 | 47488569 | TNS3 | A | T | T=0.134304 | 0.96 | -0.0408 | 5.69E-01 |  |
| rs76974703 | 20 | 56778467 | ANKRD60, C20orf85 | A | G | A=0.016553 | 1.13 | 0.1222 | 6.01E-01 |  |
| rs77038344 | 17 | 38644214 | TNS4 | T | C | T=0.090148 | 1.07 | 0.0677 | 4.76E-01 |  |
| rs77962525 | 4 | 8107943 | ABLIM2 | T | C | T=0.005047 | 1.20 | 0.1823 | 4.35E-01 |  |
| rs8028529 | 15 | 36654597 | MIR4510, C15orf41 | T | C | C=0.224118 | 1.05 | 0.0488 | 4.03E-01 |  |
| rs8176746 | 9 | 136131322 | ANBO | T | G | T=0.085606 | 1.16 | 0.1484 | 8.40E-02 |  |
| rs9543325 | 13 | 73916628 | RNY1P8, AL162376.1 | C | T | C=0.395679 | 1.20 | 0.1823 | 1.08E-04 |  |
| rs9564966 | 13 | 73896221 | KLF5, LINC00392 | A | G | A=0.347615 | 1.13 | 0.1222 | 1.49E-02 | LD with rs9543325 |
| rs9581943 | 13 | 28493997 | PDX1, PLUT | A | G | A=0.3876 | 1.03 | 0.0296 | 5.59E-01 |  |
| rs962856 | 2 | 67593803 | LINC01829 | C | T | C=0.375833 | 1.16 | 0.1484 | 2.05E-03 |  |
| rs9854771 | 3 | 189508471 | TP63 | G | A | A=0.36189 | 1.07 | 0.0677 | 1.44E-01 |  |
| Abbreviations: SNP, single nucleotide polymorphism; PDAC, pancreatic ductal adenocarcinoma; PRS, polygenic risk score; Chr, chromosome; OR, odds ratio; LD, linkage disequilibrium. | | | | | | | | | | |
| ^a^ Position based on GRCh37. | | | | | | | | | | |
| ^b^ MAF, Minor allele frequency in Europeans according to the Allele Frequency Aggregator (ALFA). | | | | | | | | | | |
| Taken from: Sharma S, Tapper WJ, Collins A, Hamady ZZR. Predicting Pancreatic Cancer in the UK Biobank Cohort Using Polygenic Risk Scores and Diabetes Mellitus. Gastroenterology. 2022 May;162(6):1665-1674.e2. | | | | | | | | | | |

| **Table S5. Information on SNPs used to construct PRS 22 for pancreatic cancer** | | | | | | | | | | |
| --- | --- | --- | --- | --- | --- | --- | --- | --- | --- | --- |
| SNP | Chr. | Position^a^ | Gene | Risk allele | Other allele | MAF^b^ | OR^c^ | Beta^c^ | P value^c^ | LD exclusion |
| rs13303010 | 1 | 894573 | NOC2L | G | A | G=0.136619 | 1.26 | 0.2311 | 8.00E-14 |  |
| rs10919791 | 1 | 199965168 | RNU6-716P, AL445687.2 | G | A | A=0.226206 | 0.79 | 0.2357 | 1.40E-11 |  |
| rs2816938 | 1 | 199985368 | NR5A2, RNU6-609P | A | T | A=0.297324 | 1.21 | 0.1906 | 3.00E-15 |  |
| rs1486134 | 2 | 67639769 | LINC02831, ETAA1 | G | T | G=0.278874 | 1.13 | 0.1222 | 5.00E-09 |  |
| rs9854771 | 3 | 189508471 | TP63 | G | A | A=0.36189 | 0.9 | 0.1054 | 5.00E-08 |  |
| rs2736098 | 5 | 1294086 | TERT | C | T | T=0.260574 | 0.84 | 0.1744 | 7.00E-15 |  |
| rs31490 | 5 | 1344458 | CLPTMIL | A | G | A=0.429089 | 1.2 | 0.1823 | 2.00E-11 |  |
| rs35226131 | 5 | 1295373 | MIR4457, TERT | C | T | T=0.030969 | 0.67 | 0.4005 | 2.00E-08 |  |
| rs78417682 | 7 | 47488903 | TNS3 | G | C | C=0.13388 | 0.85 | 0.1625 | 4.35E-08 |  |
| rs17688601 | 7 | 40866663 | SUGCT | C | A | A=0.258063 | 0.88 | 0.1278 | 1.00E-08 |  |
| rs6971499 | 7 | 130680521 | AC016831.6, LINC-PINT | T | C | C=0.14662 | 0.81 | 0.2107 | 7.00E-14 |  |
| rs2941471 | 8 | 76470404 | HNF4G | A | G | G=0.373796 | 0.89 | 0.1165 | 7.00E-10 |  |
| rs10094872 | 8 | 128719884 | CASC11 | T | A | T=0.366278 | 1.14 | 0.131 | 1.00E-09 |  |
| rs1561927 | 8 | 129568078 | LINC00824 | T | C | C=0.277668 | 0.89 | 0.1165 | 7.00E-08 |  |
| rs687289 | 9 | 136137106 | ABO | A | G | A=0.349446 | 1.27 | 0.239 | 1.60E-16 |  |
| rs9581943 | 13 | 28493997 | PDX1, PLUT | A | G | A=0.3876 | 1.15 | 0.1398 | 5.00E-14 |  |
| rs9543325 | 13 | 73916628 | RNY1P8, AL162376.1 | C | T | C=0.395679 | 1.24 | 0.2151 | 1.00E-22 |  |
| rs7190458 | 16 | 75263661 | BCAR1 | A | G | A=0.05343 | 1.36 | 0.3075 | 1.00E-11 |  |
| rs4795218 | 17 | 36078510 | HNF1B | G | A | A=0.214878 | 0.88 | 0.1278 | 1.00E-08 |  |
| rs11655237 | 17 | 70400166 | LINC00511 | T | C | T=0.124143 | 1.25 | 0.2231 | 1.00E-14 |  |
| rs1517037 | 18 | 56878274 | SEC11C, GRP | C | T | T=0.188536 | 0.86 | 0.1508 | 3.00E-08 |  |
| rs16986825 | 22 | 29300306 | ZNRF3 | T | C | T=0.159933 | 1.15 | 0.1398 | 1.00E-08 |  |
| Abbreviations: SNP, single nucleotide polymorphism; PRS, polygenic risk score; Chr, chromosome; OR, odds ratio; LD, linkage disequilibrium. | | | | | | | | | | |
| ^a^Position based on GRCh37 | | | | | | | | | | |
| ^b^MAF, Minor allele frequency in Europeans according to the Allele Frequency Aggregator (ALFA) | | | | | | | | | | |
| ^c^ OR, Beta and P value are derived from the GWAS catalog and original manuscript. | | | | | | | | | | |
| Taken form: Yuan C, Kim J, Wang QL, Lee AA, Babic A; PanScan/PanC4 I-III Consortium; Amundadottir LT, Klein AP, Li D, McCullough ML, Petersen GM, Risch HA, Stolzenberg-Solomon RZ, Perez K, Ng K, Giovannucci EL, Stampfer MJ, Kraft P, Wolpin BM. The age-dependent association of risk factors with pancreatic cancer. Ann Oncol. 2022 Jul;33(7):693-701. doi: 10.1016/j.annonc.2022.03.276. | | | | | | | | | | |

| **Table S6. Information on SNPs used to construct PRS 32 for pancreatic cancer** | | | | | | | | | | |
| --- | --- | --- | --- | --- | --- | --- | --- | --- | --- | --- |
| SNP | Chr. | Position ^a^ | Gene | Risk allele | Other allele | MAF ^b^ | OR^c^ | Beta | P value^d^ | LD exclusion |
| rs505922 | 9 | 136149229 | ABO | C | T | C=0.349937 | 1.27 | 0.2390 | 7.00E-27 |  |
| rs9543325 | 13 | 73916628 | RNY1P8, AL162376.1 | C | T | C=0.395679 | 1.24 | 0.2151 | 1.00E-22 |  |
| rs401681 | 5 | 1322087 | CLPTM1L | T | C | T=0.433217 | 1.19 | 0.1740 | 9.00E-17 |  |
| rs3790844 | 1 | 200007432 | NR5A2 | A | G | G=0.24346 | 1.23 | 0.2070 | 8.00E-16 |  |
| rs2816938 | 1 | 199985368 | NR5A2, RNU6-609P | A | T | A=0.297324 | 1.21 | 0.1906 | 3.00E-15 |  |
| rs2736098 | 5 | 1294086 | TERT | C | T | T=0.260574 | 1.19 | 0.1740 | 7.00E-15 |  |
| rs7214041 | 17 | 70401476 | LINC00511 | T | C | T=0.123737 | 1.25 | 0.2231 | 9.00E-15 |  |
| rs9581943 | 13 | 28493997 | PDX1, PLUT | A | G | A=0.3876 | 1.15 | 0.1398 | 5.00E-14 |  |
| rs6971499 | 7 | 130680521 | AC016831.6, LINC-PINT | T | C | C=0.14662 | 1.23 | 0.2070 | 7.00E-14 |  |
| rs13303010 | 1 | 894573 | NOC2L | G | A | G=0.136619 | 1.26 | 0.2311 | 8.00E-14 |  |
| rs7190458 | 16 | 75263661 | BCAR1 | A | G | A=0.05343 | 1.36 | 0.3075 | 1.00E-11 |  |
| rs2941471 | 8 | 76470404 | HNF4G | A | G | G=0.373796 | 1.12 | 0.1133 | 7.00E-10 |  |
| rs10094872 | 8 | 128719884 | CASC11 | T | A | T=0.366278 | 1.14 | 0.1310 | 1.00E-09 |  |
| rs1486134 | 2 | 67639769 | LINC02831, ETAA1 | G | T | G=0.278874 | 1.13 | 0.1222 | 5.00E-09 |  |
| rs4795218 | 17 | 36078510 | HNF1B | G | A | A=0.214878 | 1.14 | 0.1310 | 1.00E-08 |  |
| rs16986825 | 22 | 29300306 | ZNRF3 | T | C | T=0.159933 | 1.15 | 0.1398 | 1.00E-08 |  |
| rs17688601 | 7 | 40866663 | SUGCT | C | A | A=0.258063 | 1.14 | 0.1310 | 1.00E-08 |  |
| rs35226131 | 5 | 1295373 | MIR4457, TERT | C | T | T=0.030969 | 1.49 | 0.3988 | 2.00E-08 |  |
| rs1517037 | 18 | 56878274 | SEC11C, GRP | C | T | T=0.188536 | 1.16 | 0.1484 | 3.00E-08 |  |
| rs9854771 | 3 | 189508471 | TP63 | G | A | A=0.36189 | 1.11 | 0.1044 | 5.00E-08 |  |
| rs1561927 | 8 | 129568078 | LINC00824 | T | C | C=0.277668 | 1.12 | 0.1133 | 7.00E-08 |  |
| rs73328514 | 7 | 47488569 | TNS3 | A | T | T=0.134304 | 1.18 | 0.1655 | 1.00E-07 |  |
| rs1182933 | 12 | 121454622 | OASL, C12orf43 | T | C | T=0.304076 | 1.11 | 0.1044 | 3.00E-07 |  |
| rs12478462 | 2 | 153654720 | ARL6IP6, AC009969.1 | G | T | G=0.246948 | 1.14 | 0.1310 | 3.00E-07 |  |
| rs2417487 | 9 | 106887581 | SMC2 | A | G | G=0.42935 | 1.09 | 0.0862 | 6.00E-07 |  |
| rs450960 | 22 | 18316304 | MICAL3 | T | C | T=0.203399 | 1.11 | 0.1044 | 8.00E-07 |  |
| rs77038344 | 17 | 38644214 | TNS4 | T | C | T=0.090148 | 1.17 | 0.1570 | 1.00E-06 |  |
| rs7200646 | 16 | 86335351 | LINC00917, LINC01081 | C | T | C=0.348899 | 1.09 | 0.0862 | 4.00E-06 |  |
| rs6537481 | 4 | 148396094 | EDNRA, PRMT5P1 | A | G | G=0.194213 | 1.11 | 0.1044 | 4.00E-06 |  |
| rs6073450 | 20 | 43086648 | LINC01620 | A | G | A=0.394622 | 1.09 | 0.0862 | 5.00E-06 |  |
| rs351365 | 1 | 113046395 | WNT2B | C | T | T=0.25846 | 1.11 | 0.1044 | 6.00E-06 |  |
| rs10991043 | 9 | 106797388 | AL590381.1, SMC2-AS1 | C | T | C=0.376445 | 1.09 | 0.0862 | 7.00E-06 | LD with rs2417487 |
| Abbreviations: SNP, single nucleotide polymorphism; PRS, polygenic risk score; Chr, chromosome; OR, odds ratio; LD, linkage disequilibrium. | | | | | | | | | | |
| ^a^ Position based on GRCh37. | | | | | | | | | | |
| ^b^ MAF, Minor allele frequency in Europeans according to the Allele Frequency Aggregator (ALFA). | | | | | | | | | | |
| ^c^ OR and Beta are derived from GWAS catalog (https://www.ebi.ac.uk/gwas/publications/29422604). | | | | | | | | | | |
| ^d^The P value of each SNP was supplemented using the GWAS catalog and published studies from PubMed. | | | | | | | | | | |
| Taken from: Klein AP, Wolpin BM, Risch HA, Stolzenberg-Solomon RZ, Mocci E, Zhang M, et al. Genome-wide meta-analysis identifies five new susceptibility loci for pancreatic cancer. Nature Communications. 2018;9(1):556. | | | | | | | | | | |

**Table S7. Unweighted healthy lifestyle score components**

| Recommendations ^a^ | Detailed information | Categories | Score |
| --- | --- | --- | --- |
| 1. Be a healthy weight | BMI ^b^ (kg/m^2^) |  |  |
|  | ≥18.5-<24.9 25.0-29.9  <18.5 or ≥30 | Favorable Intermediate Unfavorable | 0.5  0.25  0 |
|  | Waist circumference (cm) |  |  |
|  | Men: <94  Women: <80 Men: 94–<102 Women: 80–<88  Men: ≥102 Women: ≥88 | Favorable Favorable Intermediate Intermediate Unfavorable Unfavorable | 0.5  0.5 0.25  0.25  0  0 |
| 2. Be physically active | Physical activity 10+ min (days/week) |  |  |
|  | Moderate 6-7 or vigorous 3-7 Moderate 1-5 or vigorous 1-2 None | Favorable Intermediate Unfavorable | 0.5  0.25  0 |
|  | Sedentary time ^c^ (hours/day) |  |  |
|  | < 3 3-4.9 ≥ 5 | Favorable Intermediate Unfavorable | 0.5  0.25  0 |
| 3. Eat a diet rich in whole grains, vegetables, fruit and beans | Fruits and vegetables intake ^d^ |  |  |
|  | ≥5 servings/day ≥3-<5 servings/day  <3 servings/day | Favorable Intermediate Unfavorable | 0.5  0.25  0 |
|  | Whole grains intake ^e^ |  |  |
|  | ≥3 servings/day >1.5-<3 servings/day  ≤1.5 serving/day | Favorable Intermediate Unfavorable | 0.5  0.25  0 |
| 4. Limit consumption of red and processed meat | Red meat and processed meat intake |  |  |
|  | ≤2 times/week  >2-≤4 times/week >4 times/week | Favorable Intermediate Unfavorable | 1  0.5  0 |
| 5. Limit alcohol consumption | Alcohol intake frequency ^f^ |  |  |
|  | Never or seldom 1-4 times per week Almost daily | Favorable Intermediate Unfavorable | 1  0.5 0 |
| 6. Additional recommendation | Smoking |  |  |
|  | Never Previous Current | Favorable Intermediate Unfavorable | 1  0.5  0 |
| Total Score Range |  |  | 0-6 |
| ^a^ The unweighted healthy lifestyle score based on the World Cancer Research Fund/American Institute of Cancer Research (WCRF/AICR) lifestyle score and the American Cancer Society (ACS) Guidelines on Nutrition and Physical Activity for Cancer Prevention. | | | |
| ^b^ BMI, body mass index. | | | |
| ^c^ Sedentary time, time spent engaging in sedentary activity (driving, watching TV, computer using). | | | |
| ^d^ Amount per serving: fresh fruit - 1 piece; dried fruit - 5 pieces; cooked/raw/salad vegetables - 3 heaped tablespoons. | | | |
| ^e^ Amount per serving: Bran/oat/muesli cereal - 1 bowl/day; Whole-meal/wholegrain bread - 1 slice/day. | | | |
| ^f^ Seldom, special occasions only, and 1-3 times a month. | | | |

| **Table S8. Baseline characteristics between female and male in the UK Biobank** | | | |
| --- | --- | --- | --- |
| Characteristic | No. (%) | | |
|  | Female | Male | *P* value |
|  | (n = 175,197) | (n = 165,434) |  |
| PC |  |  | <0.001 |
| No | 174,727 (99.7) | 164,775 (99.6) |  |
| Yes | 470 (0.3) | 659 (0.4) |  |
| Age, median (IQR), year | 57 (49-62) | 58 (50-63) | <0.001 |
| Education level |  |  | <0.001 |
| College or University | 59,851 (34.2) | 59,750 (36.1) |  |
| Upper secondary | 22,760 (13.0) | 18,388 (11.1) |  |
| Lower secondary | 51,431 (29.4) | 41,141 (24.9) |  |
| Vocational | 7,218 (4.1) | 15,080 (9.1) |  |
| Other | 33,937 (19.4) | 31,075 (18.8) |  |
| Socioeconomic status |  |  | <0.001 |
| Low | 34,404 (19.6) | 33,725 (20.4) |  |
| Middle | 106,037 (60.5) | 98,251 (59.4) |  |
| High | 34,756 (19.8) | 33,458 (20.2) |  |
| BMI (kg/m^2^) |  |  | <0.001 |
| Unfavorable | 38,988 (22.3) | 40,931 (24.7) |  |
| Intermediate | 63,904 (36.5) | 82,377 (49.8) |  |
| Favorable | 72,305 (41.3) | 42,126 (25.5) |  |
| Waist circumference (cm) |  |  | <0.001 |
| Unfavorable | 58,983 (33.7) | 48,691 (29.4) |  |
| Intermediate | 44,287 (25.3) | 48,255 (29.2) |  |
| Favorable | 71,927 (41.1) | 68,488 (41.4) |  |
| Physical activity 10+ min (days/week) |  |  | <0.001 |
| Unfavorable | 19,500 (11.1) | 18,130 (11.0) |  |
| Intermediate | 82,839 (47.3) | 72,247 (43.7) |  |
| Favorable | 72,858 (41.6) | 75,057 (45.4) |  |
| Sedentary time (hours/day) |  |  | <0.001 |
| Unfavorable | 65,154 (37.2) | 88,225 (53.3) |  |
| Intermediate | 75,821 (43.3) | 57,122 (34.5) |  |
| Favorable | 34,222 (19.5) | 20,087 (12.1) |  |
| Fruit and vegetable intake |  |  | <0.001 |
| Unfavorable | 44,983 (25.7) | 65,068 (39.3) |  |
| Intermediate | 76,539 (43.7) | 63,698 (38.5) |  |
| Favorable | 53,675 (30.6) | 36,668 (22.2) |  |
| Whole grains intake |  |  | <0.001 |
| Unfavorable | 53,578 (30.6) | 24,856 (15.0) |  |
| Intermediate | 86,292 (49.3) | 72,887 (44.1) |  |
| Favorable | 35,327 (20.2) | 67,691 (40.9) |  |
| Red meat and processed meat intake |  |  | <0.001 |
| Unfavorable | 46,576 (26.6) | 78,825 (47.6) |  |
| Intermediate | 65,044 (37.1) | 55,776 (33.7) |  |
| Favorable | 63,577 (36.3) | 30,833 (18.6) |  |
| Alcohol intake frequency |  |  | <0.001 |
| Unfavorable | 30,650 (17.5) | 44,596 (27.0) |  |
| Intermediate | 85,738 (48.9) | 88,748 (53.6) |  |
| Favorable | 58,809 (33.6) | 32,090 (19.4) |  |
| Smoking |  |  | <0.001 |
| Unfavorable | 14,513 (8.3) | 19,399 (11.7) |  |
| Intermediate | 56,001 (32.0) | 63,662 (38.5) |  |
| Favorable | 104,683 (59.8) | 82,373 (49.8) |  |
| PRS |  |  | 0.456 |
| Low | 58,456 (33.4) | 55,069 (33.3) |  |
| Intermediate | 58,242 (33.2) | 55,329 (33.4) |  |
| High | 58,499 (33.4) | 54,036 (33.3) |  |
| Abbreviations: PC, pancreatic cancer; PRS, polygenic risk score; BMI, body mass index. | | | |

| **Table S9. Sex-specific weighted healthy lifestyle score components ^a^** | | | | | | | |
| --- | --- | --- | --- | --- | --- | --- | --- |
| Characteristic | Female | | |  | Male | | |
|  | Estimated log_e_-HR | HR (95%CI) | *P* value |  | Estimated log_e_-HR | HR (95%CI) | *P* value |
| BMI (kg/m^2^) |  |  |  |  |  |  |  |
| Unfavorable | 0 | 1 [Reference] | NA |  | 0 | 1 [Reference] | NA |
| Intermediate | -0.162 | 0.850 (0.657-1.101) | 0.219 |  | -0.060 | 0.942 (0.754-1.177) | 0.599 |
| Favorable | -0.349 | 0.706 (0.501-0.994) | 0.046 |  | -0.083 | 0.921 (0.669-1.268) | 0.613 |
| Waist circumference (cm) |  |  |  |  |  |  |  |
| Unfavorable | 0 | 1 [Reference] | NA |  | 0 | 1 [Reference] | NA |
| Intermediate | -0.061 | 0.941 (0.719-1.231) | 0.656 |  | -0.184 | 0.832 (0.664-1.042) | 0.109 |
| Favorable | 0.026 | 1.026 (0.754-1.397) | 0.868 |  | -0.332 | 0.717 (0.548-0.939) | 0.016 |
| Physical activity 10+ min (days/week) | |  |  |  |  |  |  |
| Unfavorable | 0 | 1 [Reference] | NA |  | 0 | 1 [Reference] | NA |
| Intermediate | -0.079 | 0.924 (0.686-1.243) | 0.601 |  | 0.024 | 1.024 (0.796-1.317) | 0.854 |
| Favorable | -0.028 | 0.972 (0.718-1.315) | 0.854 |  | -0.037 | 0.963 (0.745-1.245) | 0.775 |
| Sedentary time (hours/day) |  |  |  |  |  |  |  |
| Unfavorable | 0 | 1 [Reference] | NA |  | 0 | 1 [Reference] | NA |
| Intermediate | -0.216 | 0.806 (0.658-0.986) | 0.036 |  | -0.083 | 0.920 (0.776-1.092) | 0.341 |
| Favorable | -0.149 | 0.861 (0.657-1.129) | 0.280 |  | -0.261 | 0.770 (0.579-1.025) | 0.073 |
| Fruit and vegetable intake |  |  |  |  |  |  |  |
| Unfavorable | 0 | 1 [Reference] | NA |  | 0 | 1 [Reference] | NA |
| Intermediate | 0.033 | 1.033 (0.819-1.304) | 0.783 |  | -0.084 | 0.919 (0.770-1.097) | 0.35 |
| Favorable | -0.045 | 0.956 (0.742-1.233) | 0.731 |  | -0.057 | 0.944 (0.768-1.162) | 0.587 |
| Whole grains intake |  |  |  |  |  |  |  |
| Unfavorable | 0 | 1 [Reference] | NA |  | 0 | 1 [Reference] | NA |
| Intermediate | -0.225 | 0.799 (0.649-0.982) | 0.033 |  | 0.004 | 1.004 (0.797-1.265) | 0.973 |
| Favorable | -0.282 | 0.754 (0.581-0.980) | 0.035 |  | -0.020 | 0.981 (0.774-1.242) | 0.871 |
| Red meat and processed meat intake | |  |  |  |  |  |  |
| Unfavorable | 0 | 1 [Reference] | NA |  | 0 | 1 [Reference] | NA |
| Intermediate | 0.108 | 1.114 (0.890-1.393) | 0.346 |  | 0.049 | 1.050 (0.885-1.246) | 0.576 |
| Favorable | -0.114 | 0.893 (0.700-1.139) | 0.361 |  | 0.009 | 1.009 (0.809-1.258) | 0.936 |
| Alcohol intake frequency |  |  |  |  |  |  |  |
| Unfavorable | 0 | 1 [Reference] | NA |  | 0 | 1 [Reference] | NA |
| Intermediate | 0.055 | 1.057 (0.822-1.358) | 0.667 |  | -0.204 | 0.815 (0.683-0.974) | 0.024 |
| Favorable | -0.052 | 0.950 (0.723-1.247) | 0.71 |  | -0.041 | 0.959 (0.768-1.198) | 0.714 |
| Smoking |  |  |  |  |  |  |  |
| Unfavorable | 0 | 1 [Reference] | NA |  | 0 | 1 [Reference] | NA |
| Intermediate | -0.661 | 0.517 (0.383-0.696) | <0.001 |  | -0.553 | 0.575 (0.459-0.720) | <0.001 |
| Favorable | -0.747 | 0.474 (0.355-0.632) | <0.001 |  | -0.659 | 0.517 (0.411-0.651) | <0.001 |
| Abbreviations: BMI, body mass index; HR, hazard ratio; NA, not applicable. | | | | | | | |
| ^a^ The Cox proportional hazards regression model (stratified by sex) with all 9 lifestyle factors and adjustment for age (continuous), education level, socioeconomic status, and the first 5 principal components of ancestry. | | | | | | | |

| **Table S10. Baseline characteristics of participants of PC in the UK Biobank** | | | |
| --- | --- | --- | --- |
| Characteristic | No. (%) | | |
|  | Overall | Non-PC | PC |
|  | (N = 340,631) | (n = 339,502) | (n = 1,129) |
| Age, median (IQR), year | 57 (50-63) | 57 (50-63) | 62 (57-66) |
| Sex |  |  |  |
| Female | 175,197 (51.4) | 174,727 (51.5) | 470 (41.6) |
| Male | 165,434 (48.6) | 164,775 (48.5) | 659 (58.4) |
| Education level |  |  |  |
| College or University | 119,601 (35.1) | 119,248 (35.1) | 353 (31.3) |
| Upper secondary | 41,148 (12.1) | 41,047 (12.1) | 101 (8.9) |
| Lower secondary | 92,572 (27.2) | 92,301 (27.2) | 271 (24.0) |
| Vocational | 22,298 (6.5) | 22,207 (6.5) | 91 (8.1) |
| Other | 65,012 (19.1) | 64,699 (19.1) | 313 (27.7) |
| Socioeconomic status |  |  |  |
| Low | 68,129 (20.0) | 67,889 (20.0) | 240 (21.3) |
| Middle | 204,288 (60.0) | 203,611 (60.0) | 677 (60.0) |
| High | 68,214 (20.0) | 68,002 (20.0) | 212 (18.8) |
| BMI (kg/m^2^) |  |  |  |
| Unfavorable | 79,919 (23.5) | 79,588 (23.4) | 331 (29.3) |
| Intermediate | 146,281 (42.9) | 145,770 (42.9) | 511 (45.3) |
| Favorable | 114,431 (33.6) | 114,144 (33.6) | 287 (25.4) |
| Waist circumference (cm) |  |  |  |
| Unfavorable | 107,674 (31.6) | 107,219 (31.6) | 455 (40.3) |
| Intermediate | 92,542 (27.2) | 92,232 (27.2) | 310 (27.5) |
| Favorable | 140,415 (41.2) | 140,051 (41.3) | 364 (32.2) |
| Physical activity 10+ min (days/week) |  |  |  |
| Unfavorable | 37,630 (11.0) | 37,496 (11.0) | 134 (11.9) |
| Intermediate | 155,086 (45.5) | 154,568 (45.5) | 518 (45.9) |
| Favorable | 147,915 (43.4) | 147,438 (43.4) | 477 (42.2) |
| Sedentary time (hours/day) |  |  |  |
| Unfavorable | 153,379 (45.0) | 152,771 (45.0) | 608 (53.9) |
| Intermediate | 132,943 (39.0) | 132,555 (39.0) | 388 (34.4) |
| Favorable | 54,309 (15.9) | 54,176 (16.0) | 133 (11.8) |
| Fruit and vegetable intake |  |  |  |
| Unfavorable | 110,051 (32.3) | 109,675 (32.3) | 376 (33.3) |
| Intermediate | 140,237 (41.2) | 139,780 (41.2) | 457 (40.5) |
| Favorable | 90,343 (26.5) | 90,047 (26.5) | 296 (26.2) |
| Whole grains intake |  |  |  |
| Unfavorable | 78,434 (23.0) | 78,181 (23.0) | 253 (22.4) |
| Intermediate | 159,179 (46.7) | 158,663 (46.7) | 516 (45.7) |
| Favorable | 103,018 (30.2) | 102,658 (30.2) | 360 (31.9) |
| Red meat and processed meat intake | |  |  |
| Unfavorable | 125,401 (36.8) | 124,959 (36.8) | 442 (39.1) |
| Intermediate | 120,820 (35.5) | 120,390 (35.5) | 430 (38.1) |
| Favorable | 94,410 (27.7) | 94,153 (27.7) | 257 (22.8) |
| Alcohol intake frequency |  |  |  |
| Unfavorable | 75,246 (22.1) | 74,938 (22.1) | 308 (27.3) |
| Intermediate | 174,486 (51.2) | 173,959 (51.2) | 527 (46.7) |
| Favorable | 90,899 (26.7) | 90,605 (26.7) | 294 (26.0) |
| Smoking |  |  |  |
| Unfavorable | 33,912 (10.0) | 33,737 (9.9) | 175 (15.5) |
| Intermediate | 119,663 (35.1) | 119,208 (35.1) | 455 (40.3) |
| Favorable | 187,056 (54.9) | 186,557 (55.0) | 499 (44.2) |
| Unweighted healthy lifestyle score |  |  |  |
| Unfavorable | 116,605 (34.2) | 116,096 (34.2) | 509 (45.1) |
| Intermediate | 102,246 (30.0) | 101,907 (30.0) | 339 (30.0) |
| Favorable | 121,780 (35.8) | 121,499 (35.8) | 281 (24.9) |
| Weighted healthy lifestyle score |  |  |  |
| Unfavorable | 113,431 (33.3) | 112,905 (33.3) | 526 (46.6) |
| Intermediate | 113,858 (33.4) | 113,497 (33.4) | 361 (32.0) |
| Favorable | 113,342 (33.3) | 113,100 (33.3) | 242 (21.4) |
| PRS |  |  |  |
| Low | 113,525 (33.3) | 113,308 (33.4) | 217 (19.2) |
| Intermediate | 113,571 (33.3) | 113,207 (33.3) | 364 (32.2) |
| High | 113,535 (33.3) | 112,987 (33.3) | 548 (48.5) |
| Abbreviations: PC, pancreatic cancer; BMI, body mass index; PRS, polygenic risk score. | | | |

| **Table S11. Associations between lifestyle components and PC** | | | | | | | |
| --- | --- | --- | --- | --- | --- | --- | --- |
| Characteristic | Model 1^a^ | | |  | Model 2^a^ | | |
|  | HR (95% CI) | *P* value | *P* value for trend |  | HR (95% CI) | *P* value | *P* value for trend |
| BMI (kg/m^2^) |  |  | <0.001 |  |  |  | <0.001 |
| Unfavorable | 1 [Reference] | NA |  |  | 1 [Reference] | NA |  |
| Intermediate | 0.84 (0.73-0.96) | 0.011 |  |  | 0.81 (0.71-0.93) | 0.003 |  |
| Favorable | 0.60 (0.51-0.70) | <0.001 |  |  | 0.69 (0.58-0.81) | <0.001 |  |
| Waist circumference (cm) |  |  | <0.001 |  |  |  | <0.001 |
| Unfavorable | 1 [Reference] | NA |  |  | 1 [Reference] | NA |  |
| Intermediate | 0.78 (0.68-0.91) | 0.001 |  |  | 0.80 (0.69-0.93) | 0.003 |  |
| Favorable | 0.61 (0.53-0.69) | <0.001 |  |  | 0.71 (0.61-0.81) | <0.001 |  |
| Physical activity 10+ min (days/week) |  |  | 0.282 |  |  |  | 0.110 |
| Unfavorable | 1 [Reference] | NA |  |  | 1 [Reference] | NA |  |
| Intermediate | 0.93 (0.77-1.12) | 0.443 |  |  | 0.91 (0.75-1.10) | 0.339 |  |
| Favorable | 0.90 (0.74-1.09) | 0.267 |  |  | 0.86 (0.71-1.04) | 0.118 |  |
| Sedentary time (hours/day) |  |  | <0.001 |  |  |  | <0.001 |
| Unfavorable | 1 [Reference] | NA |  |  | 1 [Reference] | NA |  |
| Intermediate | 0.73 (0.64-0.83) | <0.001 |  |  | 0.82 (0.72-0.93) | 0.002 |  |
| Favorable | 0.61 (0.50-0.73) | <0.001 |  |  | 0.75 (0.62-0.90) | 0.003 |  |
| Fruit and vegetable intake |  |  | 0.517 |  |  |  | 0.077 |
| Unfavorable | 1 [Reference] | NA |  |  | 1 [Reference] | NA |  |
| Intermediate | 0.95 (0.83-1.09) | 0.442 |  |  | 0.90 (0.78-1.03) | 0.130 |  |
| Favorable | 0.95 (0.82-1.11) | 0.540 |  |  | 0.87 (0.75-1.02) | 0.082 |  |
| Whole grains intake |  |  | 0.260 |  |  |  |  |
| Unfavorable | 1 [Reference] | NA |  |  | 1 [Reference] | NA | 0.026 |
| Intermediate | 1.01 (0.87-1.17) | 0.934 |  |  | 0.86 (0.74-1.00) | 0.058 |  |
| Favorable | 1.09 (0.93-1.28) | 0.292 |  |  | 0.82 (0.70-0.97) | 0.020 |  |
| Red meat and processed meat intake |  |  | 0.001 |  |  |  | 0.123 |
| Unfavorable | 1 [Reference] | NA |  |  | 1 [Reference] | NA |  |
| Intermediate | 1.00 (0.88-1.15) | 0.952 |  |  | 1.03 (0.90-1.18) | 0.628 |  |
| Favorable | 0.76 (0.66-0.89) | 0.001 |  |  | 0.87 (0.74-1.02) | 0.080 |  |
| Alcohol intake frequency |  |  | 0.005 |  |  |  | 0.245 |
| Unfavorable | 1 [Reference] | NA |  |  | 1 [Reference] | NA |  |
| Intermediate | 0.73 (0.63-0.84) | <0.001 |  |  | 0.86 (0.74-0.99) | 0.033 |  |
| Favorable | 0.79 (0.67-0.93) | 0.004 |  |  | 0.91 (0.77-1.07) | 0.252 |  |
| Smoking |  |  | <0.001 |  |  |  | <0.001 |
| Unfavorable | 1 [Reference] | NA |  |  | 1 [Reference] | NA |  |
| Intermediate | 0.72 (0.61-0.86) | <0.001 |  |  | 0.56 (0.47-0.66) | <0.001 |  |
| Favorable | 0.50 (0.42-0.59) | <0.001 |  |  | 0.48 (0.40-0.57) | <0.001 |  |
| Abbreviations: PC, pancreatic cancer; BMI, body mass index; HR, hazard ratio; NA, not applicable. | | | | | | | |
| ^a^ Model 1 was not adjusted; model 2 was adjusted for age (continuous), sex, education level, socioeconomic status, and the first 5 principal components of ancestry. | | | | | | | |

| **Table S12. Combined association between PRS, weighted healthy lifestyle score, and PC** | | | | | | |
| --- | --- | --- | --- | --- | --- | --- |
| Characteristic | Events/person-year | Model 1^a^ | |  | Model 2^a^ | |
|  |  | HR (95% CI) | *P* value |  | HR (95% CI) | *P* value |
| Low PRS |  |  |  |  |  |  |
| Favorable lifestyle | 46/487,929 | 0.53 (0.37-0.76) | <0.001 |  | 0.53 (0.37-0.76) | <0.001 |
| Intermediate lifestyle | 72/490,504 | 0.82 (0.60-1.13) | 0.226 |  | 0.76 (0.56-1.04) | 0.083 |
| Unfavorable lifestyle | 99/477,130 | 1.17 (0.88-1.56) | 0.290 |  | 1.09 (0.82-1.45) | 0.565 |
| Intermediate PRS |  |  |  |  |  |  |
| Favorable lifestyle | 87/488,588 | 1 [Reference] | NA |  | 1 [Reference] | NA |
| Intermediate lifestyle | 109/487,417 | 1.26 (0.95-1.67) | 0.112 |  | 1.16 (0.87-1.54) | 0.307 |
| Unfavorable lifestyle | 168/478,788 | 1.98 (1.53-2.56) | <0.001 |  | 1.85 (1.43-2.41) | <0.001 |
| High PRS |  |  |  |  |  |  |
| Favorable lifestyle | 109/488,047 | 1.25 (0.95-1.66) | 0.115 |  | 1.27 (0.95-1.68) | 0.102 |
| Intermediate lifestyle | 180/484,195 | 2.09 (1.62-2.70) | <0.001 |  | 1.93 (1.50-2.50) | <0.001 |
| Unfavorable lifestyle | 259/480,832 | 3.03 (2.38-3.87) | <0.001 |  | 2.84 (2.22-3.62) | <0.001 |
| Abbreviations: PC, pancreatic cancer; PRS, polygenic risk score; HR, hazard ratio; NA, not applicable. | | | | | | |
| ^a^ Model 1 was not adjusted; model 2 was adjusted for age (continuous), sex, education level, socioeconomic status, and the first 5 principal components of ancestry. | | | | | | |

| **Table S13. Combined association between PRS, unweighted healthy lifestyle score, and PC** | | | | | | |
| --- | --- | --- | --- | --- | --- | --- |
| Characteristic | Events/person-year | Model 1^a^ | |  | Model 2^a^ | |
|  |  | HR (95% CI) | *P* value |  | HR (95% CI) | *P* value |
| Low PRS |  |  |  |  |  |  |
| Favorable lifestyle | 50/524,629 | 0.54 (0.38-0.77) | <0.001 |  | 0.55 (0.39-0.77) | <0.001 |
| Intermediate lifestyle | 73/438,487 | 0.95 (0.70-1.29) | 0.736 |  | 0.88 (0.65-1.20) | 0.422 |
| Unfavorable lifestyle | 94/492,448 | 1.09 (0.82-1.45) | 0.558 |  | 0.94 (0.70-1.26) | 0.675 |
| Intermediate PRS |  |  |  |  |  |  |
| Favorable lifestyle | 92/524,048 | 1 [Reference] | NA |  | 1 [Reference] | NA |
| Intermediate lifestyle | 101/439,546 | 1.31 (0.99-1.74) | 0.061 |  | 1.23 (0.92-1.63) | 0.159 |
| Unfavorable lifestyle | 171/491,200 | 1.99 (1.54-2.56) | <0.001 |  | 1.73 (1.34-2.23) | <0.001 |
| High PRS |  |  |  |  |  |  |
| Favorable lifestyle | 139/523,757 | 1.51 (1.16-1.97) | 0.002 |  | 1.52 (1.17-1.98) | 0.002 |
| Intermediate lifestyle | 165/434,691 | 2.16 (1.68-2.79) | <0.001 |  | 2.03 (1.57-2.61) | <0.001 |
| Unfavorable lifestyle | 244/494,627 | 2.82 (2.22-3.58) | <0.001 |  | 2.45 (1.92-3.12) | <0.001 |
| Abbreviations: PC, pancreatic cancer; PRS, polygenic risk score; HR, hazard ratio; NA, not applicable. | | | | | | |
| ^a^ Model 1 was not adjusted; model 2 was adjusted for age (continuous), sex, education level, socioeconomic status, and the first 5 principal components of ancestry. | | | | | | |

| **Table S14. Combined association between PRS, lifestyle components, and PC** | | | | | | | | |
| --- | --- | --- | --- | --- | --- | --- | --- | --- |
| Characteristic | PRS | | | | | | | |
|  | Low | |  | Intermediate | |  | High | |
|  | HR (95% CI) | *P* value |  | HR (95% CI) | *P* value |  | HR (95% CI) | *P* value |
| BMI (kg/m^2^) |  |  |  |  |  |  |  |  |
| Favorable | 0.55 (0.39-0.77) | <0.001 |  | 1 [Reference] | NA |  | 1.45 (1.12-1.88) | 0.005 |
| Intermediate | 0.66 (0.50-0.88) | 0.004 |  | 1.11 (0.86-1.43) | 0.425 |  | 1.79 (1.41-2.26) | <0.001 |
| Unfavorable | 0.90 (0.66-1.23) | 0.521 |  | 1.43 (1.08-1.88) | 0.012 |  | 2.03 (1.57-2.62) | <0.001 |
| Waist circumference (cm) |  |  |  |  |  |  |  |  |
| Favorable | 0.60 (0.44-0.80) | <0.001 |  | 1 [Reference] | NA |  | 1.44 (1.14-1.81) | 0.002 |
| Intermediate | 0.68 (0.50-0.92) | 0.013 |  | 1.09 (0.84-1.43) | 0.505 |  | 1.67 (1.31-2.13) | <0.001 |
| Unfavorable | 0.78 (0.59-1.04) | 0.09 |  | 1.36 (1.07-1.74) | 0.012 |  | 2.13 (1.70-2.66) | <0.001 |
| Physical activity 10+ min (days/week) | |  |  |  |  |  |  |  |
| Favorable | 0.58 (0.44-0.76) | <0.001 |  | 1 [Reference] | NA |  | 1.71 (1.39-2.10) | <0.001 |
| Intermediate | 0.71 (0.55-0.91) | 0.007 |  | 1.18 (0.94-1.47) | 0.150 |  | 1.61 (1.31-1.98) | <0.001 |
| Unfavorable | 0.75 (0.49-1.14) | 0.176 |  | 1.25 (0.90-1.76) | 0.188 |  | 1.81 (1.35-2.43) | <0.001 |
| Sedentary time (hours/day) |  |  |  |  |  |  |  |  |
| Favorable | 0.47 (0.29-0.76) | 0.002 |  | 1 [Reference] | NA |  | 1.04 (0.71-1.52) | 0.833 |
| Intermediate | 0.46 (0.32-0.67) | <0.001 |  | 0.89 (0.64-1.23) | 0.473 |  | 1.41 (1.04-1.91) | 0.026 |
| Unfavorable | 0.70 (0.51-0.96) | 0.290 |  | 1.03 (0.76-1.40) | 0.853 |  | 1.64 (1.22-2.20) | 0.001 |
| Total fruit and vegetable intake |  |  |  |  |  |  |  |  |
| Favorable | 0.61 (0.44-0.85) | 0.003 |  | 1 [Reference] | NA |  | 1.51 (1.17-1.96) | 0.002 |
| Intermediate | 0.65 (0.49-0.87) | 0.004 |  | 0.95 (0.73-1.23) | 0.677 |  | 1.63 (1.28-2.07) | <0.001 |
| Unfavorable | 0.62 (0.45-0.85) | 0.003 |  | 1.28 (0.98-1.67) | 0.065 |  | 1.67 (1.30-2.15) | <0.001 |
| Whole grains intake |  |  |  |  |  |  |  |  |
| Favorable | 0.59 (0.44-0.80) | <0.001 |  | 1 [Reference] | NA |  | 1.52 (1.20-1.92) | <0.001 |
| Intermediate | 0.60 (0.45-0.78) | <0.001 |  | 1.07 (0.84-1.35) | 0.583 |  | 1.60 (1.28-1.99) | <0.001 |
| Unfavorable | 0.80 (0.57-1.11) | 0.173 |  | 1.17 (0.88-1.57) | 0.273 |  | 1.80 (1.39-2.33) | <0.001 |
| Red meat and processed meat intake | |  |  |  |  |  |  |  |
| Favorable | 0.67 (0.47-0.96) | 0.027 |  | 1 [Reference] | NA |  | 1.69 (1.27-2.24) | <0.001 |
| Intermediate | 0.70 (0.51-0.96) | 0.029 |  | 1.32 (1.00-1.74) | 0.053 |  | 1.99 (1.53-2.58) | <0.001 |
| Unfavorable | 0.78 (0.58-1.06) | 0.116 |  | 1.28 (0.97-1.69) | 0.084 |  | 1.81 (1.39-2.36) | <0.001 |
| Alcohol intake frequency |  |  |  |  |  |  |  |  |
| Favorable | 0.61 (0.44-0.85) | 0.003 |  | 1 [Reference] | NA |  | 1.58 (1.21-2.04) | <0.001 |
| Intermediate | 0.61 (0.46-0.80) | <0.001 |  | 1.02 (0.79-1.31) | 0.874 |  | 1.38 (1.09-1.76) | 0.008 |
| Unfavorable | 0.61 (0.43-0.85) | 0.004 |  | 1.06 (0.79-1.41) | 0.715 |  | 1.85 (1.43-2.39) | <0.001 |
| Smoking |  |  |  |  |  |  |  |  |
| Favorable | 0.57 (0.44-0.73) | <0.001 |  | 1 [Reference] | NA |  | 1.37 (1.12-1.67) | 0.002 |
| Intermediate | 0.62 (0.48-0.81) | <0.001 |  | 1.09 (0.87-1.36) | 0.443 |  | 1.70 (1.39-2.07) | <0.001 |
| Unfavorable | 1.25 (0.87-1.80) | 0.226 |  | 1.70 (1.23-2.34) | 0.001 |  | 3.18 (2.46-4.11) | <0.001 |
| Abbreviations: PC, pancreatic cancer; PRS, polygenic risk score; HR, hazard ratio; BMI, body mass index; NA, not applicable. | | | | | | | | |
| Model was adjusted for age (continuous), sex, education level, socioeconomic status, and the first 5 principal components of ancestry. | | | | | | | | |

| **Table S15. The absolute risk reductions of pancreatic cancer in different PRS** | | | | | | | |
| --- | --- | --- | --- | --- | --- | --- | --- |
| Characteristic | Weighted healthy lifestyle score | | | | | | |
|  | Unfavorable | Intermediate | Absolute risk reduction |  | Unfavorable | Favorable | Absolute risk reduction |
|  | events/No. (%) | events/No. (%) | (95%CI) |  | events/No. (%) | events/No. (%) | (95%CI) |
| PRS |  |  |  |  |  |  |  |
| Low | 99/37,640 (0.26) | 72/38,161 (0.19) | 0.07 (0.01-0.14) |  | 99/37,640 (0.26) | 46/37,724 (0.12) | 0.14 (0.08-0.20) |
| Intermediate | 168/37,798 (0.44) | 109/37,958 (0.29) | 0.16 (0.07-0.24) |  | 168/37,798 (0.44) | 87/37,815 (0.23) | 0.21 (0.13-0.30) |
| High | 259/37,993 (0.68) | 180/37,739 (0.48) | 0.20 (0.10-0.31) |  | 259/37,993 (0.68) | 109/37,803 (0.29) | 0.39 (0.29-0.49) |
| Characteristic | Unweighted healthy lifestyle score | | | | | | |
|  | Unfavorable | Intermediate | Absolute risk reduction |  | Unfavorable | Favorable | Absolute risk reduction |
|  | events/No. (%) | events/No. (%) | (95%CI) |  | events/No. (%) | events/No. (%) | (95%CI) |
| PRS |  |  |  |  |  |  |  |
| Low | 94/38,792 (0.24) | 73/34,148 (0.21) | 0.03 (-0.04-0.10) |  | 94/38,792 (0.24) | 50/40,585 (0.12) | 0.12 (0.06-0.18) |
| Intermediate | 171/38,773 (0.44) | 101/34,203 (0.30) | 0.15 (0.06-0.23) |  | 171/38,773 (0.44) | 92/40,595 (0.23) | 0.21 (0.13-0.29) |
| High | 244/39,040 (0.63) | 165/33,895 (0.49) | 0.14 (0.03-0.25) |  | 244/39,040 (0.63) | 139/40,600 (0.34) | 0.28 (0.19-0.38) |
| Abbreviations: PC, pancreatic cancer; PRS, polygenic risk score. | | | | | | | |

| **Table S16. Multivariable Cox regression analysis of genetic risk and lifestyles in relation to risk of PC, stratified by age ^a^** | | | | | | | | | |
| --- | --- | --- | --- | --- | --- | --- | --- | --- | --- |
| Characteristic | Age | | | | | | | | *P* value for interaction |
|  | ≤60 | |  | 61-70 | |  | >70 | |  |
|  | HR (95% CI) | *P* value |  | HR (95% CI) | *P* value |  | HR (95% CI) | *P* value |  |
| PRS |  |  |  |  |  |  |  |  | 0.164 |
| Low | 0.61 (0.37-0.99) | 0.043 |  | 0.61 (0.47-0.80) | <0.001 |  | 0.58 (0.45-0.73) | <0.001 |  |
| Intermediate | 1 [Reference] | NA |  | 1 [Reference] | NA |  | 1 [Reference] | NA |  |
| High | 1.89 (1.30-2.73) | <0.001 |  | 1.70 (1.38-2.09) | <0.001 |  | 1.27 (1.04-1.54) | 0.018 |  |
| Unweighted healthy lifestyle score |  |  |  |  |  |  |  |  | 0.290 |
| Unfavorable | 1 [Reference] | NA |  | 1 [Reference] | NA |  | 1 [Reference] | NA |  |
| Intermediate | 0.60 (0.41-0.87) | 0.008 |  | 0.83 (0.67-1.03) | 0.089 |  | 0.86 (0.70-1.05) | 0.145 |  |
| Favorable | 0.39 (0.26-0.59) | <0.001 |  | 0.62 (0.49-0.79) | <0.001 |  | 0.66 (0.52-0.82) | <0.001 |  |
| Weighted healthy lifestyle score |  |  |  |  |  |  |  |  | 0.244 |
| Unfavorable | 1 [Reference] | NA |  | 1 [Reference] | NA |  | 1 [Reference] | NA |  |
| Intermediate | 0.49 (0.33-0.72) | <0.001 |  | 0.67 (0.54-0.83) | <0.001 |  | 0.73 (0.60-0.89) | 0.002 |  |
| Favorable | 0.35 (0.23-0.54) | <0.001 |  | 0.50 (0.39-0.63) | <0.001 |  | 0.52 (0.41-0.65) | <0.001 |  |
| PRS and unweighted healthy lifestyle score |  |  |  |  |  |  |  |  | 0.086 |
| Low |  |  |  |  |  |  |  |  |  |
| favorable | 0.41 (0.15-1.17) | 0.096 |  | 0.60 (0.35-1.03) | 0.064 |  | 0.54 (0.33-0.88) | 0.014 |  |
| Intermediate | 0.73 (0.29-1.86) | 0.509 |  | 0.79 (0.47-1.32) | 0.372 |  | 0.98 (0.64-1.50) | 0.933 |  |
| unfavorable | 1.35 (0.62-2.94) | 0.446 |  | 1.07 (0.68-1.69) | 0.755 |  | 0.74 (0.48-1.15) | 0.182 |  |
| Intermediate |  |  |  |  |  |  |  |  |  |
| favorable | 1 [Reference] | NA |  | 1 [Reference] | NA |  | 1 [Reference] | NA |  |
| Intermediate | 0.52 (0.18-1.47) | 0.215 |  | 1.43 (0.92-2.22) | 0.116 |  | 1.23 (0.82-1.84) | 0.311 |  |
| unfavorable | 2.49 (1.25-4.97) | 0.010 |  | 1.63 (1.07-2.47) | 0.022 |  | 1.63 (1.13-2.35) | 0.009 |  |
| High |  |  |  |  |  |  |  |  |  |
| favorable | 1.41 (0.67-2.96) | 0.361 |  | 1.81 (1.19-2.73) | 0.005 |  | 1.33 (0.90-1.96) | 0.147 |  |
| Intermediate | 3.06 (1.56-6.00) | 0.001 |  | 2.32 (1.55-3.47) | <0.001 |  | 1.55 (1.05-2.27) | 0.026 |  |
| unfavorable | 3.33 (1.72-6.46) | <0.001 |  | 2.75 (1.87-4.05) | <0.001 |  | 2.01 (1.41-2.87) | <0.001 |  |
| PRS and weighted healthy lifestyle score |  |  |  |  |  |  |  |  | 0.010 |
| Low |  |  |  |  |  |  |  |  |  |
| favorable | 1.00 (0.35-2.84) | 0.996 |  | 0.33 (0.17-0.61) | <0.001 |  | 0.65 (0.40-1.06) | 0.085 |  |
| Intermediate | 0.48 (0.12-1.87) | 0.291 |  | 0.58 (0.35-0.96) | 0.034 |  | 0.95 (0.62-1.46) | 0.807 |  |
| unfavorable | 2.50 (1.03-6.10) | 0.044 |  | 1.18 (0.78-1.79) | 0.441 |  | 0.77 (0.48-1.22) | 0.261 |  |
| Intermediate |  |  |  |  |  |  |  |  |  |
| favorable | 1 [Reference] | NA |  | 1 [Reference] | NA |  | 1 [Reference] | NA |  |
| Intermediate | 2.26 (0.91-5.60) | 0.078 |  | 0.92 (0.59-1.43) | 0.705 |  | 1.22 (0.81-1.83) | 0.345 |  |
| unfavorable | 3.40 (1.45-7.98) | 0.005 |  | 1.52 (1.02-2.26) | 0.039 |  | 1.91 (1.31-2.80) | <0.001 |  |
| High |  |  |  |  |  |  |  |  |  |
| favorable | 2.43 (1.01-5.85) | 0.048 |  | 1.28 (0.85-1.94) | 0.242 |  | 1.04 (0.68-1.61) | 0.846 |  |
| Intermediate | 3.41 (1.45-8.02) | 0.005 |  | 2.01 (1.38-2.93) | <0.001 |  | 1.63 (1.11-2.40) | 0.013 |  |
| unfavorable | 6.69 (2.99-14.93) | <0.001 |  | 2.51 (1.75-3.62) | <0.001 |  | 2.52 (1.75-3.63) | <0.001 |  |
| Abbreviations: PC, pancreatic cancer; PRS, polygenic risk score; HR, hazard ratio; NA, not applicable. | | | | | | | | | |
| ^a^ Model was adjusted for sex, education level, socioeconomic status, and the first 5 principal components of ancestry. | | | | | | | | | |

| **Table S17. Multivariable Cox regression analysis of lifestyle components in relation to risk of PC, stratified by age ^a^** | | | | | | | | |
| --- | --- | --- | --- | --- | --- | --- | --- | --- |
| Characteristic | Age | | | | | | | |
|  | ≤60 | |  | 61-70 | |  | >70 | |
|  | HR (95% CI) | *P* value |  | HR (95% CI) | *P* value |  | HR (95% CI) | *P* value |
| BMI (kg/m^2^) |  |  |  |  |  |  |  |  |
| Unfavorable | 1 [Reference] | NA |  | 1 [Reference] | NA |  | 1 [Reference] | NA |
| Intermediate | 0.74 (0.51-1.08) | 0.121 |  | 0.79 (0.64-0.98) | 0.032 |  | 0.87 (0.71-1.08) | 0.203 |
| Favorable | 0.49 (0.31-0.75) | 0.001 |  | 0.69 (0.54-0.88) | 0.003 |  | 0.76 (0.59-0.97) | 0.027 |
| Waist circumference (cm) |  |  |  |  |  |  |  |  |
| Unfavorable | 1 [Reference] | NA |  | 1 [Reference] | NA |  | 1 [Reference] | NA |
| Intermediate | 0.57 (0.37-0.86) | 0.008 |  | 0.81 (0.65-1.02) | 0.070 |  | 0.86 (0.70-1.07) | 0.174 |
| Favorable | 0.48 (0.33-0.69) | <0.001 |  | 0.71 (0.57-0.87) | 0.001 |  | 0.77 (0.63-0.95) | 0.015 |
| Physical activity 10+ min (days/week) |  |  |  |  |  |  |  |  |
| Unfavorable | 1 [Reference] | NA |  | 1 [Reference] | NA |  | 1 [Reference] | NA |
| Intermediate | 1.39 (0.77-2.51) | 0.282 |  | 0.80 (0.60-1.06) | 0.112 |  | 0.96 (0.72-1.28) | 0.757 |
| Favorable | 1.34 (0.74-2.43) | 0.335 |  | 0.86 (0.65-1.13) | 0.280 |  | 0.79 (0.59-1.07) | 0.123 |
| Sedentary time (hours/day) |  |  |  |  |  |  |  |  |
| Unfavorable | 1 [Reference] | NA |  | 1 [Reference] | NA |  | 1 [Reference] | NA |
| Intermediate | 0.93 (0.66-1.32) | 0.690 |  | 0.94 (0.77-1.15) | 0.555 |  | 0.68 (0.55-0.82) | <0.001 |
| Favorable | 0.57 (0.33-0.99) | 0.044 |  | 0.85 (0.63-1.13) | 0.256 |  | 0.71 (0.53-0.95) | 0.020 |
| Fruit and vegetable intake |  |  |  |  |  |  |  |  |
| Unfavorable | 1 [Reference] | NA |  | 1 [Reference] | NA |  | 1 [Reference] | NA |
| Intermediate | 1.04 (0.72-1.49) | 0.853 |  | 0.92 (0.75-1.14) | 0.458 |  | 0.87 (0.70-1.07) | 0.194 |
| Favorable | 0.99 (0.64-1.52) | 0.959 |  | 0.77 (0.60-0.98) | 0.033 |  | 0.98 (0.78-1.23) | 0.853 |
| Whole grains intake |  |  |  |  |  |  |  |  |
| Unfavorable | 1 [Reference] | NA |  | 1 [Reference] | NA |  | 1 [Reference] | NA |
| Intermediate | 1.19 (0.80-1.79) | 0.389 |  | 0.75 (0.60-0.95) | 0.016 |  | 0.91 (0.72-1.15) | 0.438 |
| Favorable | 0.99 (0.62-1.58) | 0.956 |  | 0.83 (0.65-1.06) | 0.134 |  | 0.81 (0.63-1.04) | 0.102 |
| Red meat and processed meat intake | |  |  |  |  |  |  |  |
| Unfavorable | 1 [Reference] | NA |  | 1 [Reference] | NA |  | 1 [Reference] | NA |
| Intermediate | 1.09 (0.76-1.57) | 0.628 |  | 1.04 (0.84-1.29) | 0.702 |  | 1.02 (0.83-1.24) | 0.872 |
| Favorable | 0.61 (0.38-0.96) | 0.032 |  | 0.98 (0.77-1.24) | 0.870 |  | 0.85 (0.67-1.08) | 0.175 |
| Alcohol intake frequency |  |  |  |  |  |  |  |  |
| Unfavorable | 1 [Reference] | NA |  | 1 [Reference] | NA |  | 1 [Reference] | NA |
| Intermediate | 0.63 (0.42-0.93) | 0.021 |  | 0.90 (0.72-1.11) | 0.316 |  | 0.85 (0.69-1.06) | 0.142 |
| Favorable | 0.67 (0.42-1.06) | 0.085 |  | 0.80 (0.61-1.04) | 0.090 |  | 1.08 (0.85-1.37) | 0.552 |
| Smoking |  |  |  |  |  |  |  |  |
| Unfavorable | 1 [Reference] | NA |  | 1 [Reference] | NA |  | 1 [Reference] | NA |
| Intermediate | 0.52 (0.34-0.81) | 0.004 |  | 0.64 (0.49-0.84) | 0.001 |  | 0.57 (0.43-0.75) | <0.001 |
| Favorable | 0.38 (0.25-0.57) | <0.001 |  | 0.54 (0.41-0.71) | <0.001 |  | 0.51 (0.38-0.67) | <0.001 |
| Abbreviations: PC, pancreatic cancer; BMI, body mass index; HR, hazard ratio; NA, not applicable. | | | | | | | | |
| ^a^ Model was adjusted for sex, education level, socioeconomic status, and the first 5 principal components of ancestry. | | | | | | | | |

| **Table S18. Multivariable Cox regression analysis of genetic risk and lifestyles in relation to risk of PC, stratified by sex ^a^** | | | | | | |
| --- | --- | --- | --- | --- | --- | --- |
| Characteristic | Sex | | | | | *P* value for interaction |
|  | Female | |  | Male | |  |
|  | HR (95% CI) | *P* value |  | HR (95% CI) | *P* value |  |
| PRS |  |  |  |  |  | 0.553 |
| Low | 0.66 (0.51-0.85) | 0.002 |  | 0.55 (0.44-0.69) | <0.001 |  |
| Intermediate | 1 [Reference] | NA |  | 1 [Reference] | NA |  |
| High | 1.55 (1.26-1.91) | <0.001 |  | 1.48 (1.25-1.76) | <0.001 |  |
| Unweighted healthy lifestyle score |  |  |  |  |  | 0.712 |
| Unfavorable | 1 [Reference] | NA |  | 1 [Reference] | NA |  |
| Intermediate | 0.85 (0.68-1.06) | 0.142 |  | 0.78 (0.65-0.93) | 0.006 |  |
| Favorable | 0.59 (0.47-0.74) | <0.001 |  | 0.62 (0.50-0.76) | <0.001 |  |
| Weighted healthy lifestyle score |  |  |  |  |  | 0.867 |
| Unfavorable | 1 [Reference] | NA |  | 1 [Reference] | NA |  |
| Intermediate | 0.67 (0.54-0.83) | <0.001 |  | 0.66 (0.55-0.79) | <0.001 |  |
| Favorable | 0.51 (0.40-0.64) | <0.001 |  | 0.46 (0.38-0.57) | <0.001 |  |
| PRS and unweighted healthy lifestyle score |  |  |  |  |  | 0.880 |
| Low |  |  |  |  |  |  |
| favorable | 0.53 (0.33-0.84) | 0.006 |  | 0.57 (0.34-0.96) | 0.035 |  |
| Intermediate | 1.03 (0.68-1.56) | 0.871 |  | 0.76 (0.48-1.20) | 0.243 |  |
| unfavorable | 0.95 (0.61-1.49) | 0.821 |  | 0.95 (0.64-1.41) | 0.799 |  |
| Intermediate |  |  |  |  |  |  |
| favorable | 1 [Reference] | NA |  | 1 [Reference] | NA |  |
| Intermediate | 1.20 (0.81-1.79) | 0.362 |  | 1.25 (0.83-1.89) | 0.278 |  |
| unfavorable | 1.54 (1.04-2.27) | 0.032 |  | 1.84 (1.28-2.64) | <0.001 |  |
| High |  |  |  |  |  |  |
| favorable | 1.41 (0.99-2.01) | 0.055 |  | 1.68 (1.13-2.50) | 0.011 |  |
| Intermediate | 1.98 (1.39-2.82) | <0.001 |  | 2.08 (1.43-3.03) | <0.001 |  |
| unfavorable | 2.50 (1.76-3.54) | <0.001 |  | 2.46 (1.74-3.50) | <0.001 |  |
| PRS and weighted healthy lifestyle score |  |  |  |  |  | 0.866 |
| Low |  |  |  |  |  |  |
| favorable | 0.59 (0.35-1.01) | 0.052 |  | 0.48 (0.30-0.78) | 0.003 |  |
| Intermediate | 0.81 (0.50-1.30) | 0.375 |  | 0.73 (0.48-1.10) | 0.131 |  |
| unfavorable | 1.19 (0.77-1.85) | 0.427 |  | 1.02 (0.69-1.49) | 0.931 |  |
| Intermediate |  |  |  |  |  |  |
| favorable | 1 [Reference] | NA |  | 1 [Reference] | NA |  |
| Intermediate | 1.02 (0.65-1.60) | 0.936 |  | 1.25 (0.87-1.80) | 0.221 |  |
| unfavorable | 1.92 (1.29-2.86) | 0.001 |  | 1.81 (1.29-2.55) | <0.001 |  |
| High |  |  |  |  |  |  |
| favorable | 1.35 (0.88-2.07) | 0.165 |  | 1.20 (0.82-1.75) | 0.339 |  |
| Intermediate | 2.07 (1.40-3.06) | <0.001 |  | 1.84 (1.31-2.59) | <0.001 |  |
| unfavorable | 2.66 (1.82-3.89) | <0.001 |  | 2.95 (2.15-4.06) | <0.001 |  |
| Abbreviations: PC, pancreatic cancer; PRS, polygenic risk score; HR, hazard ratio; SD, standard deviation; NA, not applicable. | | | | | | |
| ^a^ Model was adjusted for age (continuous), education level, socioeconomic status, and the first 5 principal components of ancestry. | | | | | | |

| **Table S19. Multivariable Cox regression analysis of lifestyle components in relation to risk of PC, stratified by sex ^a^** | | | | | |
| --- | --- | --- | --- | --- | --- |
| Characteristic | Sex | | | | |
|  | Female | |  | Male | |
|  | HR (95%CI) | *P* value |  | HR (95%CI) | *P* value |
| BMI (kg/m^2^) |  |  |  |  |  |
| Unfavorable | 1 [Reference] | NA |  | 1 [Reference] | NA |
| Intermediate | 0.84 (0.67-1.05) | 0.121 |  | 0.79 (0.67-0.95) | 0.011 |
| Favorable | 0.70 (0.55-0.88) | 0.003 |  | 0.68 (0.54-0.85) | <0.001 |
| Waist circumference (cm) |  |  |  |  |  |
| Unfavorable | 1 [Reference] | NA |  | 1 [Reference] | NA |
| Intermediate | 0.82 (0.65-1.03) | 0.082 |  | 0.79 (0.65-0.95) | 0.013 |
| Favorable | 0.79 (0.64-0.97) | 0.028 |  | 0.65 (0.54-0.79) | <0.001 |
| Physical activity 10+ min (days/week) |  |  |  |  |  |
| Unfavorable | 1 [Reference] | NA |  | 1 [Reference] | NA |
| Intermediate | 0.87 (0.65-1.16) | 0.341 |  | 0.95 (0.74-1.21) | 0.661 |
| Favorable | 0.88 (0.66-1.19) | 0.415 |  | 0.84 (0.66-1.08) | 0.181 |
| Sedentary time (hours/day) |  |  |  |  |  |
| Unfavorable | 1 [Reference] | NA |  | 1 [Reference] | NA |
| Intermediate | 0.76 (0.62-0.93) | 0.008 |  | 0.86 (0.73-1.02) | 0.090 |
| Favorable | 0.78 (0.60-1.02) | 0.068 |  | 0.70 (0.53-0.92) | 0.012 |
| Fruit and vegetable intake |  |  |  |  |  |
| Unfavorable | 1 [Reference] | NA |  | 1 [Reference] | NA |
| Intermediate | 0.96 (0.76-1.21) | 0.738 |  | 0.86 (0.73-1.03) | 0.099 |
| Favorable | 0.89 (0.69-1.14) | 0.338 |  | 0.88 (0.72-1.07) | 0.200 |
| Whole grains intake |  |  |  |  |  |
| Unfavorable | 1 [Reference] | NA |  | 1 [Reference] | NA |
| Intermediate | 0.80 (0.65-0.98) | 0.029 |  | 0.96 (0.76-1.21) | 0.740 |
| Favorable | 0.74 (0.57-0.96) | 0.022 |  | 0.91 (0.72-1.15) | 0.438 |
| Red meat and processed meat intake |  |  |  |  |  |
| Unfavorable | 1 [Reference] | NA |  | 1 [Reference] | NA |
| Intermediate | 1.09 (0.88-1.37) | 0.432 |  | 0.99 (0.84-1.18) | 0.950 |
| Favorable | 0.86 (0.67-1.09) | 0.201 |  | 0.90 (0.73-1.12) | 0.337 |
| Alcohol intake frequency |  |  |  |  |  |
| Unfavorable | 1 [Reference] | NA |  | 1 [Reference] | NA |
| Intermediate | 1.01 (0.79-1.29) | 0.945 |  | 0.78 (0.66-0.93) | 0.006 |
| Favorable | 0.93 (0.71-1.21) | 0.586 |  | 0.94 (0.76-1.17) | 0.595 |
| Smoking |  |  |  |  |  |
| Unfavorable | 1 [Reference] | NA |  | 1 [Reference] | NA |
| Intermediate | 0.52 (0.39-0.70) | <0.001 |  | 0.58 (0.46-0.72) | <0.001 |
| Favorable | 0.46 (0.35-0.61) | <0.001 |  | 0.49 (0.39-0.62) | <0.001 |
| Abbreviations: PC, pancreatic cancer; BMI, body mass index; HR, hazard ratio; NA, not applicable. | | | | | |
| ^a^ Model was adjusted for age (continuous), education level, socioeconomic status, and the first 5 principal components of ancestry. | | | | | |

| **Table S20. The absolute risk reduction of pancreatic cancer in various age and sex groups** | | | | | | | |
| --- | --- | --- | --- | --- | --- | --- | --- |
| Characteristic | Weighted healthy lifestyle score | | | | | | |
|  | Unfavorable | Intermediate | Absolute risk reduction |  | Unfavorable | Favorable | Absolute risk reduction |
|  | events/No. (%) | events/No. (%) | (95%CI) |  | events/No. (%) | events/No. (%) | (95%CI) |
| Age |  |  |  |  |  |  |  |
| ≤60 | 81/71,494 (0.11) | 38/70,672 (0.05) | 0.06 (0.03-0.09) |  | 81/71,494 (0.11) | 31/75,530 (0.04) | 0.07 (0.04-0.10) |
| 61-70 | 221/92,702 (0.24) | 149/93,792 (0.16) | 0.08 (0.04-0.12) |  | 221/92,702 (0.24) | 104/88,996 (0.12) | 0.12 (0.08-0.16) |
| >70 | 224/56,671 (0.40) | 174/58,974 (0.30) | 0.10 (0.03-0.17) |  | 224/56,671 (0.40) | 107/52,936 (0.20) | 0.19 (0.13-0.26) |
| Sex |  |  |  |  |  |  |  |
| Female | 213/58,355 (0.37) | 148/58,607 (0.25) | 0.11 (0.05-0.18) |  | 213/58,355 (0.37) | 109/58,235 (0.19) | 0.18 (0.12-0.24) |
| Male | 313/55,076 (0.57) | 213/55,251 (0.39) | 0.18 (0.10-0.26) |  | 313/55,076 (0.57) | 133/55,107 (0.24) | 0.33 (0.25-0.40) |
| Characteristic | Unweighted healthy lifestyle score | | | | | | |
|  | Unfavorable | Intermediate | Absolute risk reduction |  | Unfavorable | Favorable | Absolute risk reduction |
|  | events/No. (%) | events/No. (%) | (95%CI) |  | events/No. (%) | events/No. (%) | (95%CI) |
| Age |  |  |  |  |  |  |  |
| ≤60 | 75/72,037 (0.10) | 41/65,091 (0.06) | 0.04 (0.01-0.07) |  | 75/72,037 (0.10) | 34/80,568 (0.04) | 0.06 (0.03-0.09) |
| 61-70 | 212/96,657 (0.22) | 143/82,783 (0.17) | 0.05 (0.01-0.09) |  | 212/96,657 (0.22) | 119/96,050 (0.12) | 0.10 (0.06-0.13) |
| >70 | 222/60,058 (0.37) | 155/50,902 (0.30) | 0.07 (-0.003-0.13) | | 222/60,058 (0.37) | 128/57,621 (0.22) | 0.15 (0.09-0.21) |
| Sex |  |  |  |  |  |  |  |
| Female | 156/44,499 (0.35) | 158/53,212 (0.30) | 0.05 (-0.02-0.13) |  | 156/44,499 (0.35) | 156/77,486 (0.20) | 0.15 (0.09-0.21) |
| Male | 353/72,106 (0.49) | 181/49,034 (0.37) | 0.12 (0.05-0.19) |  | 353/72,106 (0.49) | 125/44,294 (0.28) | 0.21 (0.14-0.28) |
| Abbreviations: PC, pancreatic cancer. | | | | | | | |

| **Table S21. The additive interaction between lifestyles and PRS (RERI)^a^** | | | | | | | | |
| --- | --- | --- | --- | --- | --- | --- | --- | --- |
| Characteristic | PRS^b^ | | | | |  | PRS^c^ | |
|  | Intermediate | |  | High | |  | High | |
|  | RERI | 95%Cl |  | RERI | 95%Cl |  | RERI | 95%Cl |
| Weighted healthy lifestyle score |  |  |  |  |  |  |  |  |
| Intermediate | -0.15 | -0.88,0.58 |  | 0.85 | 0.07,1.63 |  | 0.52 | 0.07,0.96 |
| Unfavorable | 0.55 | -0.24,1.33 |  | 1.92 | 0.98,2.86 |  | 0.71 | 0.21,1.22 |
| Unweighted healthy lifestyle score |  |  |  |  |  |  |  |  |
| Intermediate | -0.19 | -0.95,0.57 |  | 0.31 | -0.54,1.15 |  | 0.27 | -0.21,0.76 |
| Unfavorable | 0.58 | -0.12,1.28 |  | 0.97 | 0.15,1.79 |  | 0.19 | -0.31,0.69 |
| BMI (kg/m^2^) |  |  |  |  |  |  |  |  |
| Intermediate | -0.004 | -0.60,0.60 |  | 0.41 | -0.25,1.08 |  | 0.23 | -0.17,0.63 |
| Unfavorable | 0.12 | -0.63,0.87 |  | 0.38 | -0.46,1.22 |  | 0.13 | -0.37,0.63 |
| Waist circumference (cm) |  |  |  |  |  |  |  |  |
| Intermediate | 0.005 | -0.58,0.59 |  | 0.25 | -0.42,0.91 |  | 0.14 | -0.29,0.57 |
| Unfavorable | 0.27 | -0.30,0.85 |  | 0.82 | 0.17,1.48 |  | 0.33 | -0.11,0.76 |
| Physical activity 10+ min (days/week) |  |  |  |  |  |  |  |  |
| Intermediate | 0.08 | -0.43,0.59 |  | -0.40 | -1.03,0.23 |  | -0.28 | -0.68,0.13 |
| Unfavorable | 0.15 | -0.72,1.02 |  | -0.11 | -1.12,0.90 |  | -0.15 | -0.80,0.49 |
| Sedentary time (hours/day) |  |  |  |  |  |  |  |  |
| Intermediate | -0.23 | -1.07,0.62 |  | 0.78 | 0.03,1.54 |  | 0.47 | 0.08,0.87 |
| Unfavorable | -0.43 | -1.32,0.47 |  | 0.91 | 0.13,1.69 |  | 0.58 | 0.20,0.97 |
| Total fruit and vegetable intake |  |  |  |  |  |  |  |  |
| Intermediate | -0.16 | -0.72,0.40 |  | 0.13 | -0.49,0.75 |  | 0.17 | -0.23,0.57 |
| Unfavorable | 0.41 | -0.15,0.97 |  | 0.24 | -0.43,0.90 |  | -0.13 | -0.60,0.34 |
| Whole grains intake |  |  |  |  |  |  |  |  |
| Intermediate | 0.11 | -0.39,0.62 |  | 0.14 | -0.45,0.72 |  | 0.02 | -0.37,0.40 |
| Unfavorable | -0.05 | -0.75,0.65 |  | 0.10 | -0.69,0.88 |  | 0.11 | -0.39,0.61 |
| Red meat and processed meat intake | |  |  |  |  |  |  |  |
| Intermediate | 0.41 | -0.13,0.95 |  | 0.39 | -0.27,1.05 |  | -0.02 | -0.53,0.48 |
| Unfavorable | 0.25 | -0.31,0.80 |  | 0.02 | -0.66,0.70 |  | -0.16 | -0.66,0.34 |
| Alcohol intake frequency |  |  |  |  |  |  |  |  |
| Intermediate | 0.05 | -0.47,0.57 |  | -0.28 | -0.92,0.37 |  | -0.21 | -0.63,0.22 |
| Unfavorable | 0.1 | -0.51,0.71 |  | 0.35 | -0.35,1.05 |  | 0.17 | -0.29,0.64 |
| Smoking |  |  |  |  |  |  |  |  |
| Intermediate | 0.05 | -0.45,0.55 |  | 0.51 | -0.06,1.07 |  | 0.24 | -0.11,0.59 |
| Unfavorable | 0.05 | -1.13,1.23 |  | 2.11 | 0.64,3.59 |  | 1.13 | 0.29,1.97 |
| Abbreviations: PRS, polygenic risk score; RERI, relative excess risk due to interaction; NA, not applicable. | | | | | | | | |
| ^a^ Model was adjusted for age (continuous), sex, education level, socioeconomic status, and the first 5 principal components of ancestry. | | | | | | | | |
| ^b^Note: RERI used individuals with a low PRS and favorable lifestyle as the reference group. | | | | | | | | |
| ^c^Note: RERI used individuals with an intermediate PRS and favorable lifestyle as the reference group. | | | | | | | | |

| **Table S22. The additive interaction between lifestyles, or PRS, and age (RERI) ^a^** | | | | | |
| --- | --- | --- | --- | --- | --- |
| Characteristics | Age | | | | |
|  | 61-70 | |  | >70 | |
|  | RERI | 95%Cl |  | RERI | 95%Cl |
| PRS^b^ |  |  |  |  |  |
| Intermediate | 1.96 | 0.39,3.54 |  | 4.70 | 1.88,7.51 |
| High | 4.25 | 2.13,6.36 |  | 6.52 | 3.12,9.93 |
| PRS^c^ |  |  |  |  |  |
| High | 1.60 | 0.52,2.68 |  | 1.11 | -0.58,2.80 |
| Weighted healthy lifestyle score^d^ |  |  |  |  |  |
| Intermediate | 1.01 | -0.30,2.32 |  | 2.92 | 0.52,5.31 |
| Unfavorable | 2.11 | 0.50,3.72 |  | 4.66 | 1.89,7.43 |
| Unweighted healthy lifestyle score^d^ |  |  |  |  |  |
| Intermediate | 0.85 | -0.38,2.09 |  | 2.04 | -0.15,4.23 |
| Unfavorable | 1.11 | -0.26,2.48 |  | 2.71 | 0.42,5.01 |
| PRS and weighted healthy lifestyle score^e^ |  |  |  |  |  |
| High and intermediate | 4.28 | -0.32,8.89 |  | 6.47 | -1.37,14.31 |
| High and unfavorable | 3.42 | -1.29,8.13 |  | 12.61 | 1.11,24.11 |
| PRS and unweighted healthy lifestyle score^e^ |  |  |  |  |  |
| High and intermediate | 2.12 | -0.28,4.51 |  | 2.45 | -1.54,6.43 |
| High and unfavorable | 3.52 | 0.68,6.36 |  | 5.83 | 0.87,10.80 |
| Abbreviations: PRS, polygenic risk score; RERI, relative excess risk due to interaction; NA, not applicable. | | | | | |
| ^a^ Model was adjusted for sex, education level, socioeconomic status, and the first 5 principal components of ancestry. | | | | | |
| ^b^Note: RERI used individuals with a low PRS and age ≤ 60 as the reference group. | | | | | |
| ^c^Note: RERI used individuals with an intermediate PRS and age ≤ 60 as the reference group. | | | | | |
| ^d^Note: RERI used individuals with a favorable lifestyle and age ≤ 60 as the reference group. | | | | | |
| ^e^Note: RERI used individuals with an intermediate PRS, a favorable lifestyle, and age ≤ 60 as the reference group. | | | | | |

| **Table S23. The additive interaction between lifestyles, or PRS, and sex (RERI) ^a^** | | |
| --- | --- | --- |
| Characteristics | Sex | |
|  | Male | |
|  | RERI | 95%Cl |
| PRS^b^ |  |  |
| Intermediate | 0.55 | 0.08,1.03 |
| High | 0.82 | 0.28,1.36 |
| PRS^c^ |  |  |
| High | 0.19 | -0.21,0.59 |
| Weighted healthy lifestyle score^d^ |  |  |
| Intermediate | 0.24 | -0.19,0.68 |
| Unfavorable | 0.56 | 0.08,1.05 |
| Unweighted healthy lifestyle score^d^ |  |  |
| Intermediate | -0.07 | -0.52,0.39 |
| Unfavorable | 0.18 | -0.27,0.63 |
| PRS and weighted healthy lifestyle score^e^ |  |  |
| High and intermediate | 0.18 | -0.69,1.04 |
| High and unfavorable | 1.16 | 0.20,2.12 |
| PRS and unweighted healthy lifestyle score^e^ |  |  |
| High and intermediate | 0.41 | -0.42,1.25 |
| High and unfavorable | 0.34 | -0.51,1.19 |
| Abbreviations: PRS, polygenic risk score; RERI, relative excess risk due to interaction; NA, not applicable. | | |
| ^a^ Model was adjusted for age (continuous), education level, socioeconomic status, and the first 5 principal components of ancestry. | | |
| ^b^Note: RERI used individuals with a low PRS and female as the reference group. | | |
| ^c^Note: RERI used individuals with an intermediate PRS and female as the reference group. | | |
| ^d^Note: RERI used individuals with a favorable lifestyle and female as the reference group. | | |
| ^e^Note: RERI used individuals with an intermediate PRS, a favorable lifestyle, and female as the reference group. | | |

| **Table S24. Associations between lifestyles, PRS, and PC after excluding the incidence of PC or death during the first 2 years of follow-up** | | | | | |
| --- | --- | --- | --- | --- | --- |
| Characteristic | Model 1^a^ | |  | Model 2^a^ | |
|  | HR (95% CI) | *P* value |  | HR (95% CI) | *P* value |
| BMI (kg/m^2^) |  |  |  |  |  |
| Unfavorable | 1 [Reference] | NA |  | 1 [Reference] | NA |
| Intermediate | 0.85 (0.73-0.98) | 0.027 |  | 0.83 (0.71-0.96) | 0.010 |
| Favorable | 0.60 (0.51-0.71) | <0.001 |  | 0.69 (0.59-0.82) | <0.001 |
| Waist circumference (cm) |  |  |  |  |  |
| Unfavorable | 1 [Reference] | NA |  | 1 [Reference] | NA |
| Intermediate | 0.81 (0.69-0.94) | 0.005 |  | 0.82 (0.71-0.96) | 0.011 |
| Favorable | 0.61 (0.52-0.70) | <0.001 |  | 0.71 (0.61-0.82) | <0.001 |
| Physical activity 10+ min (days/week) |  |  |  |  |  |
| Unfavorable | 1 [Reference] | NA |  | 1 [Reference] | NA |
| Intermediate | 0.95 (0.77-1.15) | 0.584 |  | 0.93 (0.76-1.14) | 0.485 |
| Favorable | 0.91 (0.74-1.11) | 0.333 |  | 0.87 (0.71-1.06) | 0.166 |
| Sedentary time (hours/day) |  |  |  |  |  |
| Unfavorable | 1 [Reference] | NA |  | 1 [Reference] | NA |
| Intermediate | 0.70 (0.62-0.81) | <0.001 |  | 0.79 (0.69-0.91) | <0.001 |
| Favorable | 0.62 (0.51-0.75) | <0.001 |  | 0.76 (0.63-0.93) | 0.007 |
| Total fruit and vegetable intake |  |  |  |  |  |
| Unfavorable | 1 [Reference] | NA |  | 1 [Reference] | NA |
| Intermediate | 0.96 (0.83-1.11) | 0.592 |  | 0.92 (0.79-1.06) | 0.245 |
| Favorable | 0.95 (0.81-1.12) | 0.548 |  | 0.88 (0.75-1.03) | 0.117 |
| Whole grains intake |  |  |  |  |  |
| Unfavorable | 1 [Reference] | NA |  | 1 [Reference] | NA |
| Intermediate | 1.03 (0.88-1.20) | 0.733 |  | 0.88 (0.75-1.03) | 0.111 |
| Favorable | 1.08 (0.91-1.28) | 0.373 |  | 0.81 (0.68-0.96) | 0.016 |
| Red meat and processed meat intake | |  |  |  |  |
| Unfavorable | 1 [Reference] | NA |  | 1 [Reference] | NA |
| Intermediate | 1.00 (0.87-1.15) | 0.96 |  | 1.04 (0.90-1.19) | 0.607 |
| Favorable | 0.76 (0.65-0.89) | <0.001 |  | 0.87 (0.74-1.02) | 0.095 |
| Alcohol intake frequency |  |  |  |  |  |
| Unfavorable | 1 [Reference] | NA |  | 1 [Reference] | NA |
| Intermediate | 0.76 (0.66-0.88) | <0.001 |  | 0.89 (0.77-1.04) | 0.137 |
| Favorable | 0.79 (0.67-0.94) | 0.006 |  | 0.91 (0.77-1.09) | 0.298 |
| Smoking |  |  |  |  |  |
| Unfavorable | 1 [Reference] | NA |  | 1 [Reference] | NA |
| Intermediate | 0.72 (0.60-0.86) | <0.001 |  | 0.56 (0.46-0.67) | <0.001 |
| Favorable | 0.50 (0.42-0.60) | <0.001 |  | 0.48 (0.40-0.58) | <0.001 |
| Unweighted healthy lifestyle score |  |  |  |  |  |
| Unfavorable | 1 [Reference] | NA |  | 1 [Reference] | NA |
| Intermediate | 0.75 (0.65-0.87) | <0.001 |  | 0.82 (0.71-0.94) | 0.006 |
| Favorable | 0.52 (0.45-0.60) | <0.001 |  | 0.60 (0.52-0.71) | <0.001 |
| Weighted healthy lifestyle score |  |  |  |  |  |
| Unfavorable | 1 [Reference] | NA |  | 1 [Reference] | NA |
| Intermediate | 0.68 (0.59-0.79) | <0.001 |  | 0.68 (0.59-0.78) | <0.001 |
| Favorable | 0.46 (0.40-0.54) | <0.001 |  | 0.50 (0.43-0.59) | <0.001 |
| PRS |  |  |  |  |  |
| Low | 0.61 (0.51-0.73) | <0.001 |  | 0.61 (0.51-0.72) | <0.001 |
| Intermediate | 1 [Reference] | NA |  | 1 [Reference] | NA |
| High | 1.43 (1.24-1.64) | <0.001 |  | 1.43 (1.25-1.65) | <0.001 |
| Abbreviations: PC, pancreatic cancer; PRS, polygenic risk score; BMI, body mass index; HR, hazard ratio; NA, not applicable. | | | | | |
| ^a^ Model 1 was not adjusted; model 2 was adjusted for age (continuous), sex, education level, socioeconomic status, and the first 5 principal components of ancestry. | | | | | |

| **Table S25. Combined analysis of PRS and lifestyles on the risk of PC in participants after excluding the incidence of PC or death during the first 2 years of follow-up** | | | | | | | | |
| --- | --- | --- | --- | --- | --- | --- | --- | --- |
| Characteristic | PRS | | | | | | | |
|  | Low | |  | Intermediate | |  | High | |
|  | HR (95% CI) | *P* value |  | HR (95% CI) | *P* value |  | HR (95% CI) | *P* value |
| Weighted healthy lifestyle score |  |  |  |  |  |  |  |  |
| Favorable | 0.57 (0.40-0.82) | 0.002 |  | 1 [Reference] | NA |  | 1.24 (0.92-1.66) | 0.158 |
| Intermediate | 0.78 (0.57-1.08) | 0.131 |  | 1.16 (0.87-1.56) | 0.306 |  | 1.86 (1.42-2.43) | <0.001 |
| Unfavorable | 1.10 (0.81-1.48) | 0.542 |  | 1.86 (1.42-2.44) | <0.001 |  | 2.65 (2.05-3.41) | <0.001 |
| Unweighted healthy lifestyle score |  |  |  |  |  |  |  |  |
| Favorable | 0.57 (0.40-0.80) | 0.001 |  | 1 [Reference] | NA |  | 1.40 (1.07-1.85) | 0.016 |
| Intermediate | 0.88 (0.64-1.21) | 0.423 |  | 1.20 (0.90-1.61) | 0.216 |  | 1.93 (1.48-2.52) | <0.001 |
| Unfavorable | 0.95 (0.70-1.27) | 0.716 |  | 1.69 (1.30-2.20) | <0.001 |  | 2.27 (1.77-2.92) | <0.001 |
| BMI (kg/m^2^) |  |  |  |  |  |  |  |  |
| Favorable | 0.59 (0.42-0.82) | 0.002 |  | 1 [Reference] | NA |  | 1.34 (1.02-1.76) | 0.037 |
| Intermediate | 0.67 (0.50-0.89) | 0.007 |  | 1.09 (0.84-1.42) | 0.511 |  | 1.73 (1.35-2.21) | <0.001 |
| Unfavorable | 0.90 (0.65-1.25) | 0.537 |  | 1.43 (1.08-1.91) | 0.013 |  | 1.86 (1.42-2.44) | <0.001 |
| Waist circumference (cm) |  |  |  |  |  |  |  |  |
| Favorable | 0.61 (0.45-0.82) | 0.001 |  | 1 [Reference] | NA |  | 1.34 (1.05-1.72) | 0.018 |
| Intermediate | 0.72 (0.52-0.98) | 0.036 |  | 1.09 (0.83-1.44) | 0.534 |  | 1.64 (1.27-2.10) | <0.001 |
| Unfavorable | 0.79 (0.59-1.06) | 0.115 |  | 1.37 (1.07-1.77) | 0.013 |  | 2.01 (1.59-2.53) | <0.001 |
| Physical activity 10+ min (days/week) |  |  |  |  |  |  |  |  |
| Favorable | 0.58 (0.44-0.76) | <0.001 |  | 1 [Reference] | NA |  | 1.55 (1.25-1.92) | <0.001 |
| Intermediate | 0.71 (0.55-0.91) | 0.008 |  | 1.14 (0.91-1.43) | 0.267 |  | 1.52 (1.22-1.88) | <0.001 |
| Unfavorable | 0.78 (0.51-1.19) | 0.249 |  | 1.16 (0.81-1.65) | 0.415 |  | 1.65 (1.21-2.25) | 0.002 |
| Sedentary time (hours/day) |  |  |  |  |  |  |  |  |
| Favorable | 0.49 (0.30-0.79) | 0.004 |  | 1 [Reference] | NA |  | 0.96 (0.65-1.43) | 0.858 |
| Intermediate | 0.46 (0.32-0.66) | <0.001 |  | 0.86 (0.62-1.19) | 0.358 |  | 1.25 (0.91-1.71) | 0.161 |
| Unfavorable | 0.69 (0.49-0.96) | 0.027 |  | 0.99 (0.72-1.36) | 0.961 |  | 1.54 (1.14-2.08) | 0.005 |
| Total fruit and vegetable intake |  |  |  |  |  |  |  |  |
| Favorable | 0.61 (0.44-0.85) | 0.004 |  | 1 [Reference] | NA |  | 1.39 (1.06-1.82) | 0.018 |
| Intermediate | 0.66 (0.49-0.88) | 0.005 |  | 0.93 (0.71-1.22) | 0.592 |  | 1.55 (1.21-1.98) | <0.001 |
| Unfavorable | 0.63 (0.46-0.87) | 0.005 |  | 1.24 (0.95-1.63) | 0.119 |  | 1.53 (1.18-1.99) | 0.001 |
| Whole grains intake |  |  |  |  |  |  |  |  |
| Favorable | 0.60 (0.44-0.81) | <0.001 |  | 1 [Reference] | NA |  | 1.41 (1.10-1.80) | 0.007 |
| Intermediate | 0.61 (0.46-0.81) | <0.001 |  | 1.05 (0.82-1.35) | 0.671 |  | 1.59 (1.27-2.00) | <0.001 |
| Unfavorable | 0.84 (0.60-1.18) | 0.318 |  | 1.24 (0.92-1.66) | 0.158 |  | 1.63 (1.24-2.14) | <0.001 |
| Red meat and processed meat intake | |  |  |  |  |  |  |  |
| Favorable | 0.69 (0.48-0.99) | 0.044 |  | 1 [Reference] | NA |  | 1.58 (1.18-2.13) | 0.002 |
| Intermediate | 0.72 (0.52-0.99) | 0.047 |  | 1.32 (0.99-1.75) | 0.062 |  | 1.88 (1.43-2.47) | <0.001 |
| Unfavorable | 0.79 (0.58-1.09) | 0.151 |  | 1.27 (0.95-1.69) | 0.110 |  | 1.71 (1.30-2.26) | <0.001 |
| Alcohol intake frequency |  |  |  |  |  |  |  |  |
| Favorable | 0.62 (0.44-0.88) | 0.007 |  | 1 [Reference] | NA |  | 1.47 (1.12-1.94) | 0.006 |
| Intermediate | 0.64 (0.48-0.85) | 0.002 |  | 1.02 (0.79-1.33) | 0.880 |  | 1.38 (1.07-1.77) | 0.012 |
| Unfavorable | 0.62 (0.44-0.88) | 0.007 |  | 1.09 (0.81-1.47) | 0.571 |  | 1.69 (1.29-2.22) | <0.001 |
| Smoking |  |  |  |  |  |  |  |  |
| Favorable | 0.59 (0.45-0.76) | <0.001 |  | 1 [Reference] | NA |  | 1.31 (1.07-1.62) | 0.010 |
| Intermediate | 0.65 (0.50-0.85) | 0.002 |  | 1.12 (0.89-1.41) | 0.340 |  | 1.58 (1.28-1.96) | <0.001 |
| Unfavorable | 1.26 (0.87-1.83) | 0.219 |  | 1.63 (1.16-2.28) | 0.005 |  | 3.11 (2.37-4.06) | <0.001 |
| Abbreviations: PC, pancreatic cancer; PRS, polygenic risk score; BMI, body mass index; HR, hazard ratio; NA, not applicable. | | | | | | | | |
| Model was adjusted for age (continuous), sex, education level, socioeconomic status, and the first 5 principal components of ancestry. | | | | | | | | |

| **Table S26. Associations between lifestyles, PRS, and PC using competing risk analysis** | | | | | |
| --- | --- | --- | --- | --- | --- |
| Characteristic | Model 1^a^ | |  | Model 2^a^ | |
|  | HR (95% CI) | *P* value |  | HR (95% CI) | *P* value |
| BMI (kg/m^2^) |  |  |  |  |  |
| Unfavorable | 1 [Reference] | NA |  | 1 [Reference] | NA |
| Intermediate | 0.84 (0.73-0.97) | 0.016 |  | 0.82 (0.72-0.95) | 0.006 |
| Favorable | 0.61 (0.52-0.71) | <0.001 |  | 0.69 (0.59-0.82) | <0.001 |
| Waist circumference (cm) |  |  |  |  |  |
| Unfavorable | 1 [Reference] | NA |  | 1 [Reference] | NA |
| Intermediate | 0.79 (0.69-0.92) | 0.002 |  | 0.81 (0.70-0.94) | 0.005 |
| Favorable | 0.61 (0.53-0.70) | <0.001 |  | 0.72 (0.62-0.82) | <0.001 |
| Physical activity 10+ min (days/week) |  |  |  |  |  |
| Unfavorable | 1 [Reference] | NA |  | 1 [Reference] | NA |
| Intermediate | 0.94 (0.78-1.14) | 0.530 |  | 0.93 (0.77-1.13) | 0.460 |
| Favorable | 0.91 (0.75-1.10) | 0.330 |  | 0.88 (0.72-1.06) | 0.190 |
| Sedentary time (hours/day) |  |  |  |  |  |
| Unfavorable | 1 [Reference] | NA |  | 1 [Reference] | NA |
| Intermediate | 0.74 (0.65-0.84) | <0.001 |  | 0.82 (0.72-0.94) | 0.003 |
| Favorable | 0.62 (0.51-0.74) | <0.001 |  | 0.75 (0.62-0.91) | 0.004 |
| Total fruit and vegetable intake |  |  |  |  |  |
| Unfavorable | 1 [Reference] | NA |  | 1 [Reference] | NA |
| Intermediate | 0.95 (0.83-1.09) | 0.490 |  | 0.91 (0.79-1.04) | 0.160 |
| Favorable | 0.96 (0.82-1.12) | 0.590 |  | 0.88 (0.75-1.03) | 0.100 |
| Whole grains intake |  |  |  |  |  |
| Unfavorable | 1 [Reference] | NA |  | 1 [Reference] | NA |
| Intermediate | 1.00 (0.86-1.17) | 0.960 |  | 0.87 (0.74-1.01) | 0.063 |
| Favorable | 1.08 (0.92-1.27) | 0.340 |  | 0.82 (0.70-0.97) | 0.021 |
| Red meat and processed meat intake | |  |  |  |  |
| Unfavorable | 1 [Reference] | NA |  | 1 [Reference] | NA |
| Intermediate | 1.01 (0.88-1.15) | 0.880 |  | 1.04 (0.91-1.19) | 0.570 |
| Favorable | 0.77 (0.66-0.90) | 0.001 |  | 0.88 (0.75-1.03) | 0.099 |
| Alcohol intake frequency |  |  |  |  |  |
| Unfavorable | 1 [Reference] | NA |  | 1 [Reference] | NA |
| Intermediate | 0.74 (0.64-0.85) | <0.001 |  | 0.86 (0.75-0.99) | 0.038 |
| Favorable | 0.79 (0.67-0.93) | 0.004 |  | 0.90 (0.77-1.06) | 0.210 |
| Smoking |  |  |  |  |  |
| Unfavorable | 1 [Reference] | NA |  | 1 [Reference] | NA |
| Intermediate | 0.74 (0.62-0.88) | <0.001 |  | 0.58 (0.49-0.69) | <0.001 |
| Favorable | 0.52 (0.44-0.61) | <0.001 |  | 0.50 (0.42-0.60) | <0.001 |
| Unweighted healthy lifestyle score |  |  |  |  |  |
| Unfavorable | 1 [Reference] | NA |  | 1 [Reference] | NA |
| Intermediate | 0.76 (0.66-0.87) | <0.001 |  | 0.82 (0.71-0.94) | 0.004 |
| Favorable | 0.53 (0.46-0.61) | <0.001 |  | 0.61 (0.53-0.71) | <0.001 |
| Weighted healthy lifestyle score |  |  |  |  |  |
| Unfavorable | 1 [Reference] | NA |  | 1 [Reference] | NA |
| Intermediate | 0.68 (0.60-0.78) | <0.001 |  | 0.68 (0.59-0.78) | <0.001 |
| Favorable | 0.46 (0.40-0.54) | <0.001 |  | 0.50 (0.42-0.58) | <0.001 |
| PRS |  |  |  |  |  |
| Low | 0.60 (0.50-0.70) | <0.001 |  | 0.59 (0.50-0.70) | <0.001 |
| Intermediate | 1 [Reference] | NA |  | 1 [Reference] | NA |
| High | 1.51 (1.32-1.72) | <0.001 |  | 1.51 (1.33-1.73) | <0.001 |
| Abbreviations: PC, pancreatic cancer; PRS, polygenic risk score; BMI, body mass index; HR, hazard ratio; NA, not applicable. | | | | | |
| ^a^ Model 1 was not adjusted; model 2 was adjusted for age (continuous), sex, education level, socioeconomic status, and the first 5 principal components of ancestry. | | | | | |

| **Table S27. Combined analysis of PRS and lifestyle components on the risk of PC using competing risk analysis** | | | | | | | | |
| --- | --- | --- | --- | --- | --- | --- | --- | --- |
| Characteristic | PRS | | | | | | | |
|  | Low | |  | Intermediate | |  | High | |
|  | HR (95% CI) | *P* value |  | HR (95% CI) | *P* value |  | HR (95% CI) | *P* value |
| Weighted healthy lifestyle score | |  |  |  |  |  |  |  |
| Favorable | 0.53 (0.37-0.76) | <0.001 |  | 1 [Reference] | NA |  | 1.26 (0.95-1.68) | 0.100 |
| Intermediate | 0.76 (0.55-1.03) | 0.078 |  | 1.15 (0.87-1.53) | 0.320 |  | 1.92 (1.49-2.49) | <0.001 |
| Unfavorable | 1.06 (0.80-1.42) | 0.690 |  | 1.81 (1.39-2.34) | <0.001 |  | 2.77 (2.17-3.54) | <0.001 |
| Unweighted healthy lifestyle score | |  |  |  |  |  |  |  |
| Favorable | 0.55 (0.39-0.77) | <0.001 |  | 1 [Reference] | NA |  | 1.52 (1.17-1.98) | 0.002 |
| Intermediate | 0.88 (0.65-1.20) | 0.410 |  | 1.22 (0.92-1.62) | 0.170 |  | 2.02 (1.56-2.61) | <0.001 |
| Unfavorable | 0.92 (0.69-1.23) | 0.590 |  | 1.69 (1.31-2.19) | <0.001 |  | 2.41 (1.89-3.07) | <0.001 |
| BMI (kg/m^2^) |  |  |  |  |  |  |  |  |
| Favorable | 0.50 (0.35-0.70) | <0.001 |  | 1 [Reference] | NA |  | 1.45 (1.12-1.89) | 0.005 |
| Intermediate | 0.68 (0.51-0.89) | 0.005 |  | 1.11 (0.86-1.44) | 0.410 |  | 1.79 (1.41-2.27) | <0.001 |
| Unfavorable | 0.81 (0.59-1.11) | 0.490 |  | 1.41 (1.07-1.86) | 0.014 |  | 2.01 (1.55-2.60) | <0.001 |
| Waist circumference (cm) |  |  |  |  |  |  |  |  |
| Favorable | 0.60 (0.45-0.80) | <0.001 |  | 1 [Reference] | NA |  | 1.44 (1.14-1.81) | 0.002 |
| Intermediate | 0.68 (0.50-0.92) | 0.013 |  | 1.09 (0.84-1.43) | 0.510 |  | 1.67 (1.31-2.13) | <0.001 |
| Unfavorable | 0.78 (0.59-1.03) | 0.077 |  | 1.35 (1.06-1.72) | 0.017 |  | 2.11 (1.69-2.63) | <0.001 |
| Physical activity 10+ min (days/week) | |  |  |  |  |  |  |  |
| Favorable | 0.58 (0.45-0.76) | <0.001 |  | 1 [Reference] | NA |  | 1.71 (1.39-2.10) | <0.001 |
| Intermediate | 0.71 (0.55-0.91) | 0.008 |  | 1.17 (0.94-1.46) | 0.160 |  | 1.60 (1.31-1.97) | <0.001 |
| Unfavorable | 0.73 (0.48-1.11) | 0.140 |  | 1.23 (0.88-1.72) | 0.230 |  | 1.77 (1.32-2.38) | <0.001 |
| Sedentary time (hours/day) |  |  |  |  |  |  |  |  |
| Favorable | 0.47 (0.29-0.76) | 0.002 |  | 1 [Reference] | NA |  | 1.04 (0.71-1.52) | 0.830 |
| Intermediate | 0.46 (0.32-0.67) | <0.001 |  | 0.89 (0.64-1.23) | 0.470 |  | 1.41 (1.04-1.91) | 0.026 |
| Unfavorable | 0.69 (0.50-0.96) | 0.026 |  | 1.02 (0.75-1.39) | 0.890 |  | 1.63 (1.21-2.19) | 0.001 |
| Total fruit and vegetable intake | |  |  |  |  |  |  |  |
| Favorable | 0.61 (0.44-0.85) | 0.003 |  | 1 [Reference] | NA |  | 1.52 (1.17-1.97) | 0.002 |
| Intermediate | 0.66 (0.49-0.87) | 0.004 |  | 0.95 (0.73-1.23) | 0.690 |  | 1.63 (1.28-2.07) | <0.001 |
| Unfavorable | 0.62 (0.45-0.85) | 0.003 |  | 1.27 (0.98-1.66) | 0.074 |  | 1.66 (1.29-2.14) | <0.001 |
| Whole grains intake |  |  |  |  |  |  |  |  |
| Favorable | 0.59 (0.44-0.80) | <0.001 |  | 1 [Reference] | NA |  | 1.52 (1.20-1.92) | <0.001 |
| Intermediate | 0.60 (0.46-0.78) | <0.001 |  | 1.07 (0.84-1.36) | 0.580 |  | 1.60 (1.28-1.99) | <0.001 |
| Unfavorable | 0.80 (0.57-1.10) | 0.170 |  | 1.17 (0.88-1.56) | 0.290 |  | 1.80 (1.39-2.33) | <0.001 |
| Red meat and processed meat intake | |  |  |  |  |  |  |  |
| Favorable | 0.67 (0.47-0.96) | 0.027 |  | 1 [Reference] | NA |  | 1.69 (1.27-2.24) | <0.001 |
| Intermediate | 0.70 (0.51-0.96) | 0.028 |  | 1.31 (0.99-1.74) | 0.055 |  | 1.98 (1.53-2.58) | <0.001 |
| Unfavorable | 0.78 (0.57-1.06) | 0.110 |  | 1.27 (0.96-1.67) | 0.095 |  | 1.80 (1.38-2.35) | <0.001 |
| Alcohol intake frequency |  |  |  |  |  |  |  |  |
| Favorable | 0.61 (0.44-0.85) | 0.003 |  | 1 [Reference] | NA |  | 1.58 (1.21-2.04) | <0.001 |
| Intermediate | 0.61 (0.46-0.81) | <0.001 |  | 1.03 (0.80-1.33) | 0.810 |  | 1.40 (1.10-1.78) | 0.007 |
| Unfavorable | 0.61 (0.44-0.86) | 0.005 |  | 1.06 (0.79-1.42) | 0.690 |  | 1.86 (1.44-2.41) | <0.001 |
| Smoking |  |  |  |  |  |  |  |  |
| Favorable | 0.57 (0.44-0.73) | <0.001 |  | 1 [Reference] | NA |  | 1.37 (1.12-1.67) | 0.002 |
| Intermediate | 0.62 (0.48-0.80) | <0.001 |  | 1.08 (0.86-1.35) | 0.510 |  | 1.68 (1.37-2.06) | <0.001 |
| Unfavorable | 1.19 (0.83-1.70) | 0.360 |  | 1.61 (1.17-2.23) | 0.004 |  | 3.03 (2.34-3.92) | <0.001 |
| Abbreviations: PC, pancreatic cancer; PRS, polygenic risk score; BMI, body mass index; HR, hazard ratio; NA, not applicable. | | | | | | | | |
| Model was adjusted for age (continuous), sex, education level, socioeconomic status, and the first 5 principal components of ancestry. | | | | | | | | |


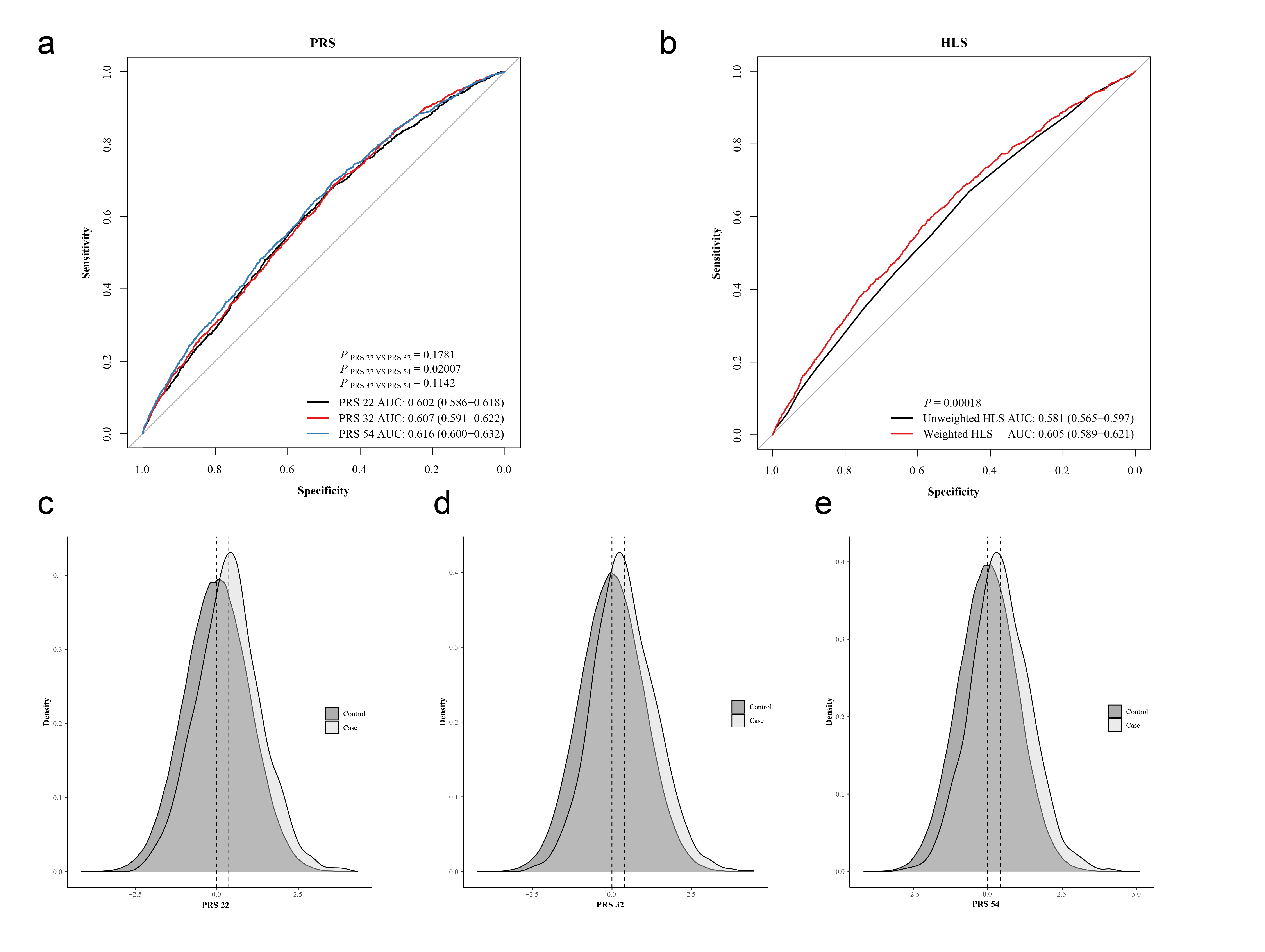


**Fig. S1. ROC curves and density plots of PRS and healthy lifestyle score.** (a) ROC curves, AUC values, and 95% CI for the prediction accuracy of PC by PRS 22, PRS 32 and PRS 54. (b) ROC curves, AUC values, and 95% CI for the prediction accuracy of PC by weighted and unweighted healthy lifestyle scores. (c) (d) (e) Density plots showing the distribution of standardized PRSs in PC cases and non-PC controls.


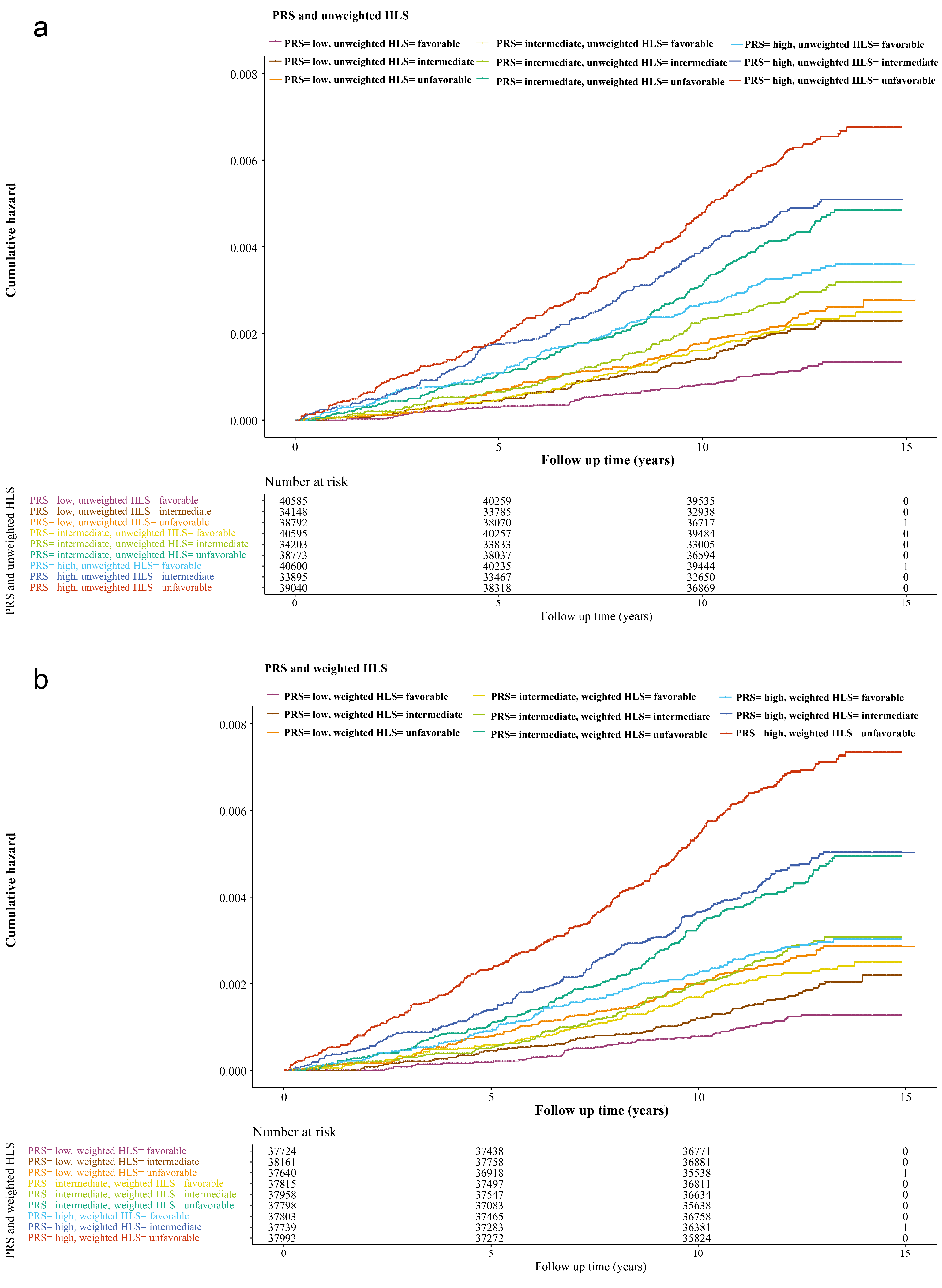


**Fig. S2. Cumulative risk of PC by the joint effect of lifestyle and PRS.** (a) Cumulative risk of PC during follow-up by joint effect of unweighted healthy lifestyle score and PRS; (b) Cumulative risk of PC during follow-up by joint effect of weighted healthy lifestyle score and PRS.


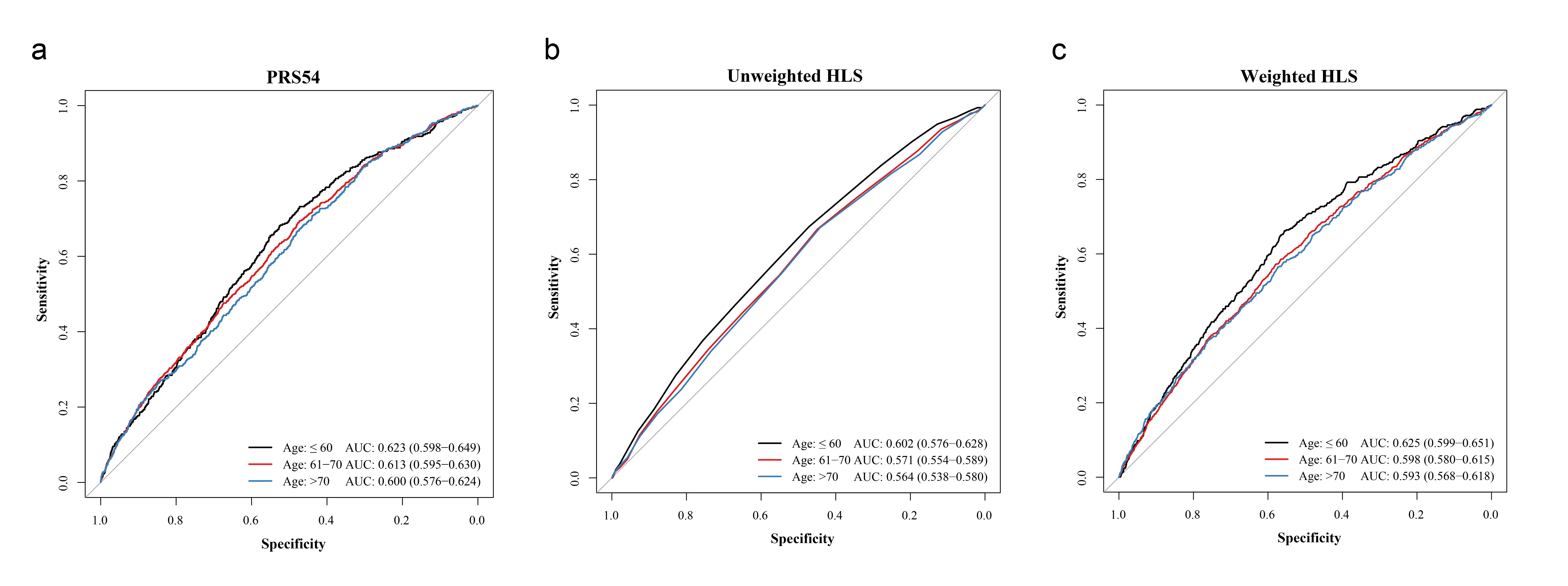


**Fig. S3. ROC curves and AUC metrics of PRS and HLSs according to different age groups.** (a) ROC curves and AUC metrics of PRS; (b) ROC curves and AUC metrics of the unweighted healthy lifestyle score; (c) ROC curves and AUC metrics of the weighted healthy lifestyle score.
